# High Molar Mass Polycarbonates as Closed-Loop Recyclable Thermoplastics

**DOI:** 10.1021/jacs.3c14170

**Published:** 2024-03-14

**Authors:** Gloria Rosetto, Fernando Vidal, Thomas M. McGuire, Ryan W. F. Kerr, Charlotte K. Williams

**Affiliations:** Department of Chemistry, Chemistry Research Laboratory, University of Oxford, 12 Mansfield Rd, Oxford OX1 3TA, U.K.

## Abstract

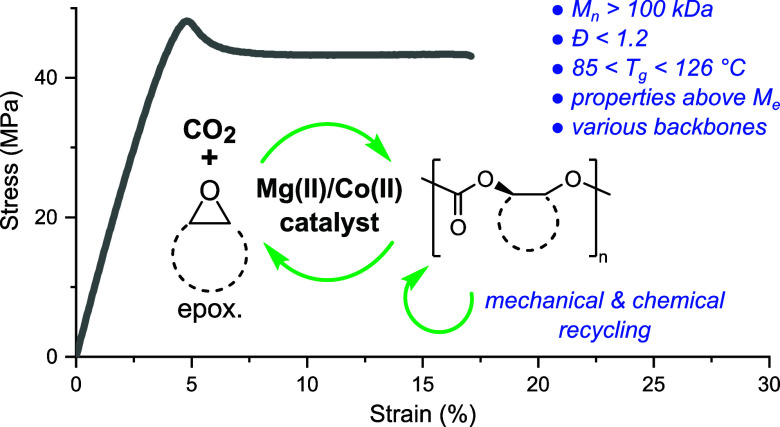

Using carbon dioxide (CO_2_) to make recyclable thermoplastics could reduce greenhouse gas emissions associated with polymer manufacturing. CO_2_/cyclic epoxide ring-opening copolymerization (ROCOP) allows for >30 wt % of the polycarbonate to derive from CO_2_; so far, the field has largely focused on oligocarbonates. In contrast, efficient catalysts for high molar mass polycarbonates are underinvestigated, and the resulting thermoplastic structure–property relationships, processing, and recycling need to be elucidated. This work describes a new organometallic Mg(II)Co(II) catalyst that combines high productivity, low loading tolerance, and the highest polymerization control to yield polycarbonates with number average molecular weight (*M*_n_) values from 4 to 130 kg mol^–1^, with narrow, monomodal distributions. It is used in the ROCOP of CO_2_ with bicyclic epoxides to produce a series of samples, each with *M*_n_ > 100 kg mol^–1^, of poly(cyclohexene carbonate) (PCHC), poly(vinyl-cyclohexene carbonate) (PvCHC), poly(ethyl-cyclohexene carbonate) (PeCHC, by hydrogenation of PvCHC), and poly(cyclopentene carbonate) (PCPC). All these materials are amorphous thermoplastics, with high glass transition temperatures (85 < *T*_g_ < 126 °C, by differential scanning calorimetry) and high thermal stability (*T*_d_ > 260 °C). The cyclic ring substituents mediate the materials’ chain entanglements, viscosity, and glass transition temperatures. Specifically, PCPC was found to have 10× lower entanglement molecular weight (*M*_e_)_n_ and 100× lower zero-shear viscosity compared to those of PCHC, showing potential as a future thermoplastic. All these high molecular weight polymers are fully recyclable, either by reprocessing or by using the Mg(II)Co(II) catalyst for highly selective depolymerizations to epoxides and CO_2_. PCPC shows the fastest depolymerization rates, achieving an activity of 2500 h^–1^ and >99% selectivity for cyclopentene oxide and CO_2_.

## Introduction

Polymers are among the largest scale chemicals produced; they are currently manufactured at a rate of around 400 megatonnes (Mt) annually.^1−3^ Their production from petrochemicals accounts for >1.0 gigatonne of carbon dioxide-equivalents (Gt CO_2_-equiv), representing >60% of overall lifecycle emissions. The total carbon footprint and other environmental pollution is exacerbated by a lack of effective recycling.^3−5^ Two important strategies to address these issues have emerged from several recent independent global plastics systemic models: (1) replace virgin petrochemical feedstocks with carbon dioxide utilization technologies, either directly achieved by CO_2_-based chemistries or through biomass upgrading and (2) drastically increase thermoplastic recycling rates, ensuring all future materials undergo energy-efficient mechanical recycling and/or chemical recycling to monomers.^1,2,6−10^ Our interest is to produce polycarbonate thermoplastics directly using CO_2_, showing useful properties and selective end-life recycling (both mechanical and chemical).^7−9^

CO_2_ and epoxide ring-opening copolymerization (ROCOP) is a front-runner carbon dioxide utilization technology.^11−13^ Most polymerizations apply catalysts to produce hydroxyl telechelic oligocarbonates (1 < *M*_n_ < 20 kg mol^–1^, where *M*_n_ is the number average molecular weight), or CO_2_-polyols, which are important as surfactants and in production of polyurethane foams, elastomers, coatings, sealants, and adhesives.^13−18^ Some excellent catalysts include Co(III) or Al(III) complexes tethered to ionic cocatalysts (often bis(triphenylphosphine)iminium halides, e.g., PPNCl); dimeric/dinuclear complexes, e.g., Zn(II)Zn(II), Zn(II)Mg(II), Co(II)Mg(II), Ln(III)Zn(II), which operate without any cocatalyst; or borane complexes tethered to/used with ionic cocatalysts (ammonium or phosphonium halides).^19−41^ Such CO_2_-polyol catalysts need to be applied with a very large-excess (10–100 equiv vs catalyst) of a protic chain transfer agent (CTA), often a diol, which controls the oligomer chain length and hydroxyl chain end-groups.^42^ Chain transfer reactions are alcoholysis processes, whereby all alcohol groups react rapidly with the growing polymer chains, moving them rapidly on/off the catalyst; these processes generally occur faster than propagation.^27,43,44^ Most catalysts also react with residual water, either in the apparatus or monomers, to ring-open epoxides and generate diols in situ, which are also bifunctional CTAs. With the inclusion of these added (or inadvertent) mono- and bifunctional CTAs, together with catalysts or cocatalysts featuring a monofunctional initiator (usually an acetate or chloride), the polymer molar masses almost inevitably exhibit bimodal distributions.^42,44^ These restrictions on molar masses, where the *M*_n_ is well below the entanglement molecular weight (*M*_e_), and high dispersities (*D̵*) have so far hampered CO_2_-based polymers from effectively challenging petroleum-based commodity thermoplastics. For instance, prior studies of poly(cyclohexene carbonate) (PCHC) have consistently noted its difficult processability and high brittleness.^45,46^

In contrast, catalysts that yield high molar mass polycarbonates (*M*_n_ = 50–150 kg mol^–1^) with narrow, monomodal molar mass distributions are much less common.^38,47−49^ Accessing such materials is desirable since entanglement phenomena directly impact many polymer physical properties, including glass transition temperature (*T*_g_), crystallization, mechanical strength, and processability.^50^ In 2001, Darensbourg and Koning reported upon a moderate molar mass PCHC sample (*M*_n_ = 42 kg mol^–1^, *Đ* = 6), produced using a Zn(II)Zn(II) catalyst, which showed a tensile stress of 43 MPa, Young’s modulus of 3.3 GPa, but only 2% elongation at break.^45^ The authors estimated an *M*_e_ of ∼15 kg mol^–1^ but noted that it was likely an underestimation. In 2022, Frey and Floudas investigated, both experimentally and computationally, the chain dynamics of moderate molar mass PCHC (5 < *M*_n_ < 33 kg mol^–1^) produced using a [(*R*,*R*-salcy)CoCl]/PPNCl catalyst system, estimating an *M*_e_ of 16 kg mol^–1^.^46^ It is also relevant to note that PCHC has been successfully applied as a “rigid” block in various phase separated copolymers (*T*_g_ of PCHC ∼ 110–120 °C), producing adhesives, elastomers, and plastics, depending on the comonomers and overall molar mass values.^15,18,43,51−53^ Beyond CHO as comonomer in ROCOP, the teams of Rieger and Greiner independently focused on related 6-membered ring, biobased poly(limonene carbonate), developing routes to high molar mass and terpolymers with PCHC.^54−56^ Wu and co-workers reported poly(cyclopentene carbonate) (PCPC) with molar masses up to 84 kg mol^–1^, but the analyzed sample (*M*_n_ = 20 kg mol^–1^, *T*_g_ = 70 °C) displayed moderate tensile strength (20 MPa) and no yield point.^48^

Underpinning future progress on CO_2_-based polycarbonates should be informed upon comprehensive material investigations, for instance, by determining the *M*_e_ more accurately. To increase the molar mass, two approaches to synthesize PCHC have been proposed. One consists of attempting to rigorously remove all residual protic impurities and traces of water with highly reactive scavengers; however, such purifications might be rather challenging to scale.^47^ For instance, Jia and co-workers discovered highly active borane/PPNCl catalysts for CHO/CO_2_ ROCOP, which when applied with rigorously dried CO_2_ and extensively purified CHO produced PCHC with *M*_n_ ∼ 450 kg mol^–1^ (*Đ* = 1.31). In 2022, Li and co-workers applied the same triethyl borane with different phosphazene cocatalysts to produce high molar mass PCHC (*M*_n_ ∼ 280 kg mol^–1^, *Đ* = 1.59).^38^ These impressive achievements were not accompanied by investigation of the material properties of the polymers. A second strategy applies organometallic catalysts that react in situ with alcohols to form only one type of initiator (the organometallic ligand does not initiate on its own).^47^ To reach sufficiently high molar masses, such catalysts must show high activity and tolerate impurities at low loadings. Previously, we reported organometallic Zn(II)Zn(II) and Zn(II)Mg(II) catalysts, which exhibit such initiation control.^43,57,58^ Unfortunately, these catalysts were not sufficiently active to produce high molar mass polycarbonates. In 2020, we reported a very active Mg(II)Co(II) acetate catalyst for CHO/CO_2_ ROCOP, which is > 1000× faster than Zn(II)Zn(II) and ∼10× faster than Mg(II)Zn(II).^27^

Any future uses for high molar mass CO_2_-based plastics must also allow for low-energy recycling options—most obviously by mechanical recycling.^44^ In addition, chemical recycling strategies allow for recovery of the constituent monomers, in this case, epoxides and CO_2_.^7,59,60^

Darensbourg and co-workers pioneered CO_2_-derived polycarbonate chemical recycling, describing the first catalysts, applied with a strong base, for the depolymerization of PCPC to cyclopentene oxide and CO_2_.^61^ Subsequently, a range of other catalysts were active for PCHC and other cyclic substituted polycarbonates.^62−68^ Although promising results have been obtained, the understanding and overall performance of such depolymerization catalysis require improvements to maximize energy efficiency.

**Figure 1:**
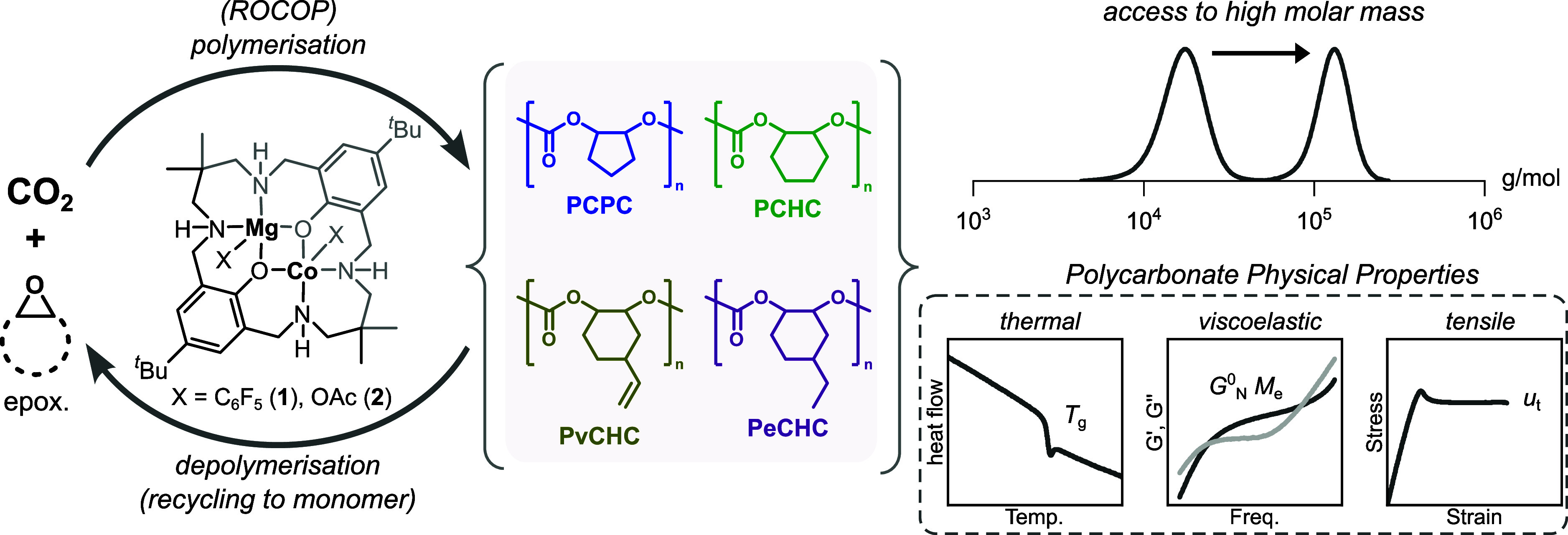
Catalysts and polymers targeted in this work, with the objective of elucidating thermal-mechanical properties and recycling routes for high molarmass cyclic substituted polycarbonates.

Thus, to address these challenges, we propose to employ a novel non-initiating organometallic Mg(II)Co(II) catalyst (Figure 1). When used with 5- and 6-membered cyclic epoxides, the latter including alkyl substituents (vinyl-and ethyl-cyclohexene), high molar mass CO_2_-based polycarbonates with end-group selectivity and narrow dispersity will allow us to elucidate polymer structure–property relationships. Finally, the Mg(II)Co(II) catalyst will also be investigated in depolymerization reactions, targeting high activity, selectivity, minimal catalyst loading, and reducing the operating temperatures for efficient chemical recycling to high-value epoxide monomers and CO_2_.

## Results

### Catalyst Synthesis and Carbon Dioxide ROCOP Catalysis

**Figure 2:**
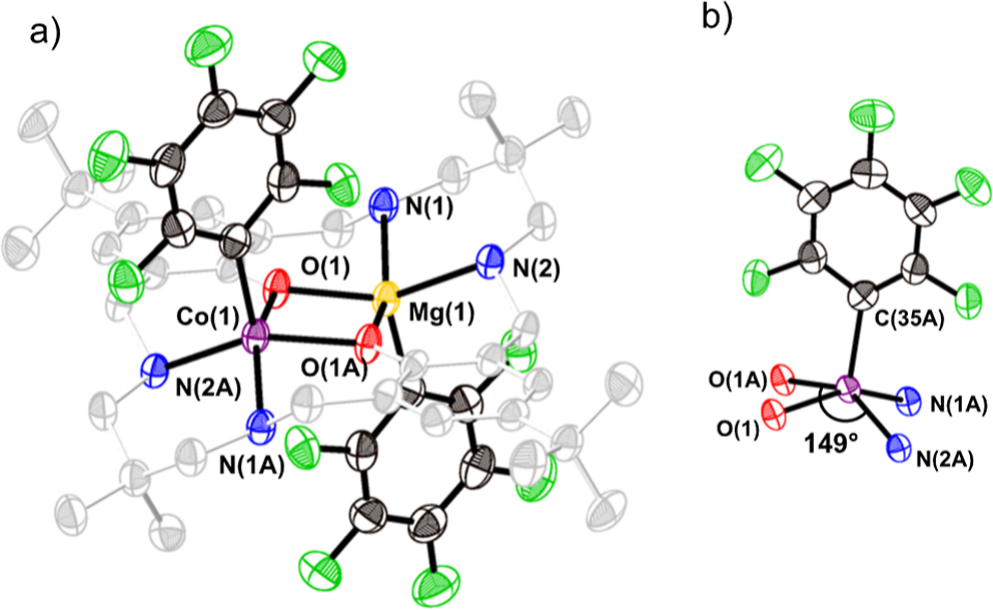
(a) ORTEP diagram for the molecular structure of **1**. H atoms and two THF molecules were omitted for clarity. Thermal ellipsoids are at 50% probability level. Co = purple, Mg = yellow, O = red, N = blue, F = green, C = gray (Figure S1 for full structure, Table S1 for crystallographic details). (b) Section of the crystal structure depicting the square pyramidal geometry around the cobalt center.

To investigate the best catalysts for high molar mass polycarbonate production, the organometallic Mg(II)Co(II) complex (**1**) and previously reported Mg(II)Co(II) diacetate complex (**2**) were compared (Figure 1).^27^ Pentafluorophenyl was selected as the organometallic ligand for its anticipated stability (with respect to β-H elimination), high reactivity of Co–C_6_F_5_ with alcohols to undergo irreversible protonation, and low nucleophilicity of Co–C_6_F_5_ to prevent CO_2_ insertion. These features ensure that the complex is non-initiating, which is essential for high molar mass polymer production. The new organometallic Mg(II)Co(II) catalyst, **1**, was prepared with pentafluorophenyl groups as the hydrolyzable non-initiating coligands. To make the catalyst, the macrocyclic ligand [LH_2_] was first reacted with [Mg{N(SiMe_3_)_2_}_2_], followed by a reaction with cobalt(II) bis(pentafluorophenyl) ([Co(C_6_F_5_)_2_ (THF)_2_]) and stirring the mixture for 24 h.^69,70^ Complex **1**, [LMgCo(C_6_F_5_)_2_], was isolated by cooling the reaction solution to −30 °C, as a purple crystalline solid, in 53% yield. Complex **2** was prepared following literature methods.^27^ Single crystals of **1**, suitable for X-ray diffraction, were isolated via recrystallization, from a saturated THF solution, at −30 °C. Its solid-state structure confirms the formation of a heterodinuclear complex (Figure 2a and Table S1), with the aryl coligands coordinated at each metal and the ligand adopting an “S” shape. In complex **1**, the Co(II) center is pentacoordinate and adopts a distorted square pyramidal geometry with the aryl ligand coordinated at the apex (Figure 2b). The Mg(II)Co(II) separation is 3.069(3) Å (Table S2 for selected bond lengths and angles).

The ^1^H NMR spectrum of complex **1**, in toluene-*d*_8_, shows peaks from −55 to 270 ppm, characteristic of a paramagnetic Co(II) species (Figure S2). Its ^19^F{^1^H} NMR spectrum shows three peaks: two broad singlets at −155 and −65 ppm and a triplet at −154 ppm, the relative integrals are 2:1:1 (Figure S4, see the Supporting Information for a discussion on the characterization of **1**). Complex **1** was also characterized by SQUID magnetometry and cyclic voltammetry (CV). The magnetic moment, arising from Co(II), is 4.99 μ_B,_ which is consistent with a square pyramidal high spin, Co(II) d^7^ complex (Figure S6). Previously, Hoffmann and Rossi investigated the structures for related five-coordinate Co(II) complexes, and **1** shows an electronic configuration consistent with their proposal.^71^ It also shows an irreversible Co(II/III) oxidation at *E*_pa_ = −0.819 V (vs Fc/Fc^+^) and ligand-centered oxidations at *E*_pa_ = 0.39 V (vs Fc/Fc^+^) (Figure S7).

To make high molar mass polycarbonates, it is essential to cleanly control the initiation reaction, and here, this is achieved using an organometallic ligand, which reacts with alcohols (CTAs). To investigate the initiation process further, complex **1** (0.01 mmol) was reacted with 2 equiv of 2-fluorophenol at room temperature in toluene-*d*_8_. The reaction was conducted in a Young’s tap NMR tube and monitored by spectroscopy (Figure S12). The addition of alcohol resulted in an immediate change of color, from pale to a much brighter pink (Figure S13). The resulting ^19^F{^1^H} NMR spectrum showed new resonances at −56 and −132 ppm, corresponding to the clean formation of the new 4-fluorophenoxide ligands coordinated to the Co(II) and Mg(II) centers, respectively. In addition, the product of alcoholysis, HC_6_F_5_, was also detected with a 1:1 relative integration, further confirming the expected reactivity. This small-scale experiment proves that the reaction between complex **1** and alcohols is instantaneous, quantitative, and irreversible. Such reactivity is essential for initiation control during polymerization, which is required to achieve high molar mass polymers.

**Figure 3:**
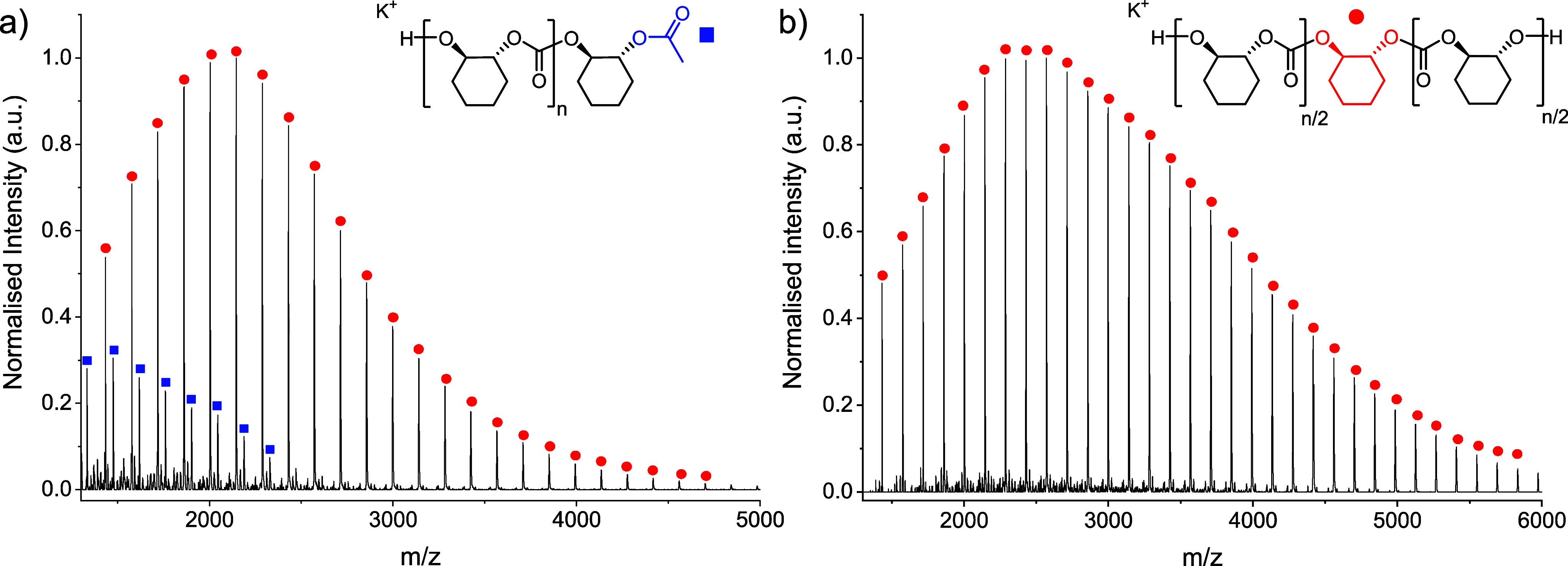
MALDI-ToF spectra for low molar mass samples of PCHC prepared using catalyst **2** (a) or **1** (b). Polymerization conditions: [catalyst]:[CHD]:[CHO] = 1:4:2000, using a solution of 3 M CHO in toluene, 80 °C, quenched after 10 min, conv. ∼5%. Chain distributions that are end-capped by CHD are marked with a red circle, and those by acetate are marked with a blue square. See Figure S14 for end group calculations and *m*/*z* peak annotation of MALDI-ToF spectra.

Next, the control of epoxide/CO_2_ ROCOP with complex **1** was studied using 4 equiv of 1,2-cyclohexane diol (CHD) as CTA and CHO as the comonomer to produce PCHC. MALDI-ToF analysis of a low molar mass PCHC sample, prepared in toluene at 80 °C using a [**1**]:[CHD]:[CHO] ratio of 1:4:2000, pressurized to 1 bar of CO_2_ and quenched at low conversions (10 min, conv. ∼5%), shows a single distribution of α,ω-dihydroxy telechelic polymer chains, initiated solely from CHD (Figure 3b). There was no evidence of any chains initiated by pentafluorophenyl groups or from carboxylates formed by carbon dioxide insertion (Figure S14 for end group calculations). In contrast, complex **2**, under identical conditions, generates polymer chains displaying both α,ω-dihydroxyl and α-acetal-ω-hydroxy end-groups. The two different end-groups result from a mixture of CHD and non-innocent catalyst acetate groups, respectively (Figure 3a). Another structural feature of note is polymer tacticity, which can be determined by quantitative ^13^C NMR spectroscopy.^72^ The spectrum for a PCHC sample catalyzed by **1** showed that the polymer is atactic, with a *P*_m_ value of 0.5 (Figure S15).

**Figure 4:**
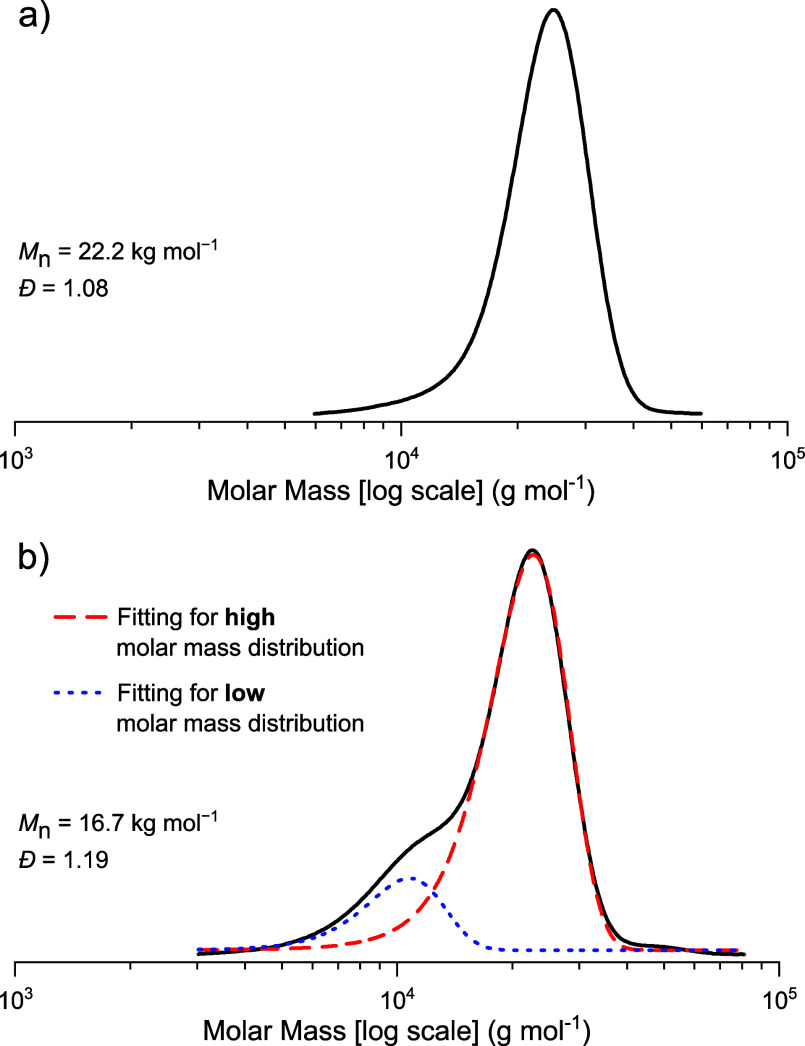
GPC traces of PvCHC (THF as eluent) produced by catalysts **1** (a) and **2** (b), in which case the molar mass peak was deconvoluted into two bimodal distributions as shown by the dashed and point lines. Polymerization conditions: [catalyst]:[CHD]:[vCHO] = 1:4:2000, 1 bar of CO_2_, 100 °C, 1 h.

Next, vinyl-cyclohexene oxide (vCHO)/CO_2_ ROCOP was conducted, at 1 bar pressure, using either catalyst **1** or **2**. The vCHO comonomer was selected since it generally polymerizes at a similar rate to CHO but benefits from an additional alkene substituent on the cyclohexene ring, which might enable postpolymerization functionalization and enable study of pendant alkyl substituents on the dynamics of polymer chains.^17,73−76^ Using [**1**]:[CHD]:[vCHO] of 1:4:2000, at 100 °C and 1 bar of CO_2_ pressure, the reaction reached 30% epoxide conversion within an hour and formed poly(vinyl-cyclohexene carbonate) (PvCHC )with >99% carbonate linkage selectivity (Figure S16). Catalyst **1** shows good activity, with a turnover frequency (TOF) of 600 h^–1^, which is closely comparable to that using **2** (TOF = 660 h^–1^), under identical conditions.^27^ Further, initial rates for these reactions, calculated from CO_2_ mass flow data, are also very similar for catalysts **1** and **2**, with *k*_obs_ values of 4.5 and 4.2 min^–1^, respectively (Figure S17). These findings demonstrate that the coligands (aryl or acetate) do not influence the polymerization propagation kinetics, i.e., the two catalysts feature virtually identical propagating intermediates.^26,43^ The ligands do, however, significantly influence the initiation processes and the resulting molar masses and distributions. Using catalyst **1**, PvCHC has a higher molar mass and monomodal distribution (*M*_n_ = 22.2 kg mol^–1^, *Đ* = 1.08) (Figure 4a), while, under identical conditions, **2** yields polymers with lower molar mass and bimodal distributions (*M*_n_ = 16.7 kg mol ^–1^, *Đ* = 1.19) (Figure 4b). In the case of catalyst **2**, the bimodality can be deconvoluted into a lower molar mass distribution, assigned to acetate-initiated chains, and a higher molar mass distribution, assigned to CHD-initiated polymer chains (Figure 4b).

**Figure 5:**
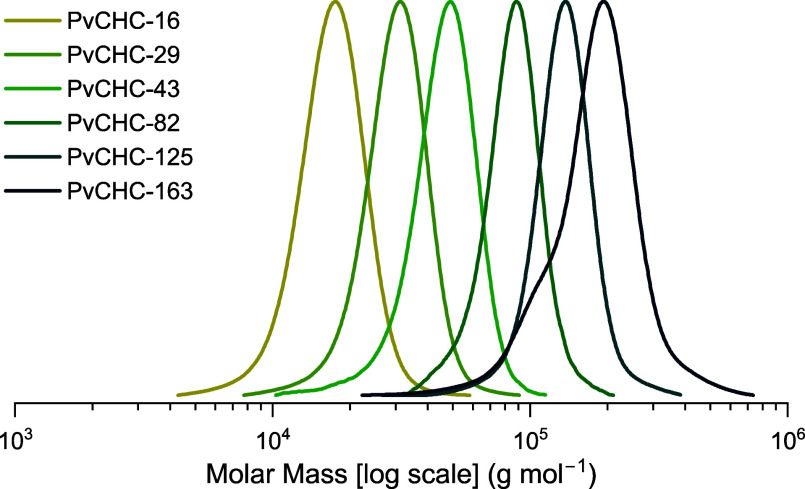
GPC traces (THF as the eluent) of PvCHC samples produced by using catalyst **1** (Table 1). A deconvoluted GPC trace of PvCHC-163 is provided in the Supporting Information (Figure S19) showing that the lower molar mass shoulder accounts for 1.2% of the total distribution.

Catalyst**1** showed improved polymerization control and was therefore applied using a range of different [catalyst]:[epoxide] ratios to prepare a series of high molar mass polycarbonates. The polymerizations were all performed in toluene solutions of the appropriate epoxide (3.0 M) using steel Parr-reactors, at 20 bar CO_2_ pressure; these conditions ensured efficient mixing and stirring particularly as conversion and viscosity increased, enabling maximum productivity (turnover numbers, TON) and high epoxide conversions (Table 1). It is worth noting that typical ROCOP conditions are selected to maximize polymerization rates at low epoxide conversions, where viscosities are low.^77^ On the other hand, conditions to achieve high epoxide conversion, where viscosities are higher and rates are lower, are comparatively underinvestigated. Since metal-catalyzed epoxide/CO_2_ copolymerizations are controlled, high epoxide conversions should result in the highest polymer molar masses. Further, the polymer molar mass should be inversely related to the quantity of CHD added since it functions as both the chain-initiator and chain-transfer agent (Figure S18). To demonstrate the outstanding polymerization control and alcohol tolerance of complex **1**, CO_2_/epoxide ROCOP was conducted using systematically increasing quantities of CHD (4 to 32 equiv) vs **1**. In these investigations, a [**1**]:[vCHO] loading ratio of 1:3000 (0.033 mol % catalyst) was kept constant, and polymerizations all achieved >95% CHO conversion (Table 1, entries 1–4). Notably, the resulting series of PvCHC samples all show very narrow (1.07 < *D̵* < 1.10) monomodal molar mass distributions, which are typical of well-controlled polymerizations (Figure 5). The experimentally determined molar mass values, using GPC against polystyrene standards, are high (*M*_n_ up to 81.9 kg mol^–1^) and inversely related to the quantity of CTA added. The catalyst’s remarkable tolerance and stability at very low concentrations and high alcohol (CHD) loadings are clear from the good correlation between experimental and theoretical molar masses, *M*_n, est_ (see the Supporting Information for further detail). Further decreasing catalyst loadings to 0.017 mol % and even 0.010 mol %, with constant 4 equiv of CHD, resulted in all cases in almost complete conversions (>95%) to PvCHC (Table 1, entries 5–6). These experiments yielded the target high molar masses of 125 and 163 kg mol^–1^, with monomodal and narrow *D̵* (1.06–1.17) (Figure 5). The catalyst’s ability to control the molar mass by varying either the catalyst or diol loading, and to achieve complete conversions, is essential to investigate the thermal-mechanical properties of the resulting materials.

**Table 1:**
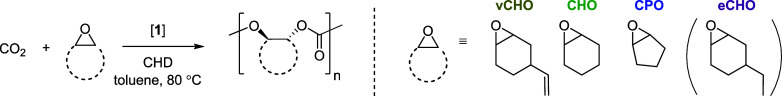
Data for Bicyclic Epoxide/CO_2_ ROCOP Using Catalyst **1**^a^

run (#)	polymer	epoxide	CHD equiv	epoxide equiv	time (h)	epoxide conv. (%)^b^	TON^c^	*M*_n, GPC_ (kg mol^–1)^^d^	*D̵*^e^	*M*_n, est_ (kg mol^–1)^^f^
1	PvCHC-16	vCHO	32	3000	10	95	2850	16.0	1.10	14
2	PvCHC-29	vCHO	16	3000	10	99	2970	28.5	1.09	28
3	PvCHC-43	vCHO	8	3000	8	95	2850	43.4	1.10	48
4	PvCHC-82	vCHO	4	3000	10	99	2970	81.9	1.07	84
5	PvCHC-125	vCHO	4	6000	24	99	5940	124.5	1.06	126
6	PvCHC-163	vCHO	4	10,000	30	98	9800	162.8	1.17	153
7	PCHC-122	CHO	4	10,000	18	95	9500	121.6	1.09	333^g^
8	PCPC-114	CPO	4	10,000	40	96	9600	114.4	1.06	320^g^
9	PeCHC-125	eCHO						72.6	1.34	125

a Polymerizations were all conducted at 80 °C, 20 bar CO_2_ in toluene (3 M solutions of epoxide); PeCHC was produced by hydrogenation of PvCHC (see the Supporting Information for details), monomer structure shown for comparison.

b Determined by ^1^H NMR spectroscopy by comparing the integrals of the resonances for the epoxide vs those of the polymer (Figures S24–S26).

c TON = (moles of epoxide converted)/(moles of catalyst).

d Determined by GPC, in THF, calibrated using narrow polystyrene standards.

e *Đ* = *M*_w_/*M*_n_, determined by GPC.

f *M*_n, est_ = [(epoxide conv.)(epoxide equiv)(MW repeat unit)] + [(CHD equiv)(MW CHD)]/(CHD + *x*) (where *x* = residual CTA, see the Supporting Information for further information).

g *M*_n, est_ = [(epoxide conv.)(epoxide equiv)(MW repeat unit)] + [(CHD equiv)(MW CHD)]/(CHD).

Having optimized the reaction conditions to reach high molar masses with 0.010 mol % loadings of catalyst **1**, the ROCOP catalysis was extended using cyclohexene oxide (CHO) or cyclopentene oxide (CPO). Similarly, high epoxide conversions, at 80 °C in toluene, produced high molar mass polycarbonates (Table 1, entries 7–8). Specifically, CHO produced PCHC with *M*_n_ = 121.6 kg mol^–1^ and very narrow *Đ* = 1.09 (Figure S20), while CPO produced PCPC with *M*_n_ = 114.4 kg mol^–1^ and *Đ* = 1.06 (Figure S21). Despite these high molar masses, in these cases, there are discrepancies between the experimental and theoretical values, which are attributed to the use of GPC standards and/or different quantities of residual diols in the epoxides.

### Hydrogenation of vinyl groups in PvCHC to PeCHC

Finally, to expand our study on the effect of ring size and substituents in CO_2_-derived polycarbonates, the potential to modify the alkene of PvCHC was explored. Hydrogenation of the vinyl group was selected since it will yield ethyl substituents on the cyclohexane rings; alkylated polymers are known to be more easily processed. Following a modified literature procedure, PvCHC-125 was reacted with *para*-toluenesulfonyl hydrazide and formed the corresponding alkylated polymer, PeCHC-125, in good yield (68% following repeated precipitation from MeOH).^78^ The hydrogenation reaction did not significantly change the polymer backbone, although there was some low molar mass tailing via GPC (*M*_n_ = 72.6 kg mol^–1^, *Đ* = 1.34) (Figures S22 and S23).

### Polycarbonate’s Thermal-Mechanical Properties

Any attempts to use these higher-molar-mass polycarbonates as thermoplastics require insights into their thermal and viscoelastic properties since these will control the temperature ranges over which they can be processed and used. The series of ∼120 kg mol^–1^ CO_2_-polycarbonates were each characterized by differential scanning calorimetry (DSC), thermogravimetric analysis (TGA), and oscillatory shear rheology (Table 3). All the samples are amorphous plastics showing high glass transition temperatures (*T*_g_) with values, as determined by DSC, in the range 85–126 °C, which are dependent on the ring-size and alkyl-substituents (Figure S27a). As such, the *T*_g_ for PCHC, featuring the 6-membered ring (*T*_g,DSC_ = 126 °C, which is at the limit predicted by the Fox–Flory equation),^46^ was ∼40 °C higher than for PCPC, which features the 5-membered ring (*T*_g,DSC_ = 85 °C).^79^ Substituted rings show greater segmental motion compared to nonsubstituted ones, i.e., added pendant alkyl chains resulted in a slight reduction of the *T*_g_ values. Specifically, PvCHC (vinyl-substituted) and PeCHC (ethyl-substituted) showed *T*_g_ values of 118 and 105 °C, respectively, which are 8–25 °C lower than PCHC. As expected, the polymer molar mass influences the glass transition temperature, with values increasing with molar mass, e.g., PvCHC with *M*_n_ 16 kg mol^–1^ shows a glass transition temperature of 112 °C, whereas the sample with *M*_n_ 82 kg mol^–1^ shows a *T*_g_ of 118 °C (Figure S28).

Another method to measure the glass transition temperature is from the peak in tan(δ) obtained during variable temperature oscillatory rheology (Figures S31–S38). Analysis of the samples using this method reveals the same trend of increasing glass transition temperatures depending on the ring sizes and pendent alkyl chains, with values increasing in the order PCPC < PeCHC < PvCHC < PCHC. Compared with those measured by DSC, *T*_g_’s determined by rheology are all slightly higher. In addition, all polymers show the necessary high temperature stability, with the onset of thermal decomposition occurring at temperatures (*T*_d_) above 265 °C (Figure S27b). These results indicate a wide processing temperature range, from 135 to 250 °C in all cases. It is noted that the high-temperature stability was dependent upon effective purification to remove residual catalyst, achieved by precipitation and silica-plug filtration (see the Supporting Information for details).

Further insights into the polymers’ molecular dynamics and extent of entanglement were obtained from the time and temperature data acquired by oscillatory shear rheology in the melt (Table 2). During temperature ramps (0.5% strain, 1.0 Hz, 2 °C·min^–1^), all the polycarbonates display four typical viscoelastic regions: a glassy state, a glass transition, a rubbery plateau, and viscous flow, whose characteristic temperatures and moduli values depend on the backbone ring size and alkyl substituents (Figures S31, S33, S35, and S37). First, only small variations were observed in the high glassy plateau moduli at the start of the oscillatory temperature investigations, with *G*′ ∼ 450–460 MPa. Next, the glass transitions are marked by a sharp drop in the viscoelastic moduli and a subsequent peak in the tan(δ), which varied from 97 °C for PCPC to ∼130 °C for PvCHC and PCHC. The crossover temperature is the point at which *G*′ = *G*″ and marks the end of the rubbery plateau. These are lowest for PeCHC and PCPC at 161–166 °C and increase to 192–196 °C for PCHC and a stabilized sample of PvCHC (0.1 wt % of a radical inhibitor, pentaerythritol tetrakis (3,5-di-*tert*-butyl-4-hydroxyhydrocinnamate), PEHC).

**Table 2:** Thermal and Viscoelastic Properties of the High Molar Mass Polycarbonates^a^

polymer	*T*_g, DSC_^c^ (°C)	*T*_g, rheo_^d^ (°C)	*T*_t,cross_^e^ (°C)	*T*_d_^f^ (°C)^b^	*G*_glass_′^g^ (MPa)	*G*_N_^0^^h^ (MPa)	*M*_e_^i^ (kg mol^–1^)	τ_*t*_^j^ (min)	η_0_^k^ (MPa s)
PCPC-114	85	97	166	271	453	0.65 ± 0.02	3.4–4.6	0.21 ± 0.02	0.78 ± 0.09
PeCHC-125	105	118	161	306	447	0.170 ± 0.004	13–18	0.57 ± 0.03	1.26 ± 0.11
PvCHC-125^b^	118	129	196	277	459	0.049 ± 0.017	45–62	87 ± 18	30 ± 20
PCHC-122	126	132	192	265	453	0.056 ± 0.011	56	240 ± 30	90 ± 20

a Time–temperature superposition (TTS) master curves were referenced at 140 °C (see the Supporting Information for experimental details and calculations, Figures S31–S38).

b Sample was stabilized with 0.1 wt % of a radical inhibitor, pentaerythritol tetrakis (3,5-di-*tert*-butyl-4-hydroxyhydrocinnamate) (PEHC).

c Measured by DSC from the second scan at a heating rate of 10 °C·min^–1^ (Figure S27a).

d Measured by oscillatory shear rheology from the peak in the tan(δ) curve obtained during temperature ramp experiments at heating rates of 2 °C·min^–1^ (Figures S31, S33, S35 and S37).

e Terminal regime crossover temperature obtained by oscillatory shear rheology during temperature ramp experiments, 2 °C·min^–1^.

f Onset of thermal decomposition, as measured by TGA (Figure S27b).

g Low-temperature glassy plateau modulus obtained by oscillatory shear rheology during temperature ramp experiments, 2 °C·min^–1^ (Figures S31, S33, S35 and S37).

h Mid frequency rubbery plateau modulus in the TTS master curves (Figures S32, S34, S36, and S38).

i Entanglement molecular weight, as determined from the plateau modulus, *G*_N_^0^, located at the minimum of the tan(δ) curves in the TTS master curves.

j Terminal relaxation time obtained as τ_*t*_ = ω^–1^ at which *G*′ = *G*″ in the TTS master curves.

k Zero shear viscosity, obtained from the low-frequency plateau of the TTS master curves (Figures S32, S34, S36 and S38).

**Figure 6:**
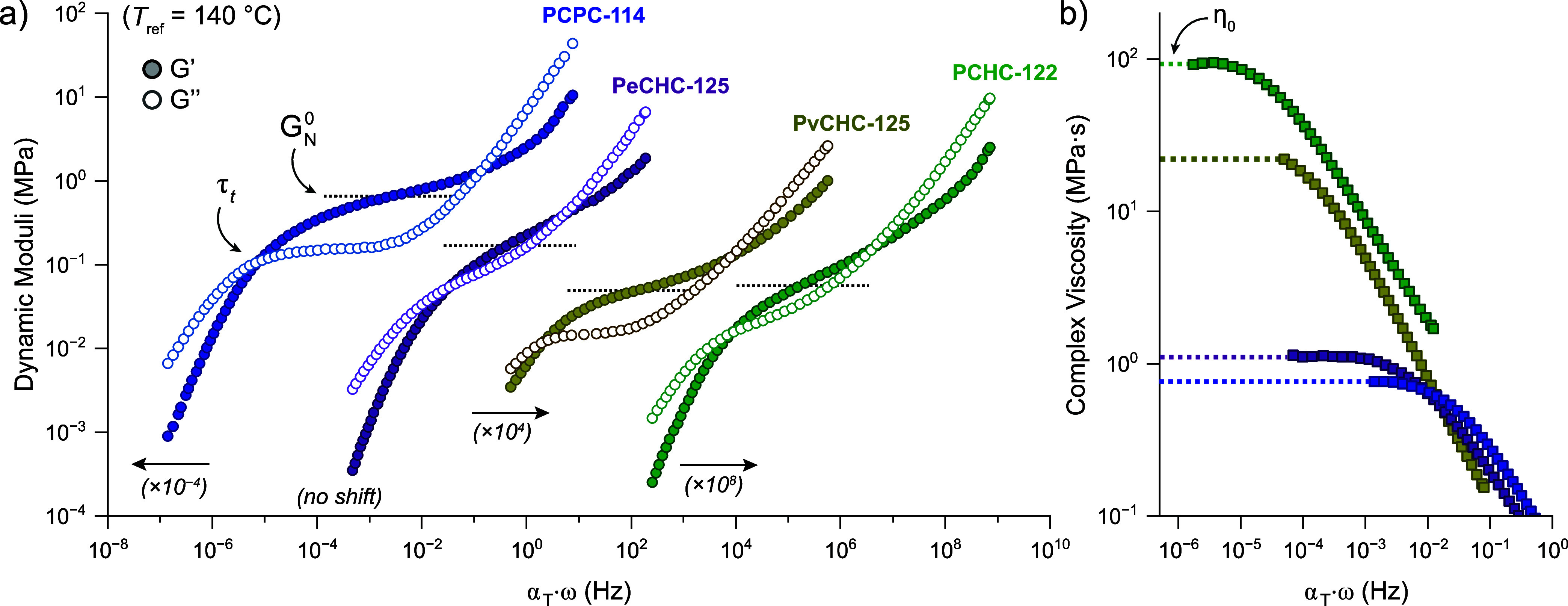
Rheological characterization of high molar mass polycarbonates: PCPC-114 (blue), PeCHC-120 (purple), PvCHC-125 (containing 0.1 wt % of PEHC as the thermal stabilizer, dark yellow), and PCHC-122 (green). (a) Plot of dynamic moduli (*G*′ and *G*″) vs frequency (Hz), obtained from TTS master curves (curves have been shifted horizontally for clarity, and the shift factors applied to each master curve are specified in the plot; for unshifted TTS master curves see the Supporting Information). (b) Plot of shear viscosity vs frequency (Hz) obtained from TTS master curves and projection of the plateau viscosity to zero-shear.

To further probe the influence of the polymer structure on the molecular dynamics, the samples were analyzed by variable frequency rheology, at constant 10 °C intervals, and the data were used to construct TTS master curves (Figure 6a,b and S32, S34, S36 and S38, reference temperature, *T*_ref_, set at 140 °C). In these investigations, PvCHC-125 was stabilized with 0.1 wt % of PEHC to prevent cross-linking (Figure S47). The shift factors, *a*_T_, showed excellent fits to the Williams–Landel–Ferry equation (Table S4 and Figures S39–S46). Similar to the observations in the temperature ramps, all the polycarbonates display typical thermoplastic behavior: their viscoelastic response changes from the glassy state at high frequencies to viscous flow at low frequencies, passing through a rubbery plateau at intermediate frequencies. There are, however, some notable differences in the viscoelastic parameters within the series of materials, signaling the significant influence of the polymer backbone structure on both the slow (long-distance chain entanglements) and fast (short-distance segmental rearrangements, i.e., molecular motions corresponding to the monomer repeat unit) relaxation processes. In the transition zone, both of the dynamic moduli (*G*′ and *G*″) show a strong frequency dependence. This results in a characteristic maximum in the tan(δ) data, which correlates approximately with the middle of the transition zone. The values for tan(δ)_max_ occur at the lowest frequencies for PCHC (4.3 Hz), which is 1 order of magnitude lower than for PeCHC and PvCHC (33–70 Hz) and 4 orders of magnitude lower than for PCPC (42000 Hz). The longer times needed for PCHC suggest that its cyclohexyl groups exert a greater resistance to molecular motions than alkyl-substituted rings and those with one fewer carbon atom. The resistance may arise from conformational effects, for example, the cyclohexene chair conformations may move more slowly through the polymer matrix than other repeat units.^46^

At intermediate relaxation times, the decrease of storage and loss moduli with frequency is less severe, giving rise to a plateau region in *G*′. In this region, the polymer viscoelastic properties are influenced by chain entanglements that restrain long-range motions. The rubbery plateau modulus, *G*_N_^0^, can be determined from the minimum of the tan(δ) in this region, allowing for the estimation of *M*_e_ using the tube model equation
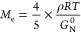
where ρ is the polymer density (see the Supporting Information for estimates), *R* is the gas constant, and *T* is the temperature.^80,81^ Importantly, the best performing thermoplastics typically show low values of *M*_e_. The comparison of this series of polymers reveals that the cyclohexyl ring polymers, PCHC and PvCHC, both show *M*_e_ values that are >10 times higher (∼54–56 kg mol^–1^) than the cyclopentyl ring-substituted polymer, PCPC (*M*_e_ ∼ 4.0 kg mol^–1^). Due to the high molar masses and narrow dispersity values of the polymers described in this work, values of *M*_e_ obtained for PCHC are 4 times greater than those previously estimated.^45,46^ Intriguingly, ethyl-substituted PeCHC displays a somewhat intermediate value of *M*_e_ ∼ 15 kg mol^–1^ between these two extremes. It is clear from these results that ring-size and alkyl substitution directly influence the molecular dynamics, including the distance between entanglements.

Finally, at shorter frequencies and after modulus crossover, the materials begin to undergo a Newtonian flow. In this region, the relaxation time, τ, of the bulk material is correlated to the crossover frequency where τ = 1/ω at the point where *G*′ = *G*″ (Tables 2, and S4). In the series of polymers, PCHC shows the longest relaxation times (4 h) followed by PvCHC (∼1.5 h), while PeCHC and PCPC had comparatively fast relaxations (0.2–1 min). In this region, the complex viscosity also reaches a plateau, i.e., the zero shear viscosity, η_0_. At the reference temperatures, PCPC and PeCHC show 115× and 70× lower η_0_ values than PCHC, another beneficial property for polymer processing.

**Figure 7:**
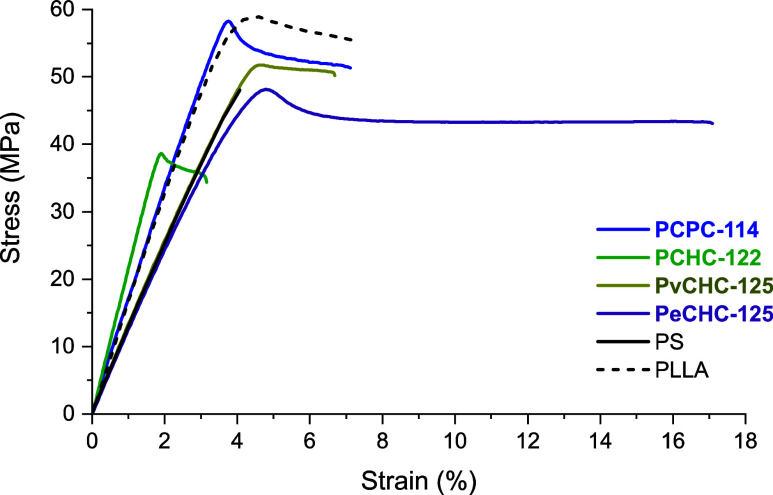
Representative stress vs strain data for high molar mass polycarbonates, dumbbell specimens by ISO 527, test speed of 10 mm·min^–1^ (full data in Figures S55–S58).

Films of the polymers (PvCHC-125, PCHC-122, PCPC-114, and PeCHC-125) were prepared for tensile mechanical investigations by solvent casting (from methylene chloride solutions) into Teflon molds, followed by solvent evaporation at ambient temperature for 48 h, and then by heating at 140 °C, under high vacuum, for 24 h. The resulting materials were processed by compression molding above the glass transition temperature (for 10 min and 4 t of pressure) to obtain homogeneous films. GPC analyses of the polymers before and after processing showed identical molar mass and dispersity values, indicating that they remained stable under these conditions (Figures S52–S54). From the films, dumbbell-shaped specimens were cut, according to ISO 527-2 type 5B, and subjected to unilateral extension experiments, conducted according to ISO 527 (Table 3, Figures 7 and S60a). All the polycarbonates are strong plastics with variable tensile strengths, Youngs moduli, and toughness values. High molar mass PCPC-114 is both the strongest and most ductile, with a tensile strength reaching 58.5 ± 1.7 MPa and strain at break of 7.1 ± 1.9%. The PvCHC shows a tensile strength of 52.2 MPa and strain at break ∼5%. PCHC is both the weakest and most brittle, with an average tensile strength of 40.0 MPa and a strain at break of ∼3%; it also has the highest Young’s modulus of 2.2 GPa. The sample containing the ethyl substituent PeCHC shows better properties than the other two six-membered ring samples, with 5× greater elongation at break (18.7 ± 4.2%) and tensile toughness (46.7 ± 1.3 MPa) compared with PCHC (Table 3, entry 4).

**Table 3:** Mechanical Properties of Selected High Molar Mass Polycarbonates

polymer	σ (MPa)^a^	ε_b_ (%)^b^	*E*_*y*_ (GPa)^c^	*u*_t_ (MJ·m^–3^)^d^
PvCHC-125	52.2 ± 1.0	4.9 ± 1.3	1.33 ± 0.11	1.6 ± 0.6
PCHC-122	40.0 ± 1.8	3.3 ± 0.3	2.16 ± 0.06	0.9 ± 0.1
PCPC-114	58.5 ± 1.7	7.1 ± 1.9	1.70 ± 0.09	2.9 ± 1.1
PeCHC-125	46.7 ± 1.3	18.7 ± 4.2	1.31 ± 0.05	7.0 ± 1.5

a Ultimate tensile strength.

b Strain at break.

c Young’s modulus.

d Tensile toughness. Mean values ± std. dev. from measurements conducted independently on at least 4 specimens (Figures S55–S58 for full data).

### Polycarbonate Recycling

All new polymers should be investigated for efficient end-of-life recycling by both mechanical reprocessing and chemical recycling to monomers. Since these thermoplastics are all amorphous, they should be amenable to reprocessing at temperatures above the *T*_g_. The potential for mechanical recycling was explored by compression molding samples that had been subjected to mechanical testing. Samples of both PCHC-122 and PCPC-114 were reprocessed into homogeneous films and their mechanical properties were tested again (Figure S60b). Both samples showed tensile mechanical profiles equivalent to those of the original samples, indicating the potential for future mechanical recycling of the polymers (Figure S61).

**Figure 8:**
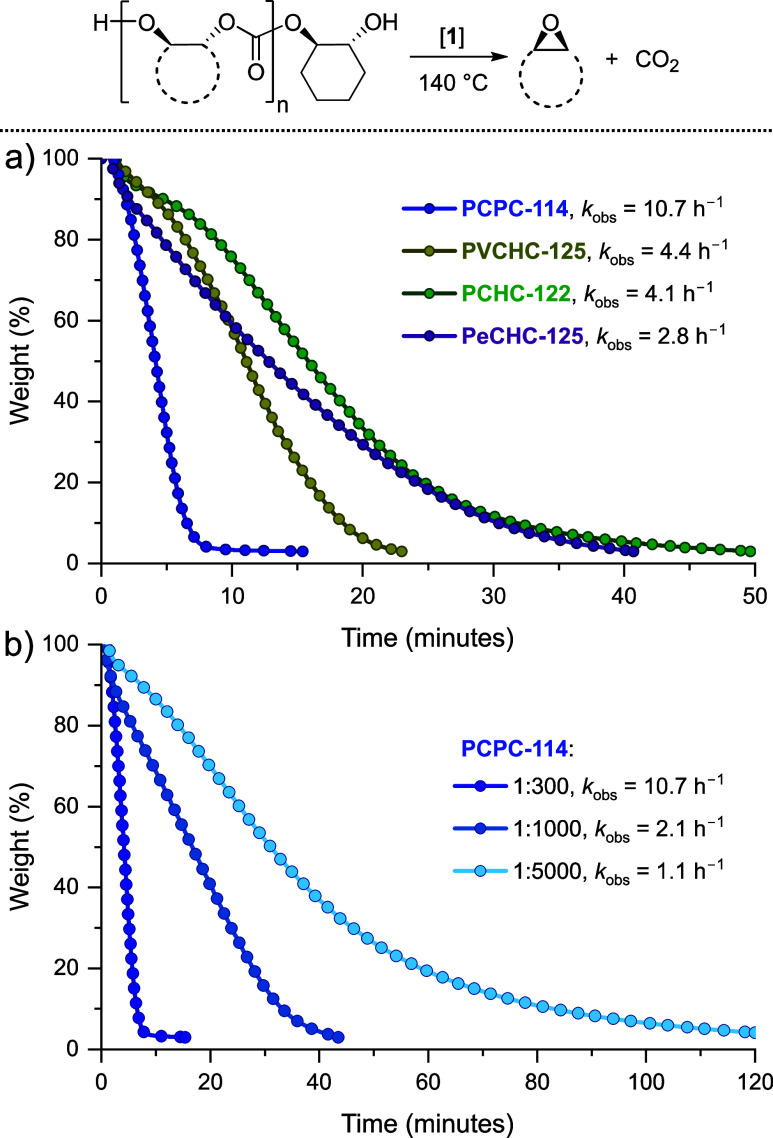
Isothermal TGA data at 140 °C comparing the chemical recycling of the polycarbonates in the presence of catalyst **1**: (a) constant [**1**]:[polycarbonate] (repeat unit) ratio of 1:300. (b) PCPC depolymerization with variable [**1**]:[PCPC] (repeat unit) ratio (Supporting Information for details). The data were fit using exponential functions, which allow for determination of recycling rates.

Next, the potential for polycarbonate chemical recycling to the constituent epoxides and carbon dioxide was explored in the solid state with catalyst **1**. Recently, we reported an efficient method for the chemical recycling of neat PCHC films, containing a dispersed catalyst, and investigated depolymerization rates using polymer weight loss vs time at constant temperature by thermogravimetric analysis.^68^ Following the same successful film recycling protocol, the polycarbonates were mixed with **1** in the solvent (THF) and cast as films into the TGA crucibles, followed by the complete removal of the solvent (Supporting Information for details). The polymer films were chemically recycled by heating to 140 °C, at a N_2_ flow rate of 25 mL min^–1^, and with continual monitoring of sample weight loss vs time. Using catalyst **1**, at loadings of 1:300, all of the polymers were efficiently depolymerized and formed only the starting *cis*-epoxide and CO_2_ as determined by in situ FTIR measurements (Figure 8a, Table S6, Figures S63–S66). It is noteworthy that even though the polymers are atactic, in all cases, depolymerization conversion and selectivity into *cis*-epoxide is high; absolute stereochemistry does not affect depolymerization, only relative *trans*-stereochemistry is important for chain-end backbiting to occur (Figure S67).

In all cases, the recycling occurred quickly, with typical TOF values of 700–1400 h^–1^. The chemical recycling rates were fastest for the PCPC and decreased in the order PCPC > PvCHC > PeCHC ∼ PCHC. The rate differences are proposed to arise from different stabilities for the alkoxide intermediates and/or from different transition state barriers to epoxide extrusion.^63^ Given the fast rates and promising thermal/mechanical properties of PCPC, it was selected for further insights into depolymerization catalysis. As such, progressively lower loadings of the catalyst were explored for the depolymerization reactions (Figure 8b). All depolymerizations were successful, yielding only epoxide (CPO) and CO_2_ and could be achieved at catalyst loadings of just 0.02 mol % (1:5000) and with a high TOF value of 2500 h^–1^ (Table S6). Finally, a larger-scale chemical recycling was conducted using ∼2 g of PCPC ([**1**]:[PCPC] 1:1000) in glassware (see the Supporting Information). After 1 h, at 140 °C, the epoxide was isolated in 72% yield and high purity, as indicated by ^1^H NMR spectroscopy (Figures S68). The depolymerization mechanism was studied for catalyst **2** in our prior work, where we proposed that depolymerization occurs via a chain-end mechanism. It is postulated that catalyst **1** operates by the same mechanism: when acetyl-end-capped PCHC (PCHC-OAc, Figure S69 for end-group analyses) was subjected to depolymerization conditions, no reaction occurred (Figure S70). With PCHC-OAc, hydrolysis of the organometallic catalyst **1** to form the alkoxide intermediate does not occur, which is a key step in chain-end depolymerization, and hence why no reaction was observed.

It is worth noting that a thermally cross-linked sample of PvCHC also underwent chemical depolymerization to vCHO under isothermal depolymerization at 140 °C in the TGA furnace (see the Supporting Information and Figures S71 and S72) and in the bulk. The low volatility under these conditions of the putative bis-epoxide cross-linker is expected since it was not detected by ^1^H NMR in the isolated monomer fraction (Figure S73). The 94% weight loss indicated a lightly cross-linked sample, i.e., only containing 6% weight of thermosetting units which, notably, did not impede its efficient depolymerization.

## Discussion

This work has established a successful new organometallic catalyst to produce a series of CO_2_-based polycarbonates, evaluated their properties, and recommended upon conditions for future reprocessing and depolymerization as a means of manufacture. Moreover, the catalysis advances described herein yield polycarbonates with sufficiently high molar mass values to more accurately determine and compare *M*_e_ values. The data are also fully consistent with the difficulties in processing and testing brittle PCHC: almost all prior investigations used samples that were below the critical molar mass or chain entanglement thresholds.^43,45,46,54,56^ Certainly, the steep frequency dependence of the *G*′ data in the rubbery plateau region makes the evaluation of *M*_e_ still somewhat challenging, and more refined estimates from the value of ∼56 kg mol^–1^ might still be possible in the future. In contrast, the much less widely investigated PCPC shows an order of magnitude lower *M*_e_, with entanglement being of the same magnitude as other strong thermoplastics, e.g., BPA-PC *M*_e_ = 1.6–4.8 kg mol^–1^, and is considerably lower than brittle thermoplastics, e.g., PLLA, *M*_e_ = 8–10 kg mol^–1^;^82^ PS, *M*_e_ = 13–18 kg mol^–1^.^83^ These data all suggest that future applications requiring high tensile strength and toughness should prioritize the five-membered cyclopentene or the ethyl-substituted cyclohexene ring substituents since the optimal thermal-mechanical properties will be accessed at substantially lower molar masses than the six-membered ring analogue.

Our mechanical data on the series of polycarbonates show that the most promising samples, PCPC and PeCHC, show favorable properties, with some in the range of existing commercial thermoplastics (Table S5). For example, PCPC has similar tensile mechanical properties to PLLA but is fully amorphous and shows a significantly higher glass transition temperature than PLLA, which would extend its upper use temperature (PCPC *T*_g_ = 85 °C vs PLLA *T*_g_ = 50–60 °C, respectively). Further, PCPC can undergo lower energy mechanical recycling than PLLA since the melting temperature for PLLA is typically 160–180 °C, whereas PCPC was effectively mechanically recycled, by compression molding, at 100 °C and displays a η_0_ of 0.78 MPa s.^84^ It is important to emphasize that, as an added benefit, the high carbonate linkage content in PCPC delivers 34 wt % carbon dioxide utilization (vs 30 wt % for PCHC). To maximize carbon dioxide utilization, the epoxide could also be sourced from biomass; there are already efficient routes to cyclopentene from lignocellulosic biomass since it is proposed for use in biobased aviation fuels.^85^ The synthesis involves bioderived furfuraldehyde being hydrogenated, over copper catalysts, to cyclopentanol in 60–90% yield and subsequent alcohol dehydration to cyclopentene, achieved in 84% yield using a recyclable solid acid catalyst.^85,86^

Our investigation also rationalizes the lack of development of PCHC as a stand-alone material—very few catalysts have ever accessed the optimal molar masses for better properties and in any case the high zero shear viscosity (90 MPa s) and moderate tensile mechanical properties suggest it would be best applied within copolymer structures in future.^38,47,48,54^ In contrast, PeCHC, featuring an ethyl substituent on the cyclohexene ring, combines high tensile strength (47 MPa), reasonable ductility (19% elongation at break), high glass transition temperatures (105 °C), low entanglement molar mass (13–18 kg mol^–1^), and low zero shear viscosity (1.26 MPa s). It is recommended for further development as a thermoplastic and may inspire the investigation of other substituted cyclohexane rings. Given that using bioderived epoxides may also help increase carbon dioxide uptake, terpenes such as limonene or menthene also feature substituted cyclohexane rings and their epoxides have precedent in this catalysis.^67,87−91^ Limonene oxide is challenging to polymerize due to its tertiary structure,^89,90^ but menth-2-ene oxide is an important future target given the influence in this work of ethyl substituents.^92^ Greiner and Agarwal reported a route to prepare moderate molar mass poly(meth-2-ene carbonate) (*M*_n_ = 20–30 kg mol^–1^), and the polymer showed a higher glass transition temperature (*T*_g_ = 144 °C) and higher temperature stability (*T*_d_ = 308 °C) than PCHC.^92^ In the future, the organometallic Mg(II)Co(II) catalyst could be used to target molar mass of poly(menth-2-ene carbonate) and determine its entanglement molar mass, tensile mechanical properties, and zero shear viscosity.

In the chemical recycling of polycarbonates to epoxides and carbon dioxide, complex **1** combines high rates, outstanding selectivity, and low loading tolerance and operates effectively using high molar mass polycarbonate—it is fastest using PCPC. Compared with other efficient PCPC depolymerization catalysts and processes its performance is excellent (Table S6). For example, catalyst **1** compares favorably against one of the best catalysts yet reported for PCPC recycling (Figure S62 for catalyst structures).^48^ The ammonium diborane catalyst is highly effective but requires the addition of KOH for activity. Even so, catalyst **1** enables depolymerization at 100× lower loading and shows ∼2000× higher activity. **1** is also more active than [Cr(salen)(Cl)]/PPN(N_3_) catalyst system which operates in neat polymer, showing efficient depolymerization at 60 °C lower temperature, using 10× lower loading and at ∼4× higher activity.^65^ Complex **1** also surpasses the original [Cr(salen)(Cl)]/^*n*^Bu_4_NN_3_ catalyst, used in solution in Darensbourg and co-workers’ seminal work, achieving activity values that are ∼2500× higher and operating at 1/100 loading.^61^ These data also support future focus on PCPC since it shows the most efficient chemical recycling catalysis and allowed, in laboratory scale experiments, for efficient closed loop recycling to cyclopentene oxide and CO_2_.

## Conclusions

A new organometallic Mg(II)Co(II) catalyst, applied with a diol, showed outstanding productivity and initiation control in the ROCOP of CO_2_ and cyclic epoxides. It was used to make a series of high molar mass polycarbonates, with *M*_n_ from 100 to 163 kg mol^–1^, by copolymerization of CO_2_ with vCHO, CHO, or CPO. The physical properties of these carbon dioxide-thermoplastics, together with an ethyl-substituted eCHO derived by hydrogenation of vinyl-polycarbonate, were investigated. The thermal, mechanical, and rheological experiments all demonstrate the significant influence of the ring size and alkyl-substituents over properties. The most widely investigated material in the literature, PCHC, showed a high temperature glass transition but suffered with low tensile strength and brittleness due to a lack of chain entanglements. Its entanglement molecular weight, *M*_e_, was very high (56 kg mol^–1^), which complicates future processing and applications. Achieving optimal properties will require ultrahigh molar mass samples (>10^3^ kg mol^–1^),^93−96^ with concomitant processing limitations due to its high viscosity. As such, perhaps it will be better to limit the use of PCHC as a stand-alone material and investigate its beneficial properties within copolymer structures. On the other hand, exactly the opposite conclusion is made regarding PCPC, which shows significant potential as a future thermoplastic. A PCPC with *M*_n_ = 114 kg mol^–1^ showed high tensile strength (59 MPa), high Young’s modulus (1.7 GPa), moderate elongation at break (7%), high tensile toughness (3 MJ m^–3^), a medium/high glass transition temperature (*T*_g_ = 85 or 97 °C by DSC or rheology), and low zero shear viscosity (0.78 MPa s). These properties arise from its low *M*_e_ (∼4 kg mol^–1^) with the consequence that in the future, only moderate molar mass polymers should be required to achieve the best material properties. Its properties are similar to those of polystyrene, albeit with a lower *M*_e_, and it improves upon the high use temperature limitations of poly(l-lactide) (PLLA). All the polycarbonates, including PCPC, are fully reprocessable by compression molding (100–140 °C, 10 min), providing a future mechanical recycling route. Further, the new Mg(II)Co(II) catalyst efficiently depolymerized the high molar mass polycarbonates to epoxides and CO_2_, providing a future chemical recycling option. The lead material, PCPC, underwent the fastest depolymerizations with high activity (TOF = 2500 h^–1^) and low catalyst loadings (1:5000), exceeding performances by several orders of magnitude for current literature catalysts. In future, PCPC should be prioritized for further investigation both in catalysis, application development and recycling chemistry. The new organometallic Mg(II)Co(II) catalyst, with its high productivity, low loading, and efficiency in either CO_2_ polymerization or depolymerization, should be investigated using other monomers and polymerizations to diversify the range of CO_2_-based polymers.

## Supporting Information Available

Characterization data of complexes (NMR, X-ray crystallography, magnetic moment, CV); polymerization data (NMR, MALDI-ToF, GPC chromatograms, DSC, TGA, Rheology, Tensile Testing); chemical recycling data (NMR, TGA-FTIR); and CheckCIF/PLATON report (PDF)
