# Supplementary material for: High Molar Mass Polycarbonates as Closed-Loop Recyclable Thermoplastics
Source: J Am Chem Soc. 2024 Mar 14;146(12):8381–93. doi: 10.1021/jacs.3c14170 (PMC10979403; doi:10.1021/jacs.3c14170)
Supplement: Supplementary file 1 — ja3c14170_si_001.pdf [file ja3c14170_si_001.pdf]

Supporting Information

High Molar Mass Polycarbonates as Closed-Loop Recyclable Thermoplastics

Gloria Rosetto,[a,#] Fernando Vidal,[a,‡] Thomas M. McGuire,[a] Ryan W. F. Kerr,[a] and Charlotte K. Williams[a*]

[a]Department of Chemistry, Chemistry Research Laboratory, University of Oxford, 12 Mansfield Rd, Oxford, OX1 3TA, U.K.

Current Address: [#]Renewable Resources and Enabling Sciences Center, National Renewable Energy Laboratory, Golden, CO 80401, USA. [‡]POLYMAT, University of the Basque Country UPV/EHU, Joxe Mari Korta Center, Avda. Tolosa 72, 20018 Donostia-San Sebastian, Spain.

Contents

Materials.....5

Methods.....5

Experimental Details.....7

Synthesis and characterization discussion for complex 1.....7

Synthesis of complex 3.....8

PvCHC hydrogenation to PeCHC.....8

Polymerizations at 1 bar of CO_2.....8

Polymerizations at 20 bar of CO_2.....9

TGA depolymerization experiments.....9

Synthesis of acetyl-end capped PCHC (PCHC-OAc).....9

Supplementary Figures and Tables.....10

Table S1: Crystallography data for 1 and 3......10

Table S2: Selected bond length and angles for complex 1......11

Figure S1: Molecular structure of complex 1 determined by X-ray diffraction using single crystals. ...11

Figure S2: ^{1}H NMR spectrum (toluene-d_8, 400 MHz) of complex 1......12

Figure S3: ^{13}C{^{1}H} NMR spectrum (toluene-d_8, 151 MHz) of 1......12

Figure S4: ^{19}F{^{1}H} NMR spectrum (toluene-d_8, 377 MHz) of complex 1......13

Figure S5: ^{19}F{^{1}H} COSY NMR spectrum (toluene-d_8, 377 MHz) of 1......14

Figure S6: SQUID plots of complex 1......14

Figure S7: Cyclic voltammetry curve of 1 in THF......14

Figure S8: ^{1}H NMR spectrum (toluene-d_8, 400 MHz) of [LCoCo(C_6F_5)$_2$] (3)......15

Figure S9: ^{19}F{^{1}H} NMR spectrum (toluene-d_8, 377 MHz) of 3......15

Figure S10: ^{13}C{^{1}H} NMR spectrum (toluene-d_8, 151 MHz) of 3......16

Figure S11: Molecular structure of 3......16

Table S3: Selected bond length and angles for complex 3......16

Figure S12: Stacked plot of ^{19}F{^{1}H} NMR spectra (toluene-d_8, 377 MHz) of 1 (bottom), 4-fluorophenol (middle), and the reaction between them (top)......17

Figure S13: Solution of complex 1 in toluene-d_8 before (left) and after (right) addition of 2 equivalents of 4-fluorophenol. Colour change is instantaneous. 18

Figure S14: Annotated MALDI-ToF spectra and corresponding m/z vs. repeat unit plots 19

Figure S15: Quantitative ^{13}C NMR spectrum ($CDCl_3$, 151 MHz) of isolated PCHC catalysed by 1. The polymer had a P_m value of 0.5 using the method reported by Coates *et al.*[13] 20

Figure S16: ^1H NMR spectrum ($CDCl_3$, 400 MHz) of polymerization aliquot. 20

Figure S17: Initial rates comparison between catalyst 1 and 2. 21

Figure S18: Illustration of epoxide/carbon dioxide ROCOP initiation, propagation for catalyst bearing the organometallic ligand, C_6F_5, and chain transfer reactions with alcohols. 22

Figure S19: GPC analysis of PvCHC-163. 23

Figure S20: GPC analysis of PCHC-122. 23

Figure S21: GPC analysis of PCPC-110. 24

Figure S22: ^1H NMR spectrum ($CDCl_3$, 400 MHz) of PeCHC. 24

Figure S23: GPC analysis of PvCHC and PeCHC. 25

Figure S24: ^1H NMR spectrum ($CDCl_3$, 400 MHz) of an aliquot corresponding to Table 1, entry 5. 25

Figure S25: ^1H NMR spectrum ($CDCl_3$, 400 MHz) of an aliquot corresponding to Table 1, entry 7. 26

Figure S26: ^1H NMR spectrum ($CDCl_3$, 400 MHz) of an aliquot corresponding to Table 1, entry 8. 27

Figure S27. Overlay of DSC data (a) and TGA data (b) 28

for PCPC-114 (blue), PeCHC-125 (purple), PvCHC-125 (dark yellow), and PCHC-122 (green). 28

Figure S28: DSC data for PvCHC with increasing M_n. 29

Figure S29: Overlay of TGA data for PvCHC series 29

Rheology Measurements and Time-Temperature Superposition (TTS). 30

Figure S30: Representative TTS master curve of PCPC-114 31

Table S4: Viscoelastic parameters obtained from the time-temperature superposition mastercurves (T_{ref} = 140 °C). 32

Figure S31 Oscillatory temperature ramp of PCPC-114 (0.5% strain, 1.0 Hz, 2 °C · min^{-1}) between 75 and 190 °C. 33

Figure S32: Mastercurves of PCPC-114 constructed by time-temperature superposition (TTS) referenced to 140 °C (0.5% strain, 0.01–100 Hz) 34

Figure S33: Oscillatory temperature ramp of PeCHC-125 (0.5% strain, 1.0 Hz, 2 °C · min^{-1}) between 90 and 200 °C 35

Figure S34: Mastercurves of PeCHC-125 constructed by time-temperature superposition (TTS) referenced to 140 °C (0.5% strain, 0.01–100 Hz). 36

Figure S35: Oscillatory temperature ramp of PvCHC-125 containing 0.1 wt% of a thermal stabilizer (0.5% strain, 1.0 Hz, 2 °C · min^{-1}) between 100 and 220 °C. 37

Figure S36: Mastercurves of PvCHC-125 containing 0.1 wt% of a thermal stabilizer constructed by time-temperature superposition (TTS) referenced to 140 °C (0.5% strain, 0.01–100 Hz). 38

Figure S37: Oscillatory temperature ramp of PCHC-122 (0.5% strain, 1.0 Hz, 2 °C · min^{-1}) between 100 and 220 °C 39

Figure S38: Mastercurves of PCHC-122 constructed by time-temperature superposition (TTS) referenced to 140 °C (0.5% strain, 0.01–100 Hz). 40

Figure S39: Oscillatory frequency sweeps (0.5% strain) of PCPC-114 employed in the creation of mastercurves. 41

Figure S40: Temperature dependence of the shift factors, α_T, employed for the construction of the mastercurve of PCPC-114 (black diamonds) referenced at 140 °C and fitted curve (red line) to the WLF equation. 41

Figure S41: Oscillatory frequency sweeps (0.5% strain) of PeCHC-125 employed in the creation of mastercurves. 42

Figure S42: Temperature dependence of the shift factors, α_T, employed for the construction of the mastercurve of PeCHC-125 (black diamonds) referenced at 140 °C and fitted curve (red line) to the WLF equation. 42

Figure S43: Oscillatory frequency sweeps (0.5% strain) of PvCHC-125 containing 0.1 wt% of a thermal stabilizer employed in the creation of mastercurves. 43

Figure S44: Temperature dependence of the shift factors, α_T, employed for the construction of the mastercurve of PvCHC-125 containing 0.1 wt% of a thermal stabilizer (black diamonds) referenced at 140 °C and fitted curve (red line) to the WLF equation. 43

Figure S45: Oscillatory frequency sweeps (0.5% strain) of PCHC-122 employed in the creation of mastercurves. 44

Figure S46: Temperature dependence of the shift factors, α_T, employed for the construction of the mastercurve of PCHC-122 (black diamonds) referenced at 140 °C and fitted curve (red line) to the WLF equation. 44

Figure S47: Comparison of thermal scans by DSC of PvCHC under air (10 °C min^{-1}): unstabilized sample (purple line) and stabilized sample (yellow line) by addition of 0.1 wt% of a radical inhibitor pentaerythritol tetrakis(3,5-di-tert-butyl-4-hydroxyhydrocinnamate) 46

Figure S48: Oscillatory temperature ramp of unstabilized PvCHC-125 (0.5% strain, 1.0 Hz, 2 °C · min^{-1}) between 100 and 220 °C. 47

Figure S49: Normalized stress relaxation curves of unstabilized PvCHC-125 at various increasing temperatures. 48

Figure S50: Normalized stress relaxation curves of PCPC-114 at various increasing temperatures. 48

Figure S51: Diagram showing the cross-linking of a unstabilized PvCHC film sample. 49

PvCHC-125 treated at 180 °C for 2 hours would no longer dissolve in dioxane, instead forming a swollen gel which is also typical of some chain crosslinking 49

Figure S52: GPC analysis of PCHC-122 before and after hot-pressing. 49

Figure S53: GPC analysis if PCPC-114 before and after hot pressing. 50

Figure S54: GPC analysis of PvCHC-125 before and after hot pressing (no stabilizer added). 50

Table S5: Thermal-mechanical properties of polymers in this work and commercial polymers. 51

Figure S55: Tensile data for PvCHC-125 (without stabilizer). 51

Figure S56: Tensile data for PCHC-122. 52

Figure S57: Tensile data for PCPC-114. 52

Figure S58: Tensile data for PeCHC-125. 53

Figure S59: Tensile data for PvCHC-xlinked 53

Figure S60: a) Images of the polymer specimens before and after tensile testing. b) Samples of PCHC-122 and PCPC-114 re-processed into a homogeneous film by compression moulding for 10 min at 140 °C and 100°C, respectively. 54

Figure S61: Tensile graphs of PCHC (left) and PCPC (right) specimens after reprocessing. 54
Table S6: Depolymerization data for PCHC, PCPC and PvCHC using catalyst 1 and prior literature reports on the depolymerization of PCPC. 55
Figure S62. Structures of catalysts used for the depolymerization of PCPC.[18-20] 55
Figure S63. TGA-FTIR gas-phase analysis of PCHC-122 depolymerization mixture catalysed by 1. ...56
Figure S64. TGA-FTIR gas-phase analysis of PvCHC-125 depolymerization mixture catalysed by 1 ..56
Figure S65. TGA-FTIR gas-phase analysis of PCPC-114 depolymerization mixture catalysed by 1. 57
Figure S66. TGA-FTIR gas-phase analysis of PeCHC-125 depolymerization mixture catalysed by 1...57
Figure S67: Example depolymerization mechanism showing backbiting reactions with stereochemical inversion to form *cis*-epoxide, irrespective of absolute stereochemistry. 57
Figure S68: ^{1}H NMR spectrum ($CDCl_3$) of CPO isolated from the depolymerization of PCPC-114. 58
The reaction was conducted at 140 °C with $[cat]_0$: $[PCPC\text{-}114]_0$ = 1: 1000. 58
Figure S69: End group analysis of PCHC and PCHC-OAc by $^{31}P\{^1H\}$ NMR spectroscopy 58
Figure S70: Isothermal TGA data at 140 °C comparing the depolymerization of the PCHC-OAc and PCHC 59
Figure S71. Representative weight vs. time plots for the depolymerization of PvCHC-125 (yellow), PVCHC x-linked (pink) catalysed by 1. 59
Figure S72. TGA-FTIR gas-phase analysis of PvCHC x-linked depolymerization mixture catalysed by 1. 60
Figure S73: ^{1}H NMR spectrum ($CDCl_3$) of vCHO isolated from the depolymerization of PvCHC-x-linked. 60
References 61

Materials

Solvents used in air-sensitive synthesis were collected from a solvent purification system (SPS), degassed by three freeze-pump-thaw cycles, and stored under 3 Å molecular sieves, under an N_2 atmosphere. THF was additionally dried, over CaH_2, and distilled prior to use. All chemicals purchased, purified, or synthesised were stored under an inert atmosphere. $CoBr_2$ (Sigma Aldrich, 98 %) and bromo(pentafluoro)benzene (Fluorochem, 99 %) were used as received. $[Mg\{N(SiMe_3)_2\}_2]$ (Sigma Aldrich, 95 %) was recrystallised, from hexane, and dried, under vacuum, prior to use. 4-Fluoropehnol (Sigma Aldrich, 99 %) was sublimed, under high vacuum at room temperature, prior to use. Toluene-d_8 (Cambridge Isotope Laboratories Inc.) was dried, over CaH_2, degassed by three freeze-pump-thaw cycles, and dried under 3 Å molecular sieves, prior to use. LH_2 was synthesised according to a literature procedure, and was recrystallised, from methanol and dried under vacuum, prior to use.[1] $[Co(C_6F_5)_2(THF)_2]$ was synthesized according to a literature procedure,[2-3] and recrystallised twice from a 1:6 THF/hexane mixture, at −30 °C, prior to use. $[LMgCo(OAc)_2]$ (**2**) was prepared according to a literature procedure.[4] Cyclohexene oxide (CHO, Acros Organics, 98 %) was stirred over CaH_2 for three days, followed by fractional distillation, under an N_2 atmosphere, at 140 °C. It was subsequently distilled from ^{n}BuLi (2.5 M in hexanes), at 40 °C and 35 mbar, and then fractionally distilled, at 40 °C and 35 mbar. 4-Vinyl-cyclohexene oxide (vCHO, Sigma Aldrich, 98 %) was dried and distilled once, over CaH_2, at 50 °C, under 10 mbar. Cyclopentene oxide (CPO, Sigma Aldrich, 98 %) was stirred over CaH_2, for 3 days and distilled, under an N_2 atmosphere, at 120 °C. CO_2 gas (BOC, CP grade, 99.995 %) was passed through two carbon dioxide purifiers (VICI) at point of use. 1,2-Cyclohexanediol (Sigma Aldrich, 98 %) was recrystallized from ethyl acetate.

Methods

All manipulations, unless otherwise stated, were carried out under inert conditions, either in a N_2 filled MBraun glovebox or a dual manifold N_2-vacuum Schlenk line.

NMR Spectroscopy: ^{1}H, COSY, ^{19}F{^{1}H} NMR spectra were obtained using a Bruker AVIII HD 400 NMR spectrometer. ^{13}C{^{1}H} NMR spectra were obtained using a Bruker AV III 500 spectrometer, equipped with a cryoprobe. All spectra were recorded at 298 K, unless stated otherwise.

Cyclic Voltammetry: Electrochemical studies were carried out using a PalmSens EmStat Blue potentiostat. Cyclic voltammetry experiments were performed in N_2 glovebox, using a three-electrode configuration: with an Au disc (2.0 mm^2) as the working electrode, a Pt wire as the counter electrode and a Ag wire as the pseudo-reference electrode. Sample solutions were prepared by dissolving the analyte (*ca.* 5 mM), in THF (10 mL), followed by the addition of the electrolyte $[^{n}Bu4N][PF_6]$. The reported mid-peak potential was recorded at a scan rate of 0.1 V s^{-1} and was referenced against the $Fe(Cp)_2^+/Fe(Cp)_2$ redox couple, which was measured by adding ferrocene (*ca.* 1 mg) to the sample solution.

SQUID Magnetometry: SQUID measurements were carried out on powdered samples using a Quantum Design MPMS-3 Magnetometer, with a field of 0.1 T, at temperatures from 2 to 300 K. The sample (*ca.* 15 mg) was placed into a gelatine capsule and then loaded into a diamagnetic plastic straw under inert atmosphere, prior to placing it in the cryostat. Magnetic susceptibility values were corrected using the diamagnetic Pascal constants.

X-ray Crystallography: Air sensitive single crystals were isolated in the glovebox and immersed in fluorinated oil before analysis. Crystalline samples were mounted on a MiTeGen Micromount and cooled to 150 K, with dry nitrogen flow, using an Oxford Cryostream.[5] Data was collected with an Oxford Diffraction Supernova diffractometer using Cu Kα (λ = 1.5417 Å) radiation. The resulting reflection data was processed with CrysAlis Pro.[6-8] Structures were solved using the SHELXT program and least-square refined using the SHELXL program within the Olex2 system suite.[7-9]

Gel Permeation Chromatography (GPC): GPC analysis was carried out on a Shimadzu LC-20AD instrument, equipped with a Refractive Index (RI) detector and two PSS SDV 5 µm linear M columns, with an eluent of HPLC-grade THF, at 30 °C and at a flow rate of 1.0 mL min^{-1}.

Matrix-Assisted Laser Desorption/Ionization Time of Flight (MALDI-ToF): MALDI-ToF was carried out on a Bruker Autoflex Speed instrument. A 1:1:4 solution of polymer (20 mg mL^{-1} in THF), potassium trifluoroacetate (20 mg mL^{-1} in THF), and dithranol (20 mg mL^{-1} in THF) was prepared and spotted twice on a MALDI stainless steel plate. The samples were allowed to dry completely before being subjected to analysis.

Differential Scanning Calorimetry (DSC): DSC measurements were made using a DSC 25 (TA Instruments). Samples were heated to 200 °C for 5 minutes, to remove thermal history, before heating and cooling, from 30 °C to 200 °C, at a rate of 20 °C min^{-1}. Each sample was run for three heating and cooling cycles. Glass transition temperatures (T_g) were determined from the midpoint of the transition during the second heating curve.

Thermogravimetric Analysis (TGA): TGA measurements were made on a Discovery TGA 5500 (TA Instruments), heating at 10 ˚C min^{-1}, between 30–700 ˚C. Polymer samples were purified by column chromatography and dried in a vacuum oven above the polymer's T_g prior to use.

TGA-FTIR: Measurements were collected on a Nicolet iS20 FTIR spectrometer (Thermo Scientific Instruments) coupled to a TGA5500 (TA instruments). The mass flow of the TGA was set to 30 mL min^{-1}. The FTIR spectrometer was equipped with a KBr/Ge beamsplitter, fast-recovery deuterated triglycine sulfate KBr detector and solid-state diode laser. Spectra were recorded, between 400–4000 cm^{-1}, with 10 scans per spectrum, at a resolution of 8 cm^{-1}.

Tensile Testing: The polymer was solvent cast from methylene chloride solutions into a rectangular-shaped PTFE mould followed by solvent evaporation, at ambient temperature for 48 h, and then by heating at 140 ˚C, under high vacuum, for 24 h. The resulting materials were processed by compression molding above the glass transition temperature (for 10 mins and 4 metric tonnes of pressure). The resulting homogeneous films were cut into dumbbell-shaped specimens, using a Zwick ZCP020 cutting press equipped with a cutting die for ISO 527-2 type 5B. Uniaxial extension experiments were run according to ISO 527 using a Universal Testing Instrument (Instron) at an extension rate of 10 mm min^{-1}.

Thermal Press: Polymer films were processed or reprocessed using the Carver mini CH CE Press (5420CE.4010C00) with heated plates and a hydraulic press.

Calculating $M_{n, est}$ for PvCHC

From the M_n data obtained from the loading studies of PvCHC (Table 1), the equivalents of residual diols (CTA) present in the vCHO and CO_2 used in the polymerizations can be estimated (it is difficult to decouple their contributions). The procedure was adapted from Diment and Williams.[10] The

calculated amount is then subsequently applied to $M_{n,\,est}$ in Table 1. The same treatment cannot be done for CHO and CPO as a single data point may not be representative. This value is determined as follows:

Example case Table 1 entry 4 (where $M_{n,\,total}$ = (epoxide conv.)(epoxide equiv.)(MW repeat unit):

$$\frac{M_{n,\,total}}{M_{n,\ GPC}} = [CTA]_{added} + [CTA]_{residual}$$

$$\frac{499500}{81900} = 6.09 = 4 + [CTA]_{residual}$$

$$2 \approx [CTA]_{residual}$$

Roughly, there are 2 equivalents of residual CTAs for every 3000 equivalents of vCHO used. This quantity can be applied to other entries in Table 1, obtaining $M_{n,est}$ values close to what is determined experimentally ($M_{n\,,\,GPC}$).

Experimental Details

Synthesis and characterization discussion for complex 1

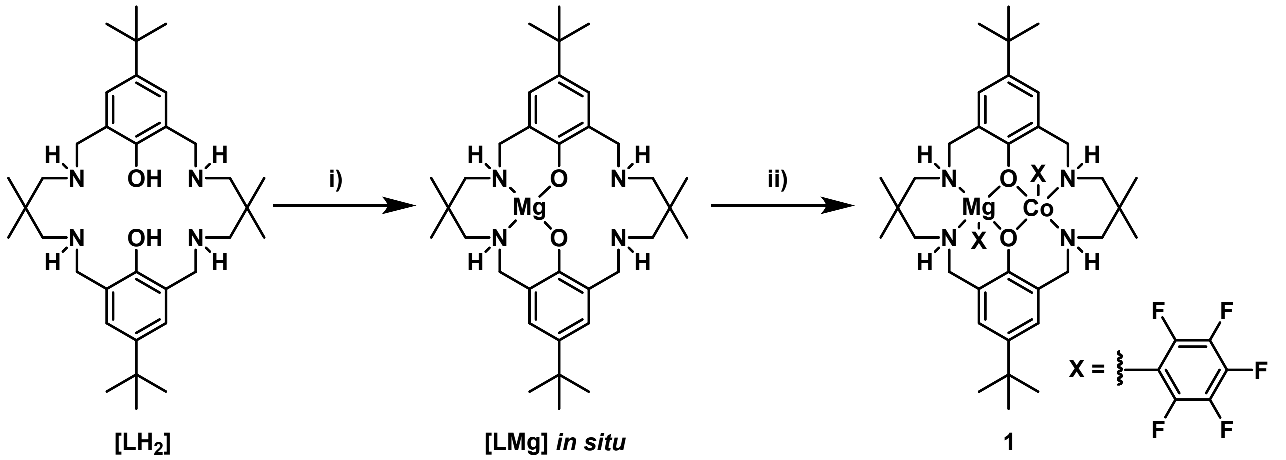

[LH$_2$] [LMg] *in situ* 1

i) [Mg{N(SiMe$_3$)$_2$}$_2$], THF, RT, 2 h
ii) [Co(C$_6$F$_5$)$_2$(THF)$_2$], THF, RT, 16 h

The macrocyclic proligand [LH$_2$] (150 mg, 0.27 mmol, 1 equiv.) and [Mg{N(SiMe$_3$)$_2$}$_2$] (94 mg, 0.27, 1 equiv.) were stirred in anhydrous THF (15 mL), in a sealed vial, for 2 h, in a glovebox. A concentrated solution of [Co(C$_6$F$_5$)$_2$(THF)$_2$] (145 mg, 0.27 mmol, 1 equiv.) was added, dropwise, and stirred overnight at room temperature, observing a colour change from aquamarine to dark brown/purple. The solution was cooled, to –30 ˚C for 24 h, to afford a purple crystalline solid. The solution was decanted, and the solid residue was washed with cold (–30 ˚C) THF and hexane, and dried, *in vacuo* overnight, at room temperature (0.14 mmol, 52 % yield). The product was stored at –30 ˚C, in a glovebox. Crystals suitable for X-ray diffraction were grown from a –30 ˚C solution in THF after standing for 48 h.

^{1}H NMR (toluene-d_8, 400 MHz): δ 264.51, 220.88, 148.51, 75.08, 29.42, 25.13, 10.29, 7.77, 6.34, 3.28, 2.58, -0.42, -1.98, -16.60, -56.33 ppm. ^{19}F{^{1}H} NMR (toluene-d_8, 377 MHz): δ -68.5 (s, 1 F), -153.6 (t, 2 H, J = 19.1 Hz, 2 F), -155.5 (br, 1 F) ppm. ^{13}C{^{1}H} (toluene-d_8, 151 MHz): δ 247.7, 205.1, 173.1, 172.3, 150.7, 141.2, 73.3, 51.6, 48.3, 47.3, 43.6, 42.5, 36.9, 29.8, 24.5, 12.4, 9.4, -12.7 ppm. Magnetic moment: μ_{eff} = 4.99 μ_B. Cyclic voltammetry: [MgCo^{II}]/[MgCo^{III}]$^+$ E_{pa} = −0.891 V.

Note: The $^{19}F\{^1H\}$ NMR spectrum (Figure S4) shows only 3 peaks for complex **1** in solution. It is hypothesized that only the *meta*- and *para*-fluorine substituents on the phenyl ring coordinated to Mg(II) and the *para*-fluorine substituents on the ring attached to Co(II) are observed – the $^{19}F\{^1H\}$ COSY (Figure S5) shows that the peaks at -155 and -154 ppm are coupling (*meta* and *para*-F on Mg(II)), and the peak at -65 ppm does not couple to anything (Co(II) *para*-F). This assignment is also consistent with their relative integration. The remaining aromatic F atoms are not observed due to significant broadening associated with their proximity to the paramagnetic Co(II) centre.

For comparison, the analogous Co(II)Co(II) complex (**3**) was prepared, by combining LH_2 with two equivalents of $[Co(C_6F_5)_2(THF)_2]$. By ^1H and ^{13}C NMR spectroscopy (Figures S8 and S10), the number of peaks suggests a symmetrical complex, and a single peak was observed in the $^{19}F\{^1H\}$ NMR spectrum at −71 ppm. This peak was consistent with only the *p*-fluorine on the C_6F_5 co-ligand being observed, exactly as has been assigned for complex **1** (Figure S9). The single crystal XRD measurements also result in a molecular structure that is a very similar to **1**, although evidently with two Co(II) centres (Figure S11 and Table S3). This data supports the speciation of complex **1** as a heterodinuclear rather than a mixture of two homonuclear complexes.

Synthesis of complex 3

$[Co(C_6F_5)_2(THF)_2]$ (39 mg, 0.073 mmol, 2 equiv.) was added to a cold solution of $[LH_2]$ (20 mg, 0.036 mmol, 1 equiv.) in THF (0.6 mL). After stirring for 5 minutes, the solution turned dark/deep purple. After 1 h, the solution was cooled to −30 °C and kept at this temperature for 24 h to afford a purple crystalline solid. Crystals suitable for X-ray diffraction were grown from the saturated complex solution (THF) at −30 °C.

^1H NMR (toluene-d_8, 400 MHz): δ 269.93, 229.57, 187.21, 94.15, 25.46, 5.92, -0.30, -27.11, -75.77 ppm. $^{19}F\{^1H\}$ NMR (toluene-d_8, 377 MHz): δ -71.2 ppm. $^{13}C\{^1H\}$ (toluene-d_8, 151 MHz): δ 275.7, 223.8, 222.0, 166.3, 108.7, 107.8, 75.7, 73.0, 69.3, -12.2 ppm.

PvCHC hydrogenation to PeCHC

Following a modified literature procedure, PvCHC (1.78 g, 1.0 equiv. of olefin) was added to 2-neck round-bottomed flask, in air.[11] Anhydrous *p*-xylene (30 mL) was added and the mixture left to stir until the polymer dissolved (*ca.* 10 minutes). Tri-n-butylamine (11.2 mL, 47.06 mmol, 4.44 equiv.) and *p*-toluenesulfonylhydrazide (8.91 g, 47.81 mmol, 4.51 equiv) were added to the flask. To prevent overpressure during the reaction, one arm of the reaction flask was left open to air. The reaction flask was heated to 140 °C to form a yellow solution which gradually darkened to brown over time. After 3 h, the reaction was cooled to room temperature. The solution was precipitated by adding it into methanol (~250 mL) and the solid collected. The solid was then dissolved in methylene chloride (~ 100 mL) and washed with water (x 3, ~100 mL). The organic phases were collected, dried over $Mg(SO_4)_2$ and filtered. The solvent was removed, *in-vacuo* at 40 °C, before further drying the polymer, in a vacuum oven at 60 °C, to yield PeCHC as a white solid (1.22 g, 68% yield).

Polymerizations at 1 bar of CO_2

For polymerizations run at 1 bar of CO_2, in a glove box the catalyst, vCHO, CHD, and toluene were combined in a Rotaflo ampoule, equipped with a rare-earth magnetic stirrer bar. The ampoule was cycled three times onto a triple manifold steel Schlenk line, with cycling occurring between vacuum

and CO_2, and the headspace was evacuated and refilled with CO_2 (three times) before placing the flask in a pre-heated (100 °C) oil bath.

Polymerizations at 20 bar of CO_2

For polymerizations run at 20 bar of CO_2, catalyst, in a glove box the epoxide, CHD, and toluene were combined in a 25 mL or 100 mL stainless steel Parr pressure vessel. The vessel was heated to 80 °C and pressurised using 20 bar of CO_2 once the temperature was reached. To stop the reaction, the vessel was cooled to room temperature and the remaining CO_2 pressure was vented. A sample of the crude polymer was collected for analysis by ^{1}H NMR spectroscopy. The viscous solution was diluted with methylene chloride and the polymer was precipitated by adding it into acidified methanol (HCl, this also quenches any unreacted epoxide). After drying, the polymer was re-diluted in methylene chloride and passed through a silica plug, to remove the catalyst, followed by its re-precipitation from methanol. Finally, the polymer was dried, in a vacuum oven at 60 °C for 24 h.

TGA depolymerization experiments

Depolymerization experiments were performed in sealed TZero aluminium pans, which were opened immediately prior to being loaded into the TGA furnace. A typical procedure for the depolymerization is given below:
In the glovebox, PCHC (142 mg, 1.00 mmol) was added to a vial and dissolved in THF (1 mL). Catalyst was added to the vial as a stock solution at the appropriate loading (0.01 M in THF). A portion of the catalyst:polymer solution (40 µL) was transferred to an aluminium Tzero TGA crucible. The crucible was placed under vacuum, for 30 minutes, before being crimped in the glovebox with a hermetic seal. The crucible was then transferred to a TGA instrument for monitored solid-state depolymerization. Rate coefficients were extracted by fitting the data to an exponential function and using data from 10-95% mass loss.

Synthesis of acetyl-end capped PCHC (PCHC-OAc)

Following an adapted literature procedure:[12] under an N_2 atmosphere, acetyl chloride (0.21 mL, 2.93 mmol, 0.7 equiv.) was added dropwise to a THF (5 mL) solution of α-ω hydroxy PCHC (0.594 mg, 4.18 mmol, 1 equiv.) and triethylamine (0.58 mL, 4.18 mmol, 1 equiv.) at 0 °C. After 5 minutes, a white precipitate formed. The reaction solution was allowed to warm to room temperature overnight before being precipitated from methanol. The solid phase was collected by centrifugation. The solid phase was then reprecipitated and centrifuged twice more, before being dried under vacuum to yield PCHC-OAc as an off-white powder (296 mg, 2.08 mmol, 50% yield).

Supplementary Figures and Tables

Table S1: Crystallography data for **1** and **3**.

**Complex**	**1**	**3**
**Local Code**	010rwfk21	009rwfk22
**Compound**	CoMg	CoCo
**CCDC number**	2208898	2208899
**Crystal data**		
**Chemical formula**	$C_{46}H_{54}CoF_{10}MgN_4O_2·4(C_4H_8O)$	$C_{23}H_{27}CoF_5N_2O·2(C_4H_8O)$
***M*r**	1256.58	645.60
**Crystal System, space group**	Triclinic, *P*¯1	Triclinic, *P*¯1
***a*, *b*, *c* (Å)**	10.7864 (7), 11.9681 (7), 13.1272 (7)	10.7361 (7), 11.8550 (9), 13.0799 (9)
**a, b, g (°)**	70.242 (5), 84.445 (5), 73.579 (5)	70.812 (6), 84.485 (5), 74.391 (6)
***V* (Å3)**	1529.81 (17)	1514.2 (2)
Z	1	2
**m (mm-1)**	3.02	5.01
**Crystal size (mm)**	0.53 × 0.06 × 0.05	0.10 × 0.08 × 0.07
**Data collection**		
**Absorption correction**	Multi-scan *CrysAlis PRO* 1.171.41.81a (Rigaku Oxford Diffraction, 2020) Empirical absorption correction using spherical harmonics, implemented in SCALE3 ABSPACK scaling algorithm.	Gaussian *CrysAlis PRO* 1.171.40.53 (Rigaku Oxford Diffraction, 2019) Numerical absorption correction based on gaussian integration over a multifaceted crystal model Empirical absorption correction using spherical harmonics, implemented in SCALE3 ABSPACK scaling algorithm.
***T*min, *T*max**	0.901, 1.000	0.745, 0.889
**No. of measured, independent and observed [*I* > 2s(*I*)] reflections**	20972, 6309, 4400	14974, 6242, 5039
***R*int**	0.074	0.047
**(sin q/l)max (Å-1)**	0.631	0.630
**Refinement**		
R*[*F*2 > 2s(*F*2)], *wR*(*F*2), *S	0.057, 0.163, 1.02	0.054, 0.147, 1.03
**No. of reflections**	6309	6242
**No. of parameters**	387	384
**Dñmax, Dñmin (e Å-3)**	0.57, -0.30	1.01, -0.40

Table S2: Selected bond length and angles for complex **1**.

Bond	Length (Å)	Bond	Angle (°)
Co(1)-O(1)	2.050(12)	O(1)-Co(1)-N(1A)	150.9(7)
Mg(1)-O(1)	2.010(2)	O(1A)-Co(1)-N(2A)	147.7(7)
Co(1)-C(35A)	2.182(14)	O(1A)-Mg(1)-N(1)	150.4(14)
Mg(1)-C(35)	2.160(3)	O(1)-Mg(1)-N(2)	146.8(14)

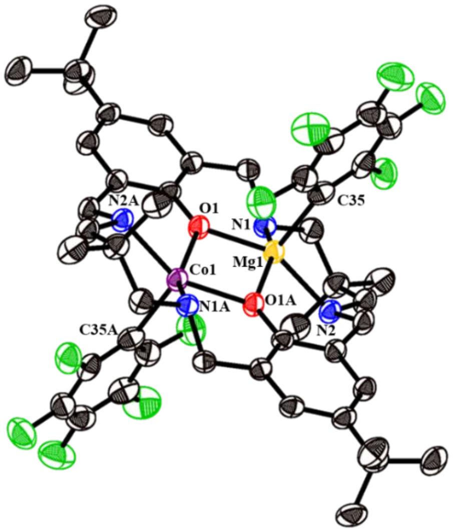

Figure S1: Molecular structure of complex **1** determined by X-ray diffraction using single crystals. H atoms and two THF molecules have been omitted for clarity. Thermal ellipsoids at 50 % probability level. Co = purple, Mg = yellow, O = red, N = blue, F = green, C = grey.

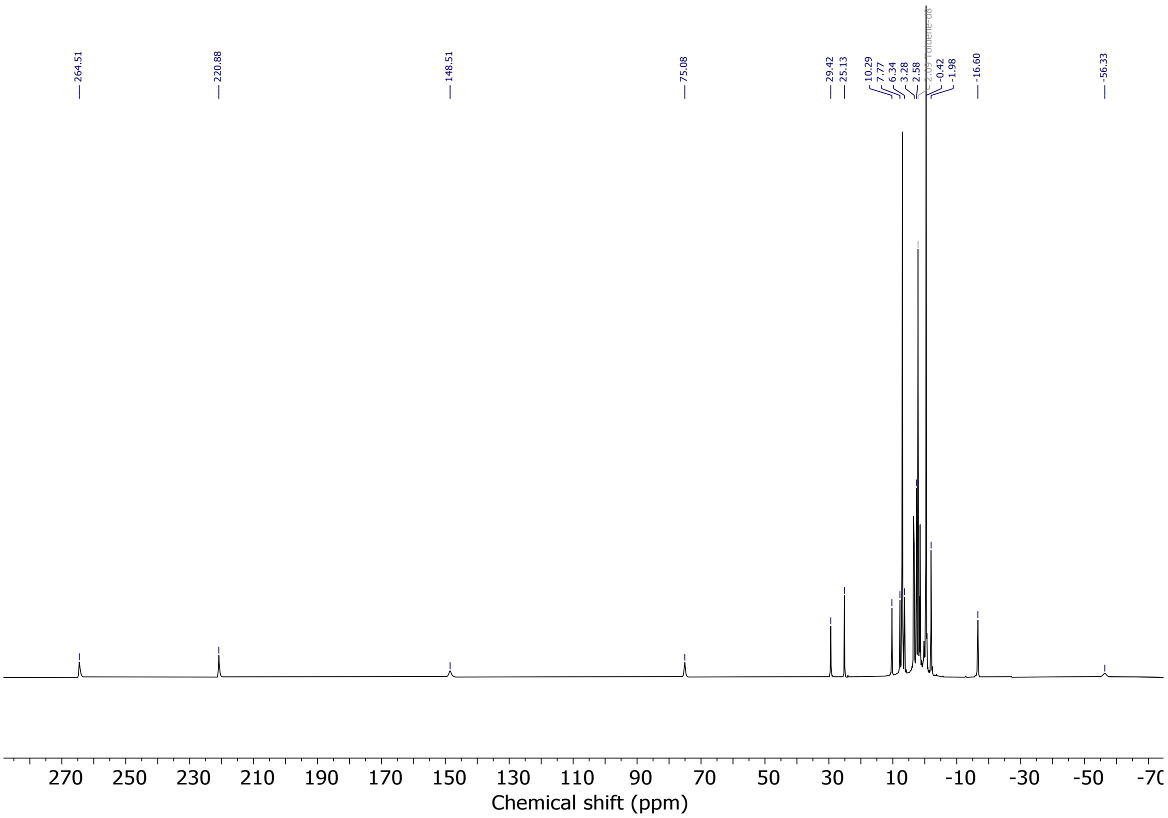

Figure S2: ^{1}H NMR spectrum (toluene-d_8, 400 MHz) of complex **1**.

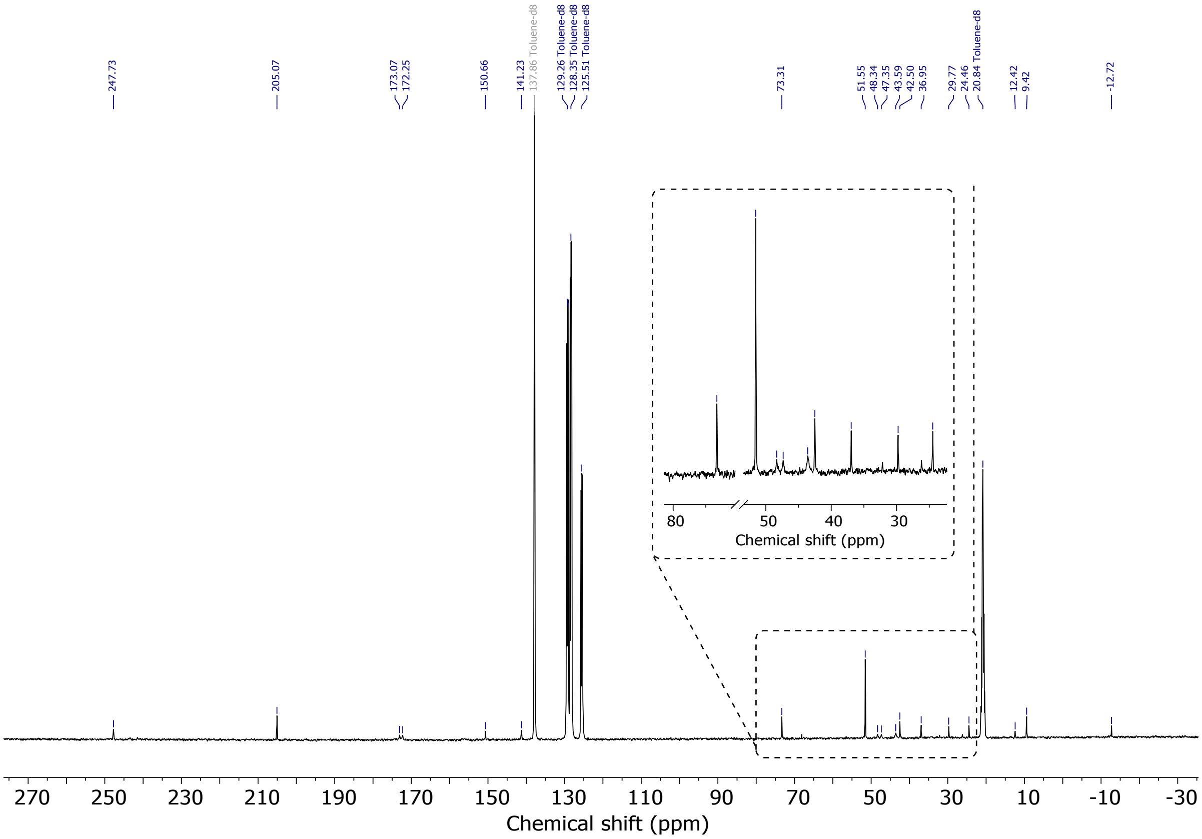

Figure S3: $^{13}C\{^{1}H\}$ NMR spectrum (toluene-d_8, 151 MHz) of **1**.

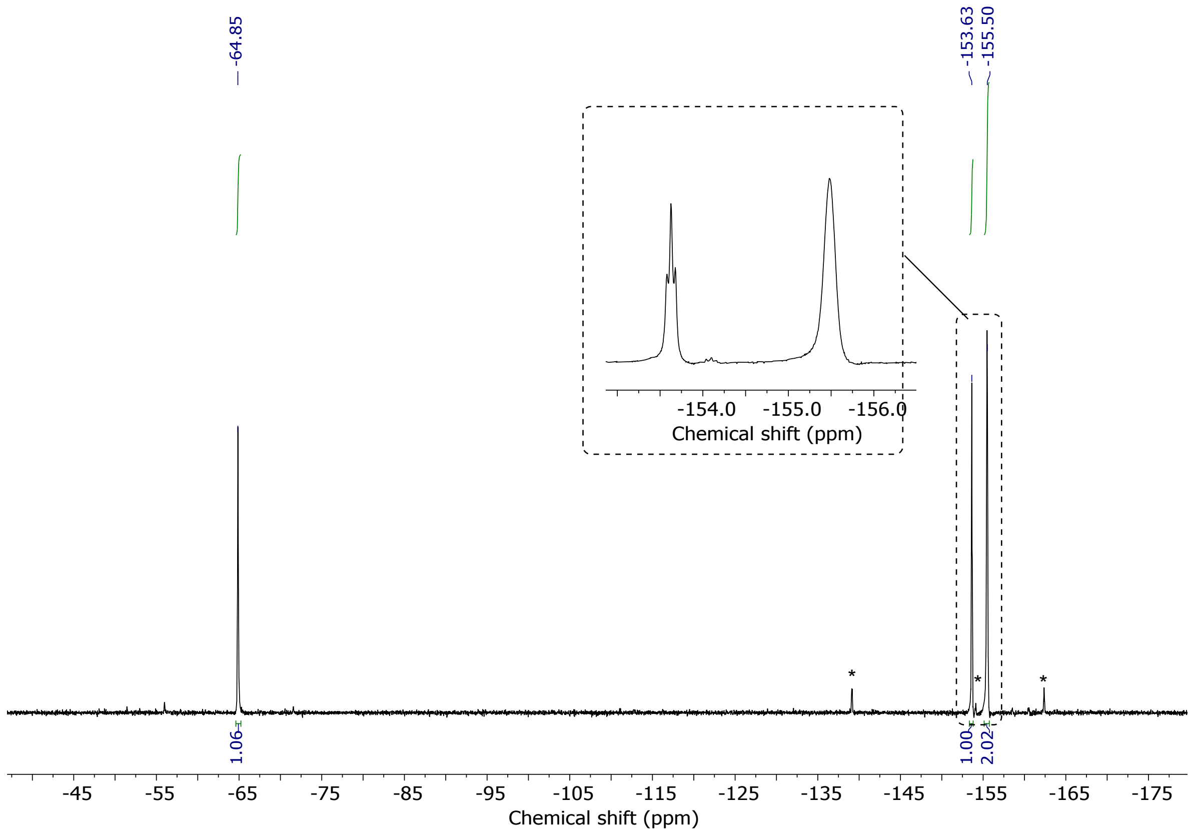

Figure S4: $^{19}F\{^{1}H\}$ NMR spectrum (toluene-d_8, 377 MHz) of complex 1. Trace HC_6F_5 denoted with *

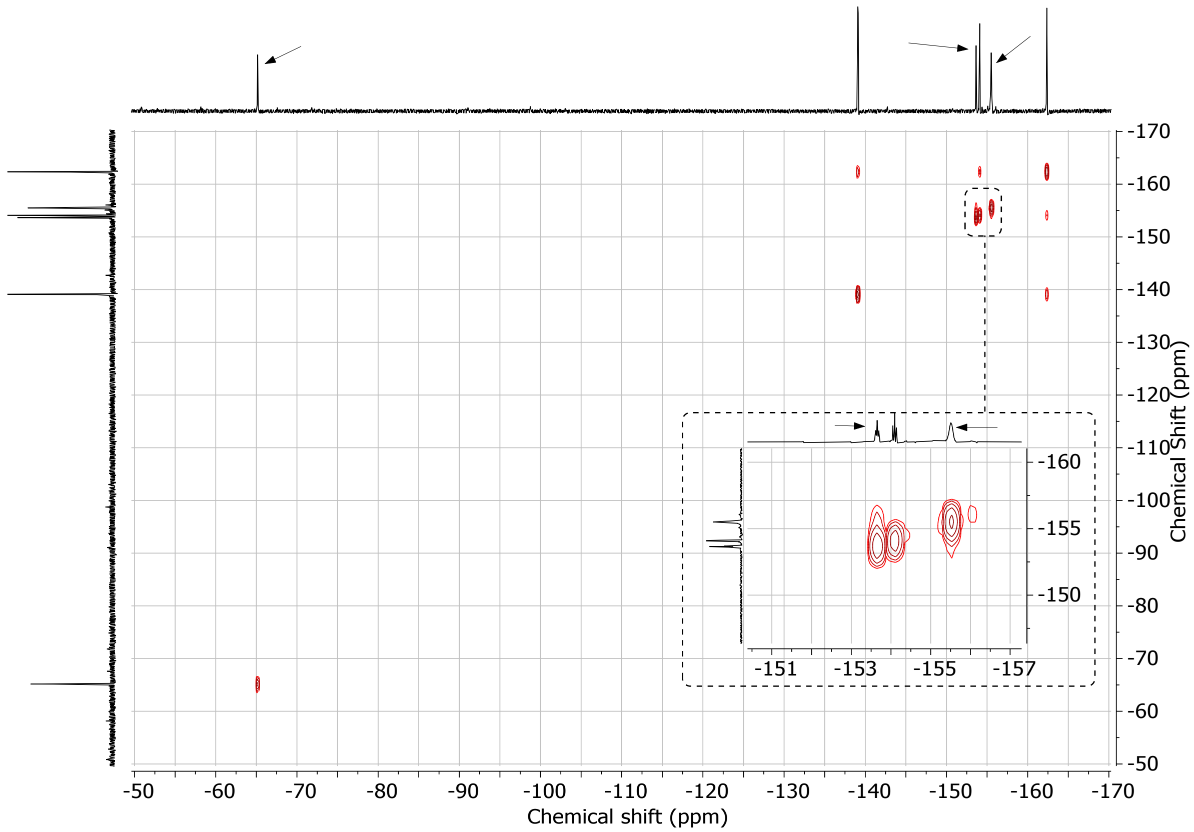

Figure S5: $^{19}F\{^{1}H\}$ COSY NMR spectrum (toluene-d_8, 377 MHz) of 1.Arrows indicate signals corresponding to 1; other signals are HC_6F_5 due to hydrolysis over the course of the measurement.

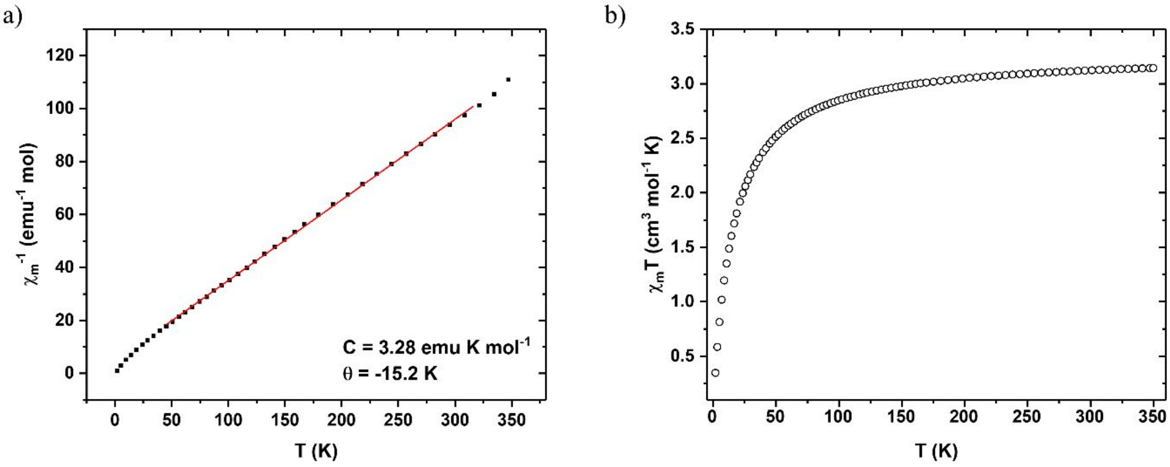

Figure S6: SQUID plots of complex **1**. a) Inverse susceptibility against temperature χ_m^{-1} *vs*. T for complex 10. From the linear fit, using the Curie-Weiss Law $\chi_m = \frac{C}{T-\theta}$, the Curie (C) and Weiss (θ) constants can be extracted. b) χ_mT *vs*. T plot for complex **1**. At room temperature, the effective magnetic moment (μ_{eff}) is $\mu_{eff} = 2.828\sqrt{\chi_m T} = 4.99\ \mu_B$. This is indicative of a high spin d^7 Co(II) complex with 2nd order spin-orbit coupling (μ_{so} = 3.87 μ_B for S=3/2, g = 2).

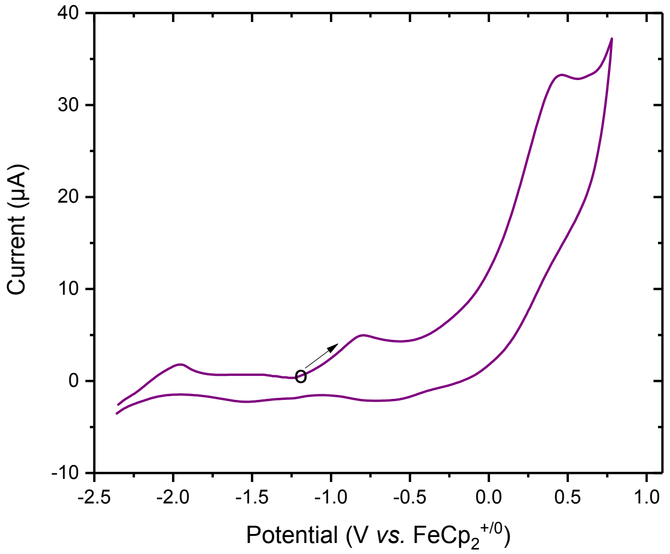

Figure S7: Cyclic voltammetry curve of 1 in THF. Conducted with 0.1 M [$^n Bu_4N$][PF_6] at a scan rate of 0.1 V s^{-1}, referenced to $FeCp_2^{+/0}$. Circle and arrow indicate start and direction of the measurement, respectively. The oxidation event at E_{pa} = −0.891 V and E_{pa} = −0.39 V are assigned to [$MgCo^{II}$]/[$MgCo^{III}$]$^+$ and phenolate ligand oxidation, respectively.

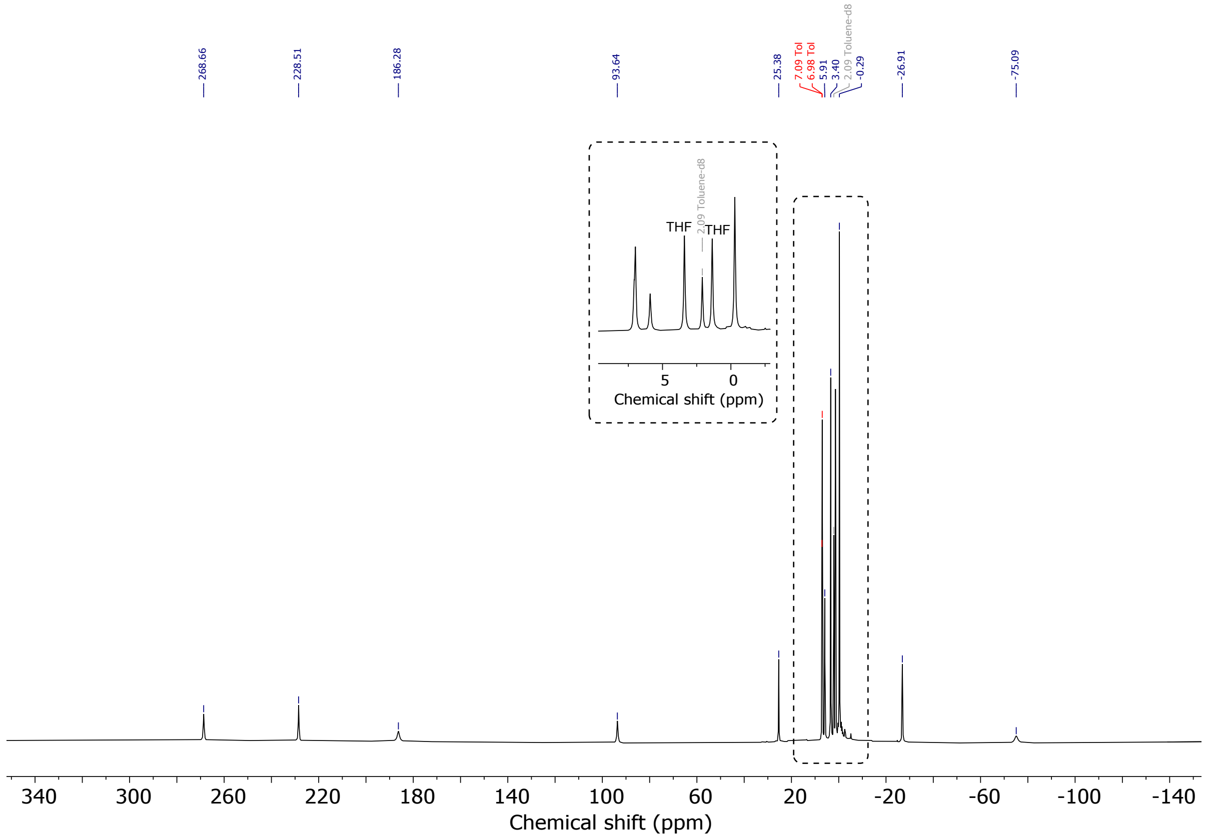

Figure S8: ^{1}H NMR spectrum (toluene-d_8, 400 MHz) of $[LCoCo(C_6F_5)_2]$ (**3**).

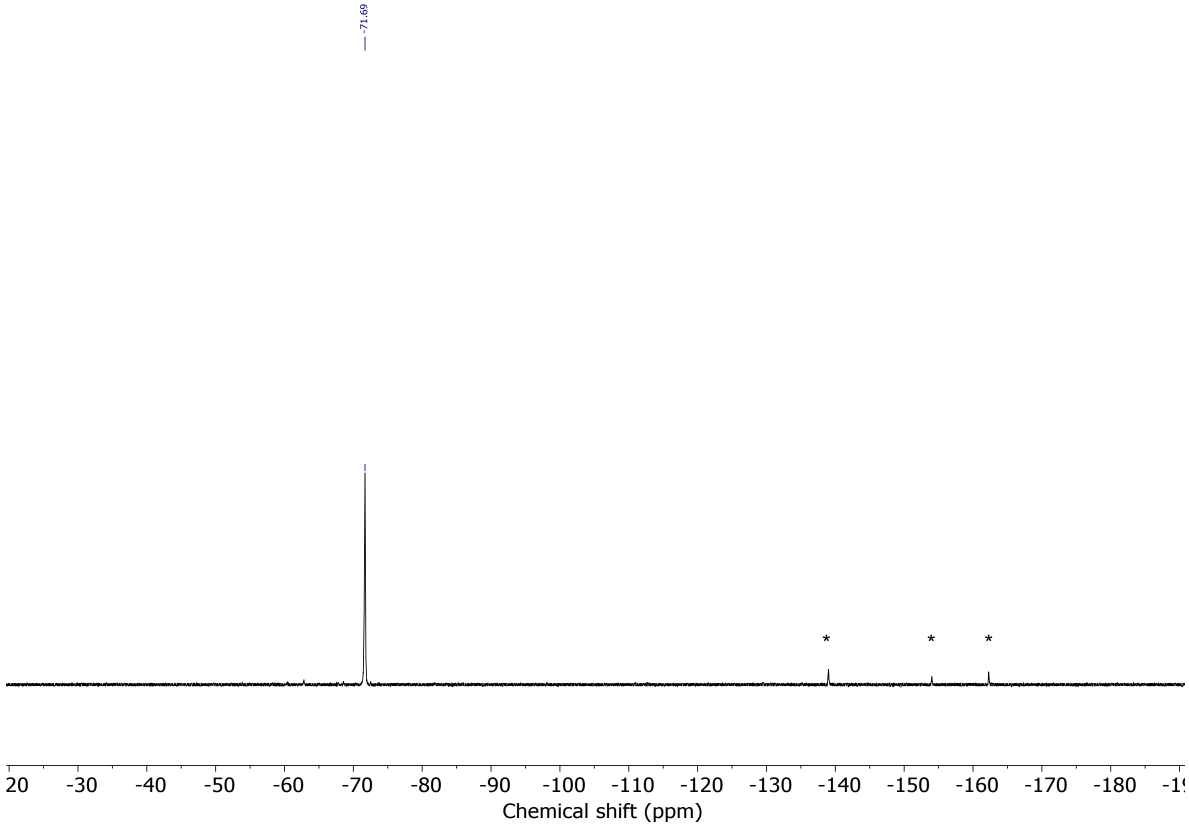

Figure S9: $^{19}F\{^{1}H\}$ NMR spectrum (toluene-d_8, 377 MHz) of **3**. Hydrolised ligand, HC_6F_5, denoted by *.

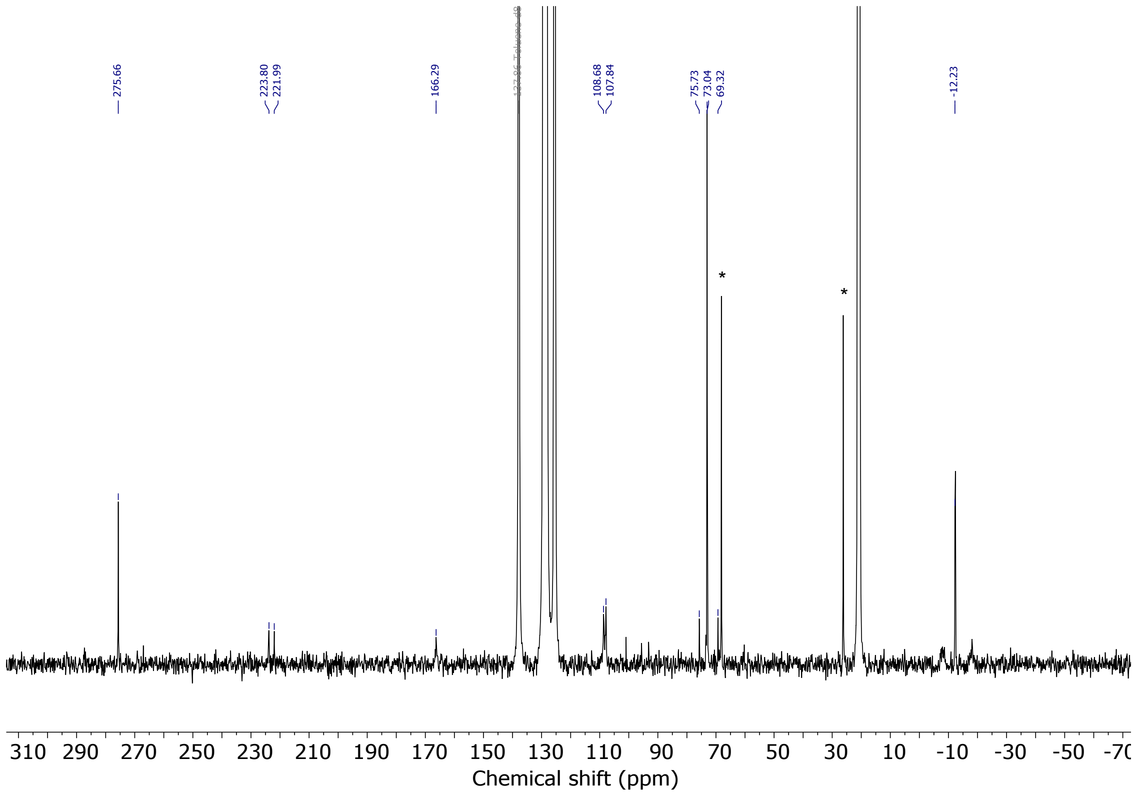

Figure S10: $^{13}C\{^1H\}$ NMR spectrum (toluene-d_8, 151 MHz) of **3**. * denotes resonance for THF.

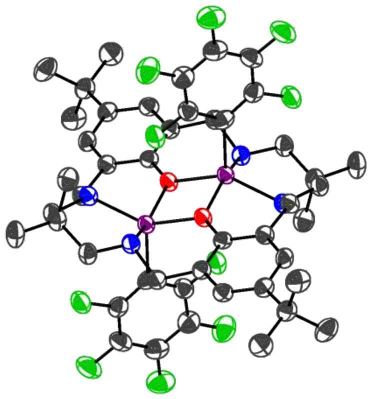

Figure S11: Molecular structure of **3**. H atoms and two toluene molecule have been omitted for clarity. Thermal ellipsoids at the 50 % probability level. Co = purple, O = red, N = blue, F = green, C = grey.

Table S3: Selected bond length and angles for complex **3**.

Bond	Length (Å)	Bond	Angle (°)
Co(1)-O(1)	2.046(2)	N(1)-Co(1)-O(2)	143.69(11)
Co(1)-O(2)	2.010(2)	N(2)-Co(1)-O(2)	83.44(11)
Co(1)-N(1)	2.115(3)	N(1)-Co(2)-O(1)	149.40(11)
Co(1)-N(2)	2.167(3)	N(2)-Co(2)-O(1)	83.25(11)
Co(1)-C(36)	2.104(4)		

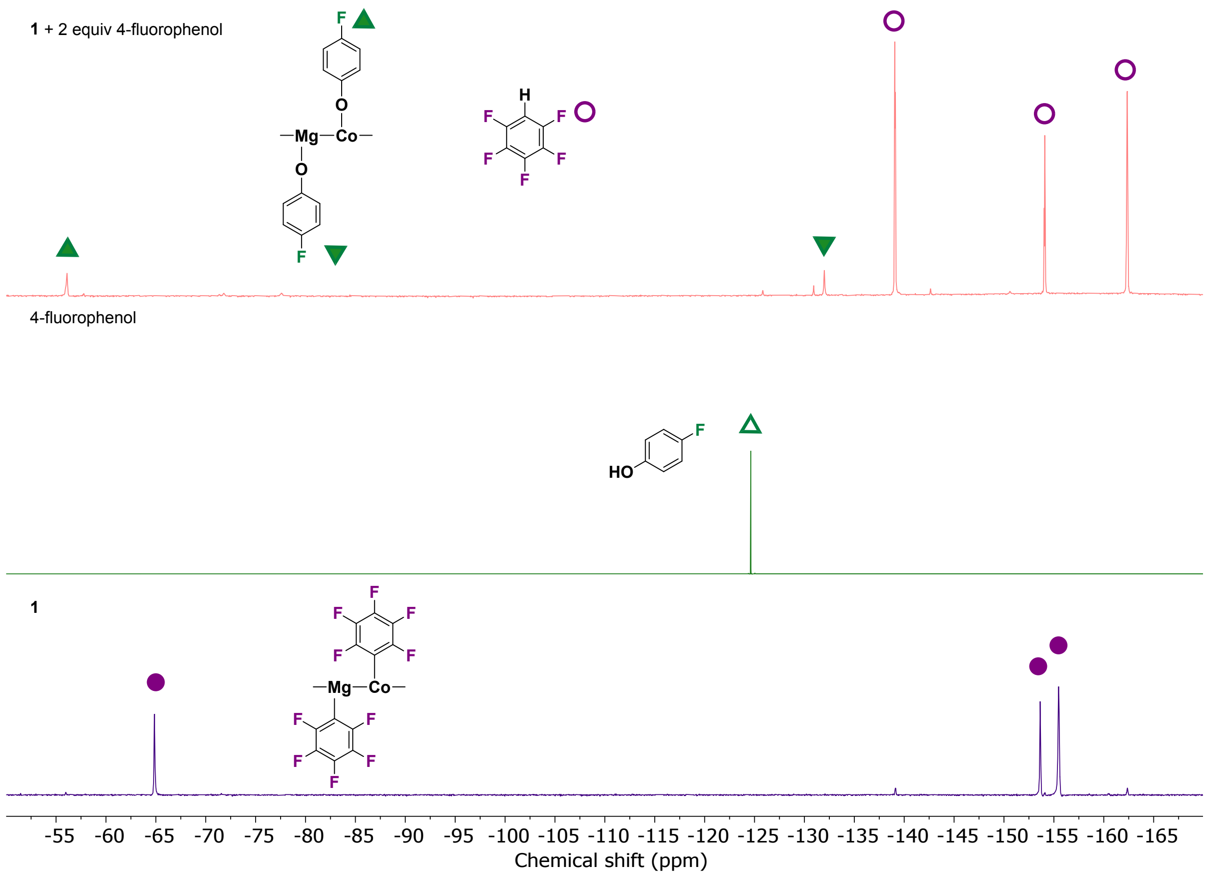

Figure S12: Stacked plot of $^{19}F\{^{1}H\}$ NMR spectra (toluene-d_8, 377 MHz) of **1** (bottom), 4-fluorophenol (middle), and the reaction between them (top).

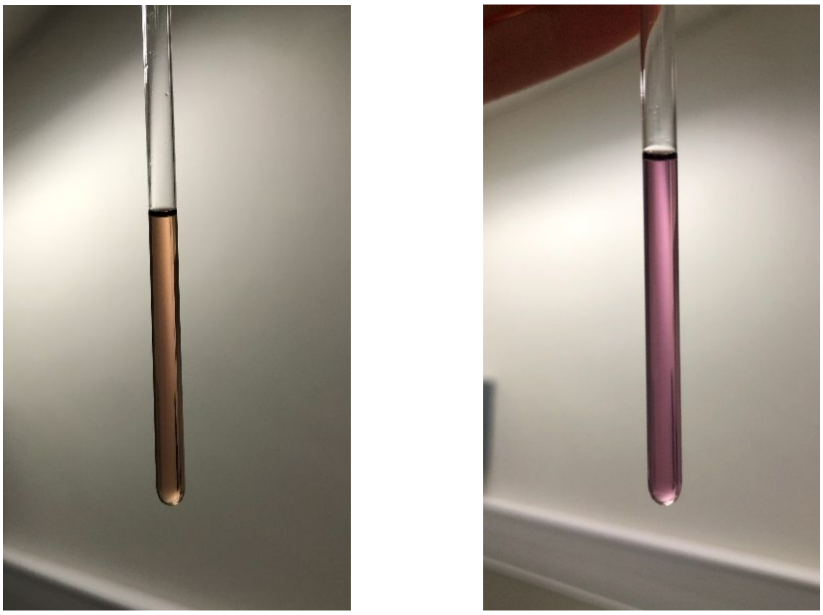

Figure S13: Solution of complex **1** in toluene-d_8 before (left) and after (right) addition of 2 equivalents of 4-fluorophenol. Colour change is instantaneous.

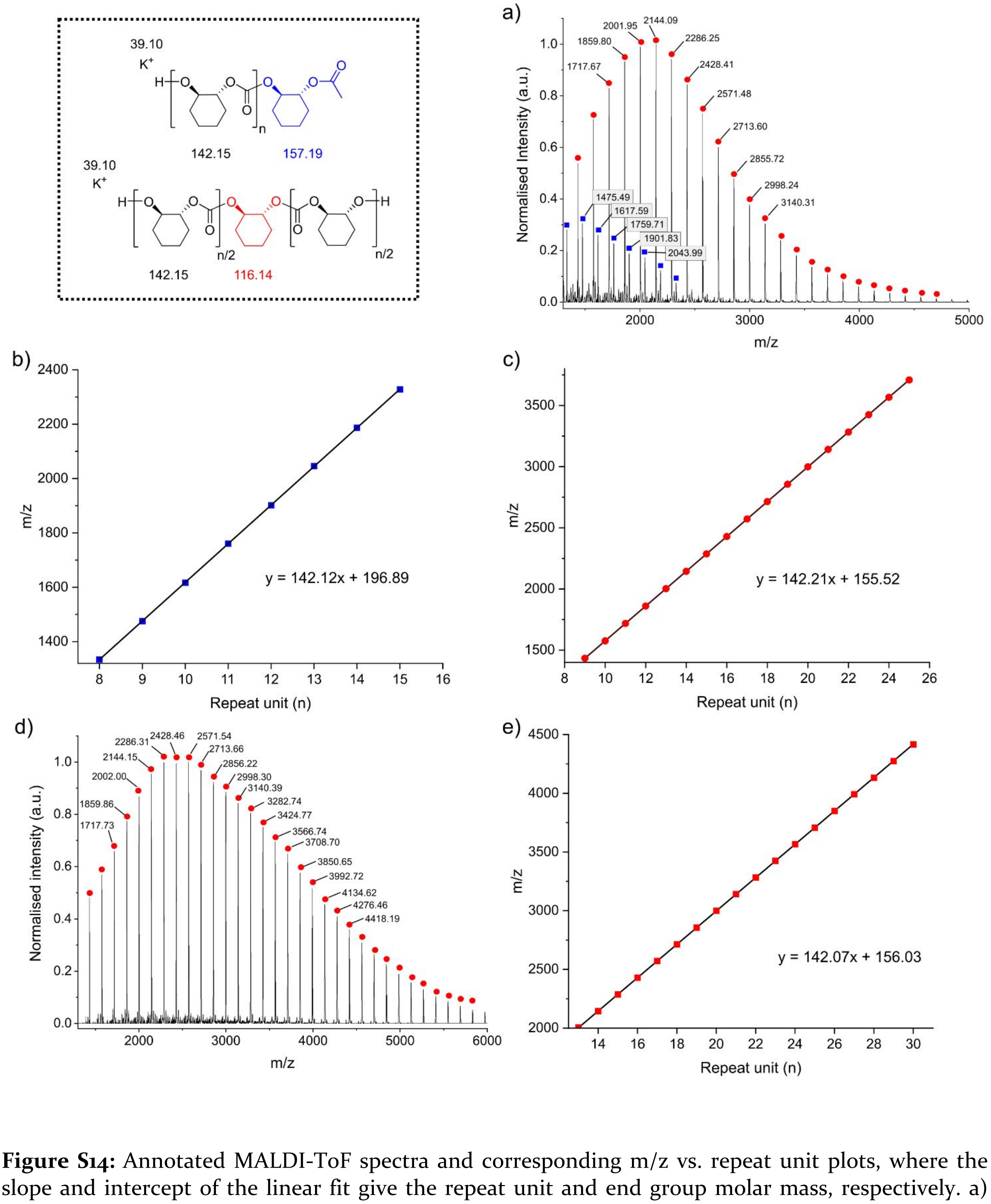

Figure S14: Annotated MALDI-ToF spectra and corresponding m/z vs. repeat unit plots, where the slope and intercept of the linear fit give the repeat unit and end group molar mass, respectively. a) MALDI-ToF spectrum of PCHC prepared using catalyst 2 marked with m/z peak values. b) Plot of m/z values against theoretical PCHC repeat unit from MALDI-ToF spectrum S14a. Distribution end-capped by acetate. c) CHD end-capped distribution. d) MALDI-ToF spectrum of PCHC prepared using catalyst 1 marked with m/z peak values. e) Plot of m/z values against theoretical PCHC repeat unit from MALDI-ToF spectrum S14d. Distribution end-capped by CHD.

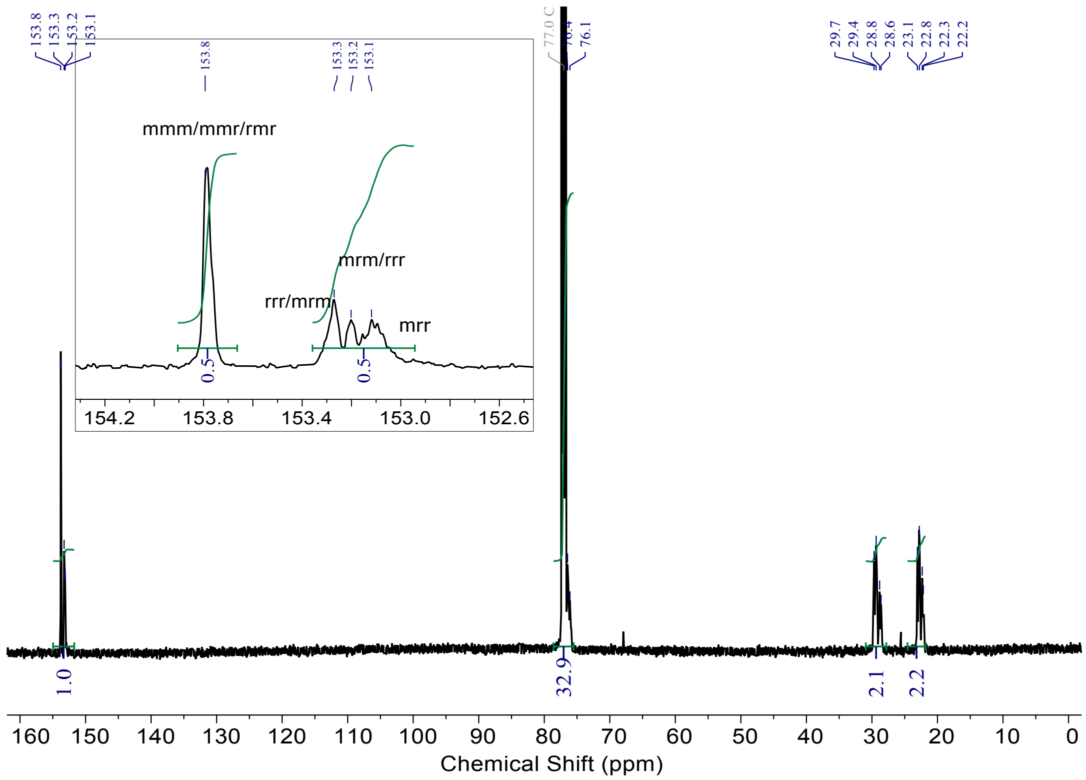

Figure S15: Quantitative ^{13}C NMR spectrum ($CDCl_3$, 151 MHz) of isolated PCHC catalysed by **1**. The polymer had a P_m value of 0.5 using the method reported by Coates *et al.*[13]

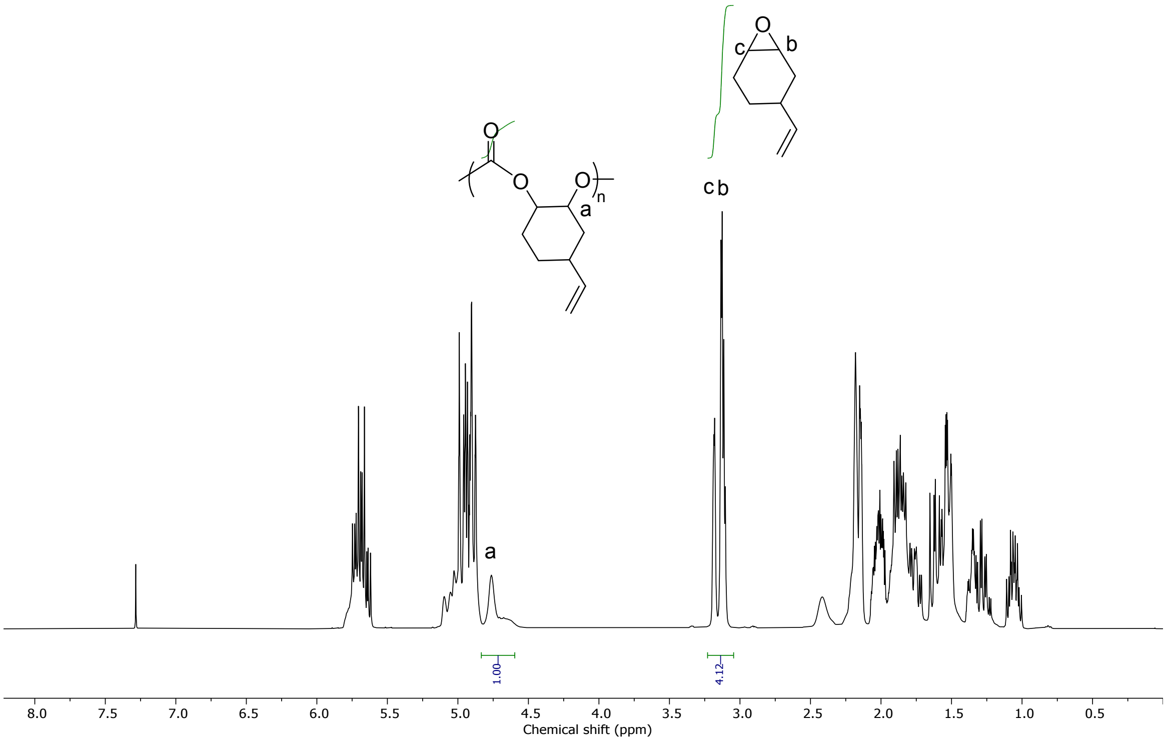

Figure S16: ^{1}H NMR spectrum ($CDCl_3$, 400 MHz) of polymerization aliquot. Conditions: [**1**]:[CHD]:[vCHO] = 1:4:2000, 1 bar CO_2, 100 °C, 1 h.

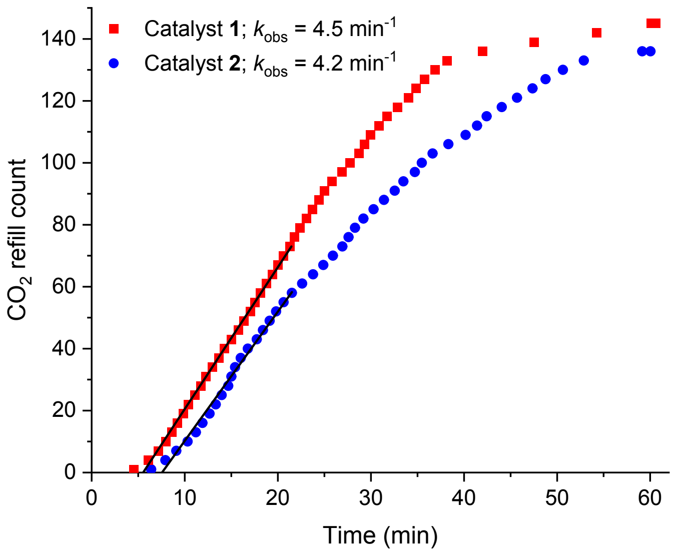

Figure S17: Initial rates comparison between catalyst **1** and **2**. Polymerization of vCHO/CO_2, 1 bar CO_2, 100 °C. The y-axis presents the CO_2 injection refills into the system. These re-fills occur when the pressure drops below 1 bar and relate directly to the catalyst activity.

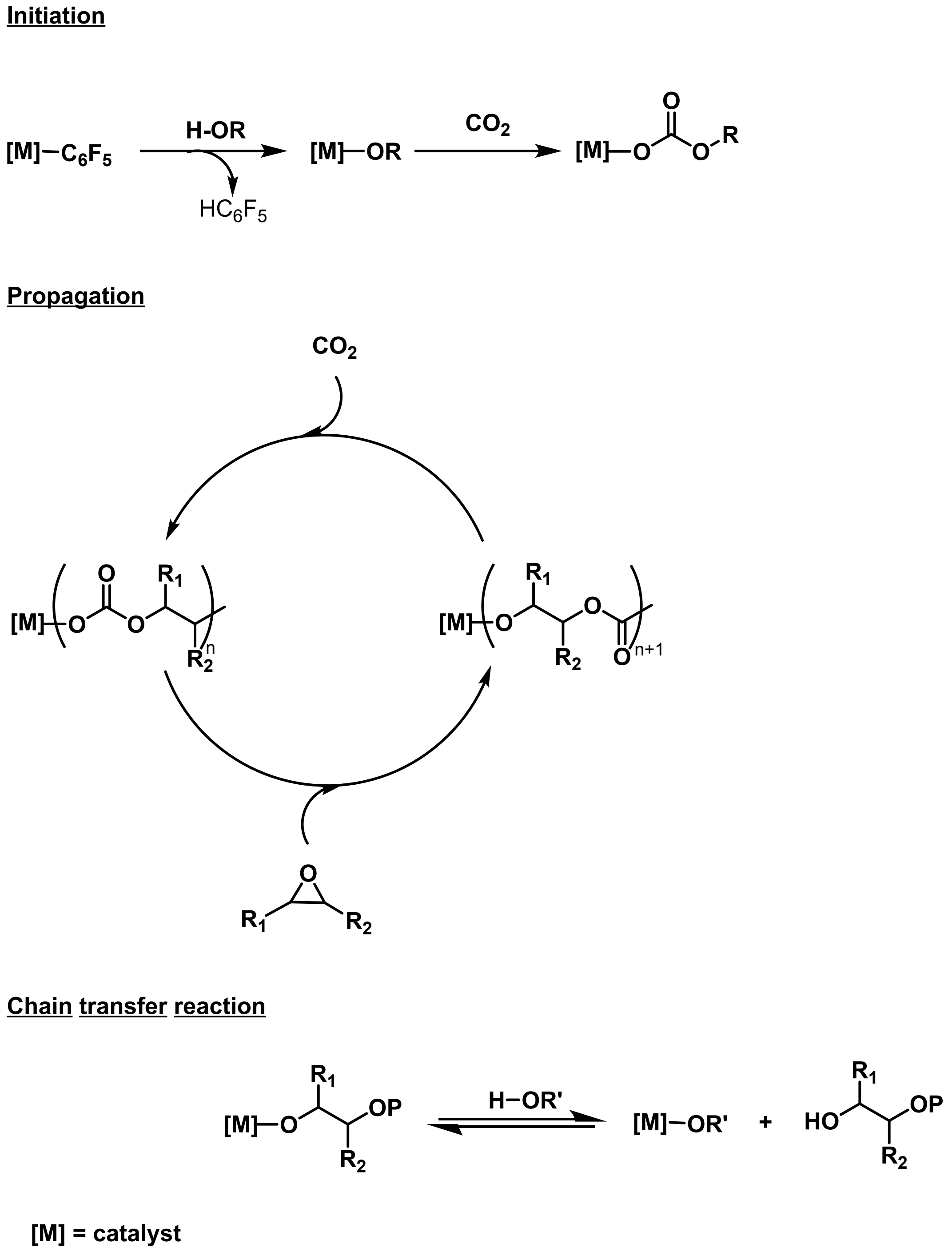

Figure S18: Illustration of epoxide/carbon dioxide ROCOP initiation, propagation for catalyst bearing the organometallic ligand, C_6F_5, and chain transfer reactions with alcohols.

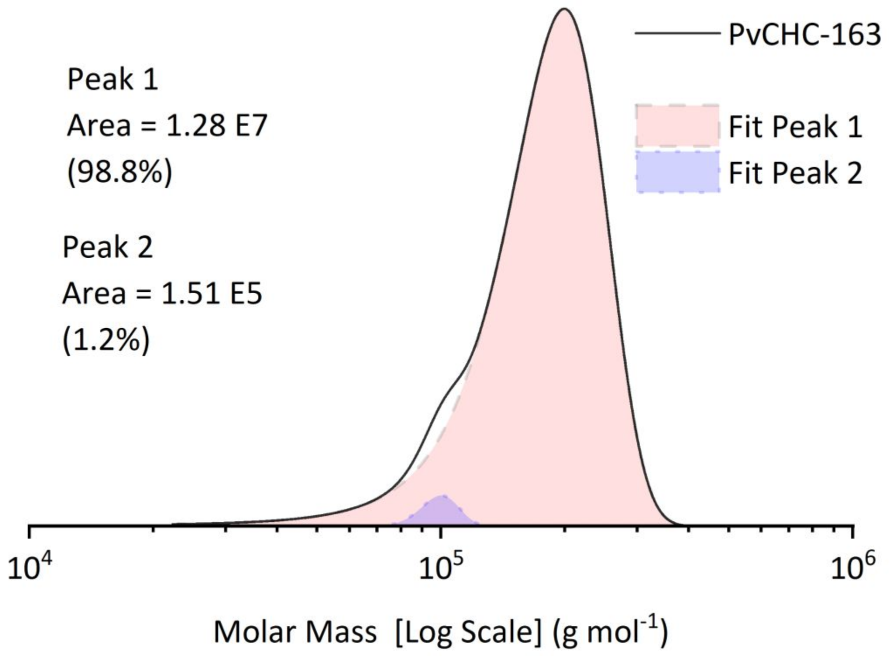

Figure S19: GPC analysis of PvCHC-163. The peaks were deconvoluted using Gaussian nonlinear curve fit on OriginPro 2023. Calculated M_n = 99, 182 and 163 kg mol^{-1} for peak 1, 2 and the cumulative fit respectively. The lower molar mass shoulder accounts for 1.2% of the total distribution, which we attribute to mass transfer limitations at high such high conversion/viscosity, causing the distribution to broaden.

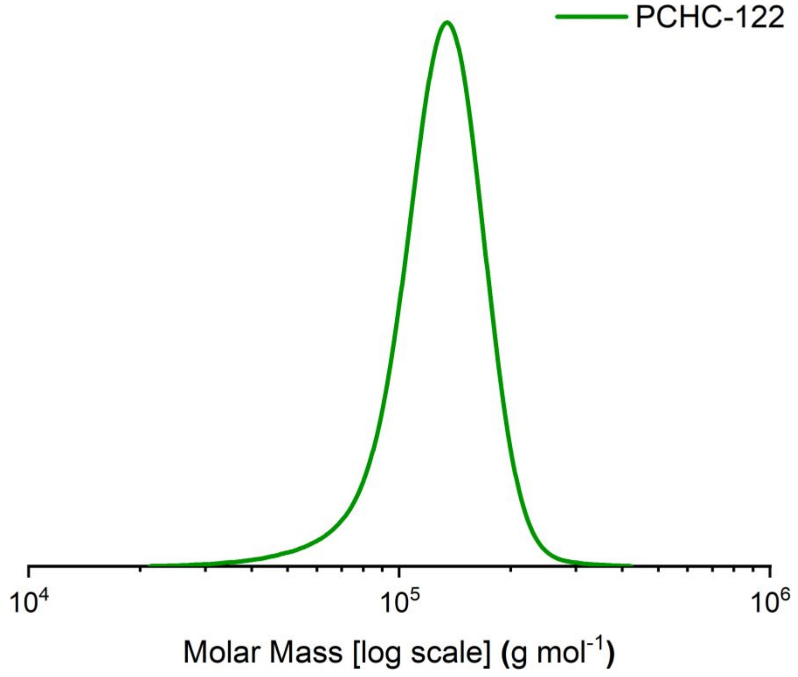

Figure S20: GPC analysis of PCHC-122.

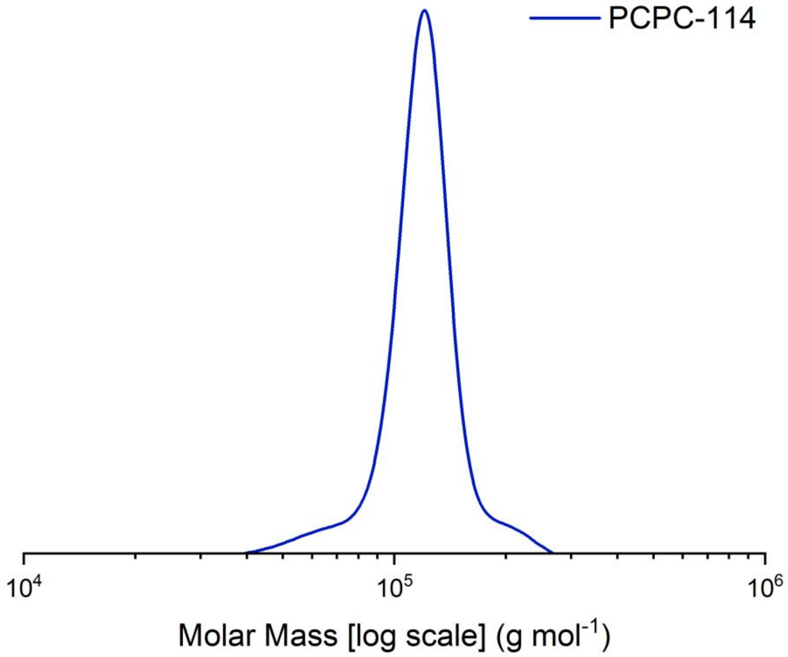

Figure S21: GPC analysis of PCPC-110.

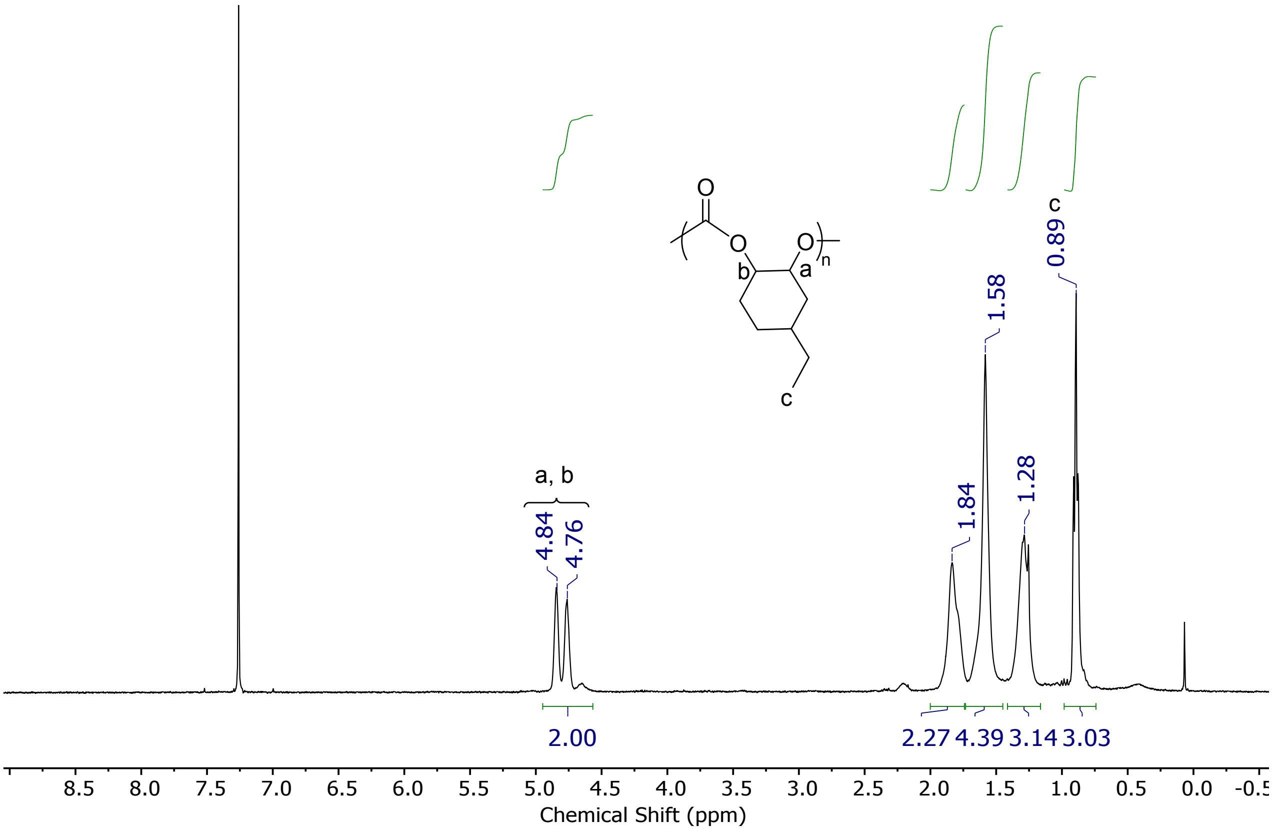

Figure S22: ^{1}H NMR spectrum ($CDCl_3$, 400 MHz) of PeCHC.

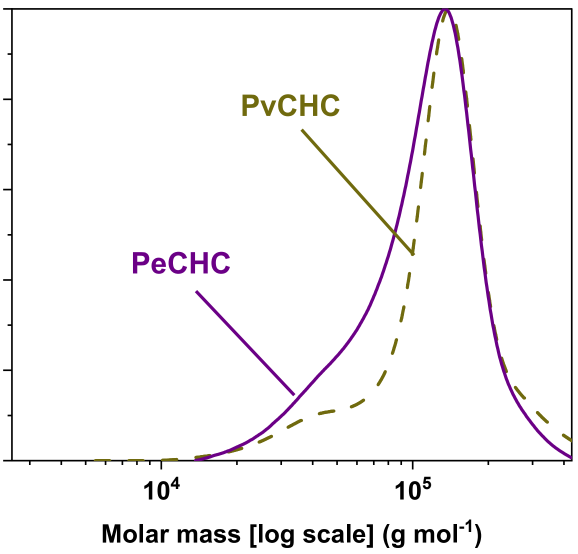

Figure S23: GPC analysis of PvCHC and PeCHC.

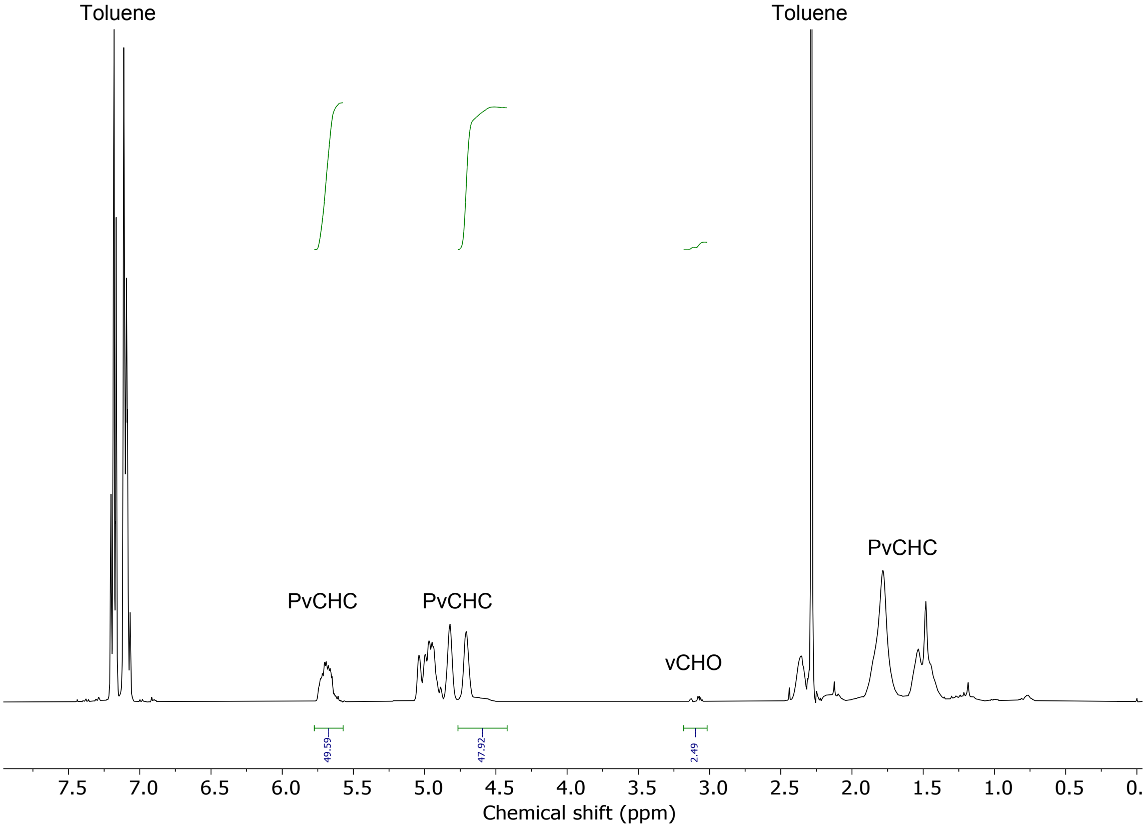

Figure S24: ^{1}H NMR spectrum ($CDCl_3$, 400 MHz) of an aliquot corresponding to Table 1, entry 5.

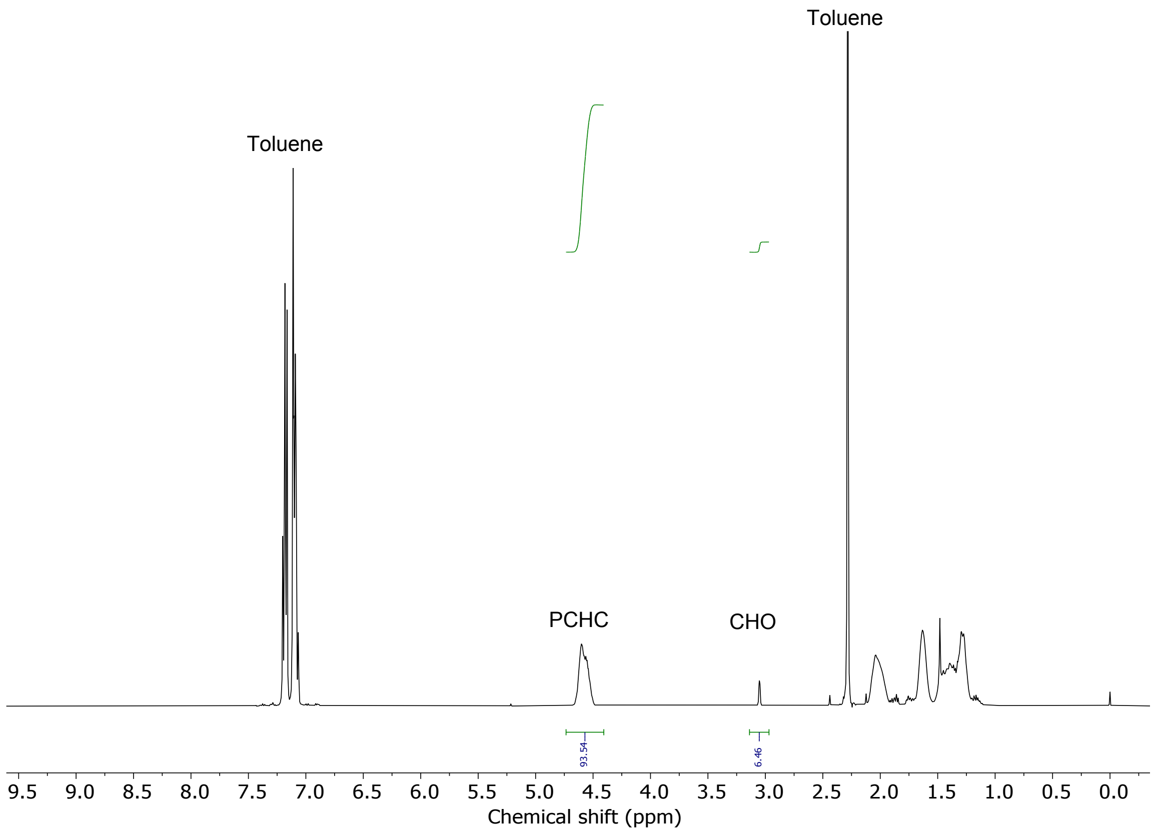

Figure S25: ^{1}H NMR spectrum ($CDCl_3$, 400 MHz) of an aliquot corresponding to Table 1, entry 7.

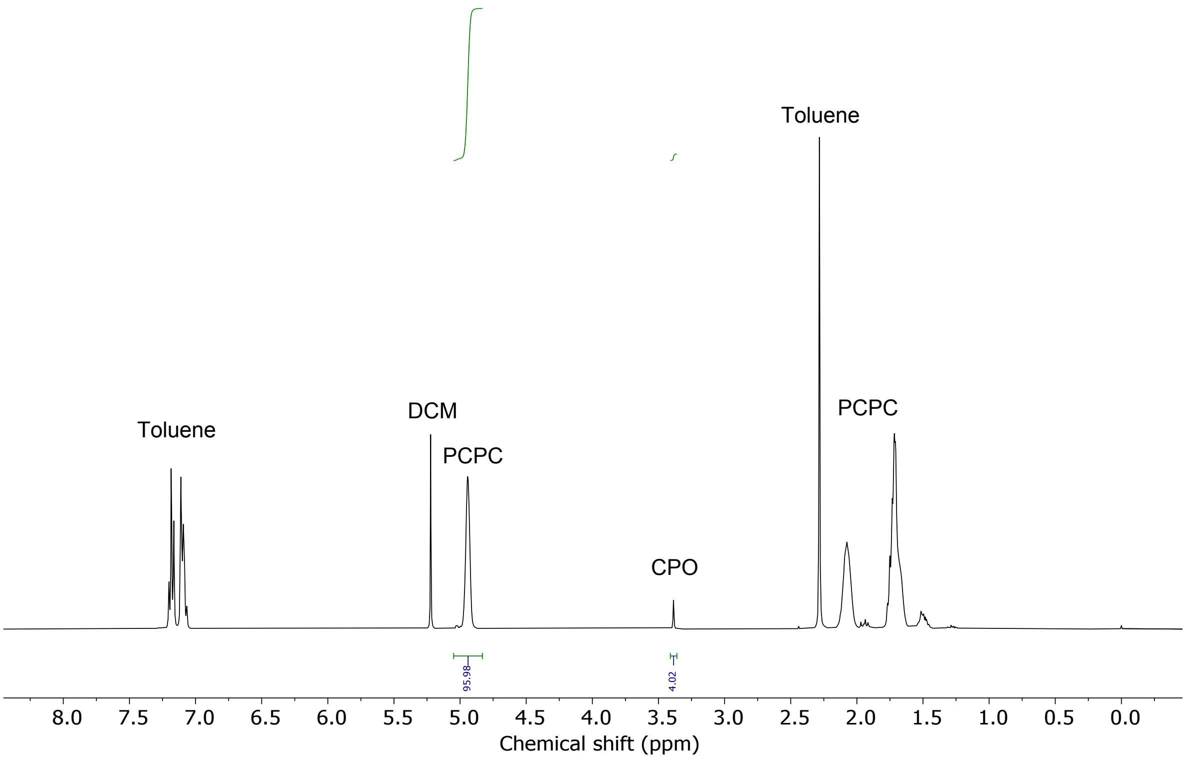

Figure S26: ^{1}H NMR spectrum ($CDCl_3$, 400 MHz) of an aliquot corresponding to Table 1, entry 8.

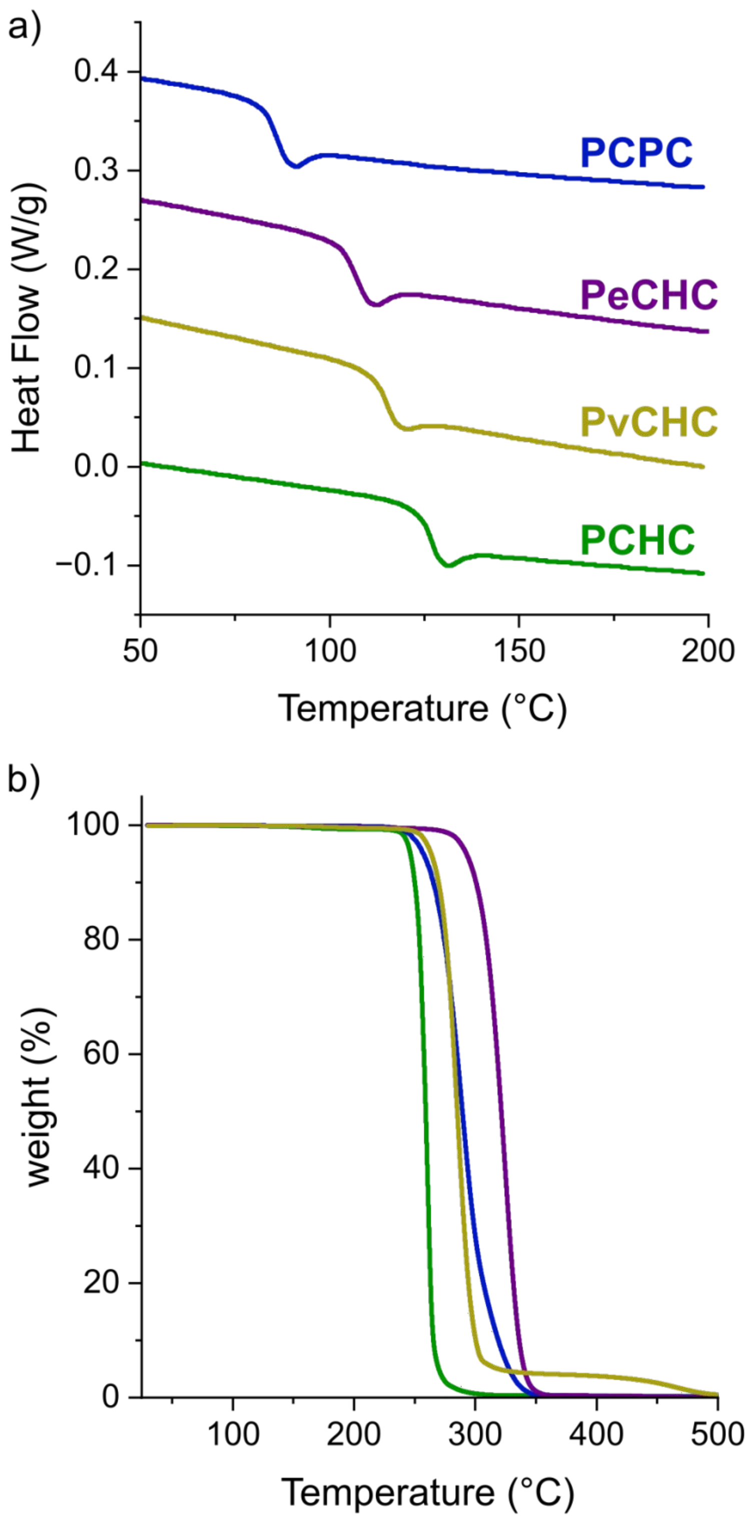

Figure S27. Overlay of DSC data (a) and TGA data (b) for PCPC-114 (blue), PeCHC-125 (purple), PvCHC-125 (dark yellow), and PCHC-122 (green).

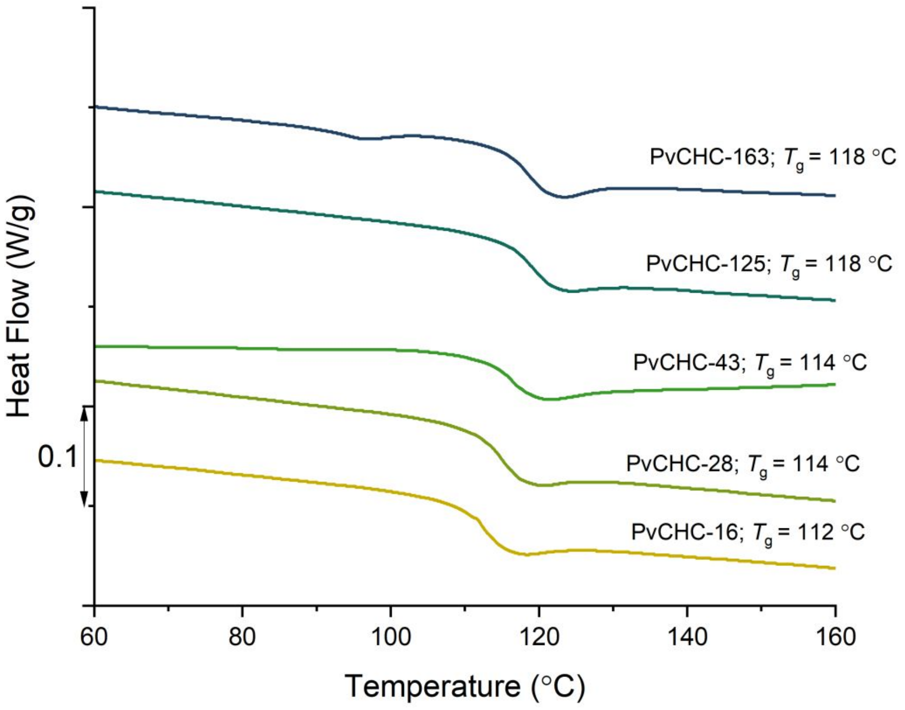

Figure S28: DSC data for PvCHC with increasing M_n.

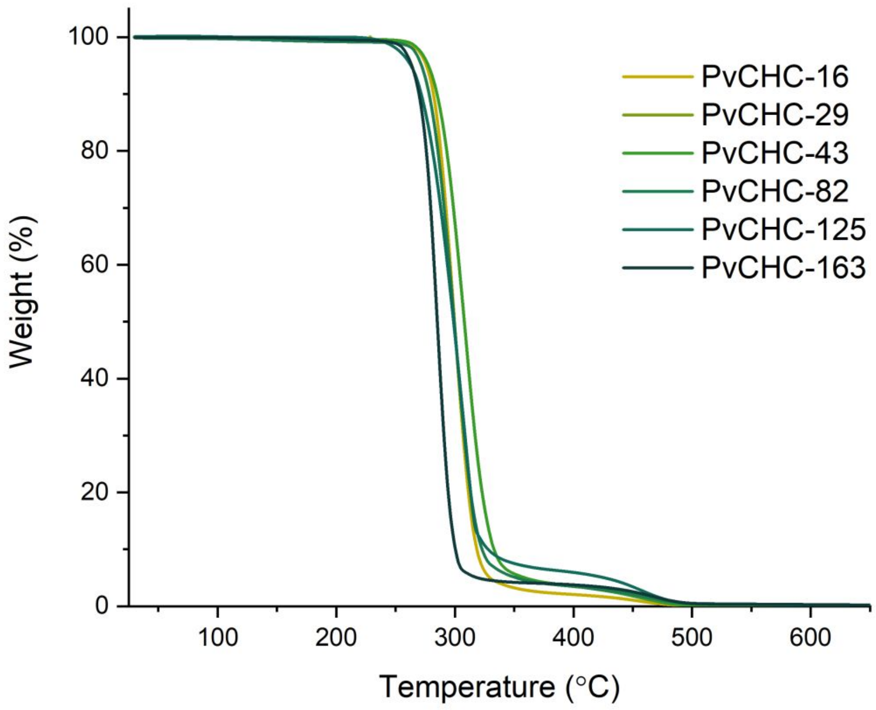

Figure S29: Overlay of TGA data for PvCHC series.

Rheology Measurements and Time-Temperature Superposition (TTS).

Oscillatory shear measurements were performed on an ARES-G2 rheometer (TA Instruments) between two stainless steel 8 mm parallel plates. Clear round disks 8 mm in diameter were punch-cut with a hole borer from hot-pressed homogeneous films (0.7–1.0 mm thickness). The specimen disks were loaded onto the equilibrated geometry at the highest running temperature, except for disks of PvCHC thermoplastic which were loaded at 120 °C and equilibrated at 150 °C, under a stream of N_2 inside of the rheometer's furnace. Axial force was allowed to relax while taking the geometry gap to 750-1000 μm (trim gap offset of 50 μm). Dynamic tests were performed in the viscoelastic linear region after performing dynamic strain sweeps. Stress relaxation experiments were performed at 15 % oscillatory strain, after 2 min equilibration, at various temperatures. Oscillatory temperature ramps (0.5% strain, 1.0 Hz, 2 °C · min^{-1}) were performed on cooling from the maximum running temperature, except for samples of PvCHC which were tested on heating to avoid spontaneous crosslinking at high temperatures. Consecutive oscillatory frequency sweeps (0.5% strain, 0.01–100 Hz) were performed at 10 °C intervals, with 1 min equilibration time between runs. The data was successfully combined into the corresponding time-temperature superposition (TTS) mastercurves with the TRIOS Software Version 4.2.1 (TA Instruments), and referenced to 140 °C as indicated in Table 5. The data and figures represent the geometric average of four or five identical runs.

The glass transition temperature ($T_{g,rheo}$) was obtained from the peak of the tan(δ) curve, while the crossover temperature ($T_{g,rheo}$) was obtained as the temperature at which *G'* = *G"* in the temperature ramps. The zero-shear viscosity of the polymers, η_o, was taken from the low-frequency plateau of the mastercurves. The time at the crossover modulus, τ, was obtained as the inverse of the frequency (in Hz) at which *G'* = *G"* in the mastercurves. The shift-factor, α_T, with which the mastercurves were constructed were successfully fitted to the Williams–Landel–Ferry (WLF) equation:

$$\log(\alpha_T) = \frac{-C_1(T - T_{ref})}{C_2 + T - T_{ref}}$$

where T_{ref} is the reference temperature (110 °C or 140 °C) used to construct the mastercurves.

The plateau modulus, G^o_N, was determined from the value located at the minimum of the tan(δ) curve in the TTS mastercurves, which then was used to calculate the entanglement MW (M_e) according to the equation:

$$M_e = \frac{4\rho RT}{5\ G_N^0}$$

where R is the gas constant (8.314 m^3·Pa·K^{-1}·mol^{-1}), T is the reference temperature in Kelvin (see Table 1), and ρ is the melt density (ρ = 1135 kg·m^{-3} for PCHC,[14] when unknown, calculations assume a range of 800–1100 kg·m^{-3} to provide an estimate).[15]

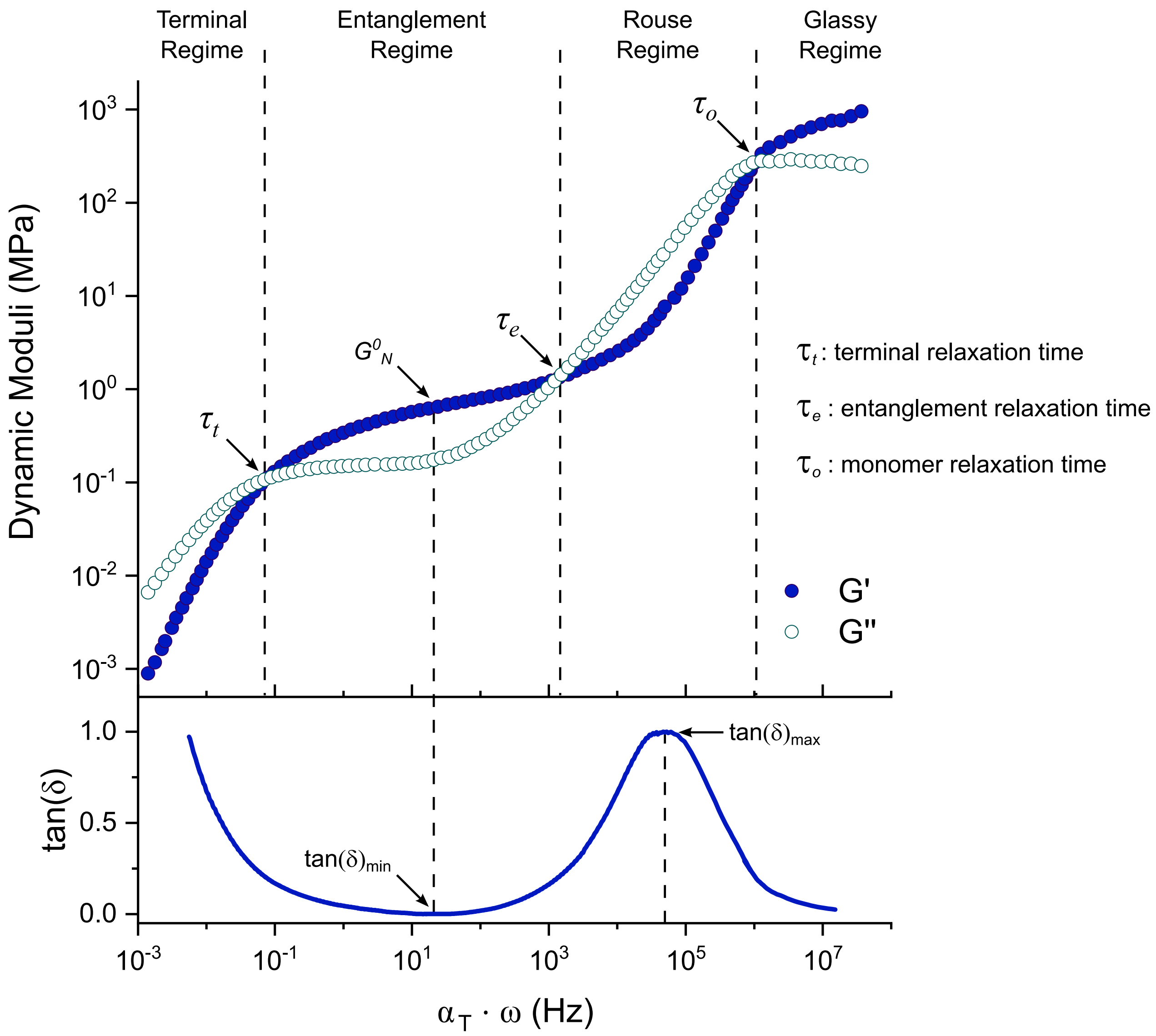

Figure S30: Representative TTS master curve of PCPC-114(Figure S32) displaying different relaxation regimes, relaxation times (τ_t, τ_e, and τ_o, obtained from the crossover where G'=G"), and the plateau modulus ($G^o{}_N$, obtained from the minimum of the tan(δ) curve).

Table S4: Viscoelastic parameters obtained from the time-temperature superposition mastercurves (T_{ref} = 140 °C).

Polymer	TTS temp. interval (°C)	terminal crossover (MPa)	τ_t (min)	entanglement crossover (MPa)	τ_e (s)	glassy crossover (MPa)	τ_o (ms)	$\tan(\delta)_{max}$ (Hz)
PCPC-114	90 – 180	0.121 ± 0.008	0.21 ± 0.02	1.35 ± 0.05	(72 ± 5) × 10^{-5}	250 ± 20	(106 ± 15) × 10^{-5}	42000 ± 5000
PeCHC-120	120 – 200	0.055 ± 0.003	0.57 ± 0.03	0.42 ± 0.03	0.195 ± 0.006	88 ± 2	0.1418 ± 0.0009	32.7 ± 1.6
PvCHC-125[a]	140 – 200	0.013 ± 0.002	87 ± 18	0.13 ± 0.02	1.33 ± 0.18	n.o.	n.o.	70 ± 11
PCHC-122	130 – 220	0.013 ± 0.001	240 ± 30	0.137 ± 0.015	32 ± 5	66 ± 10	7.7 ± 1.8	4.3 ± 0.4

[a] Sample containing 0.1 wt% of a thermal stabilizer. (n.o. = not observed)

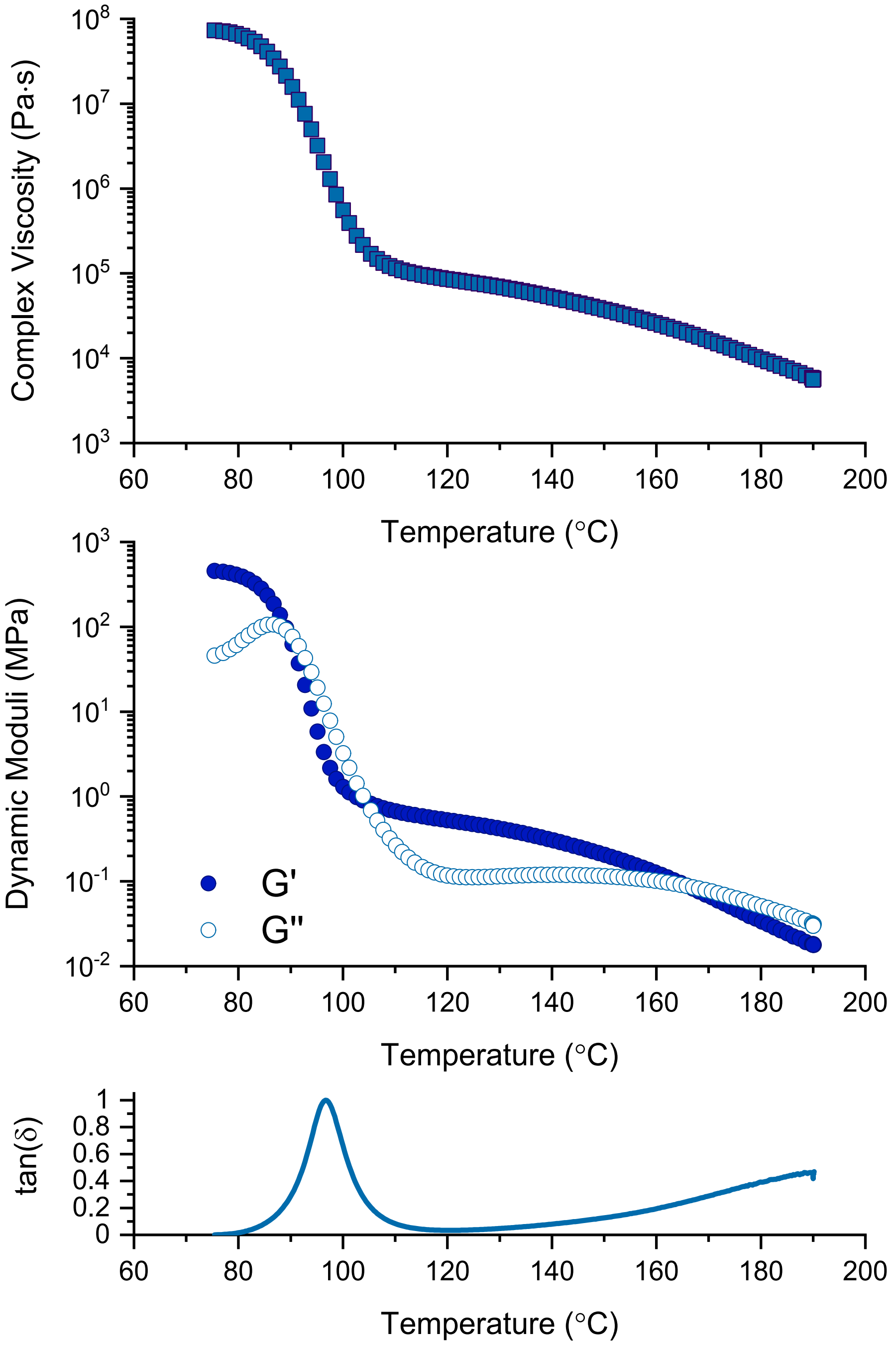

Figure S31 Oscillatory temperature ramp of PCPC-114 (0.5% strain, 1.0 Hz, 2 °C · min^{-1}) between 75 and 190 °C. Top: complex viscosity, η*; middle: dynamic moduli, G' and G"; bottom: tan(δ).

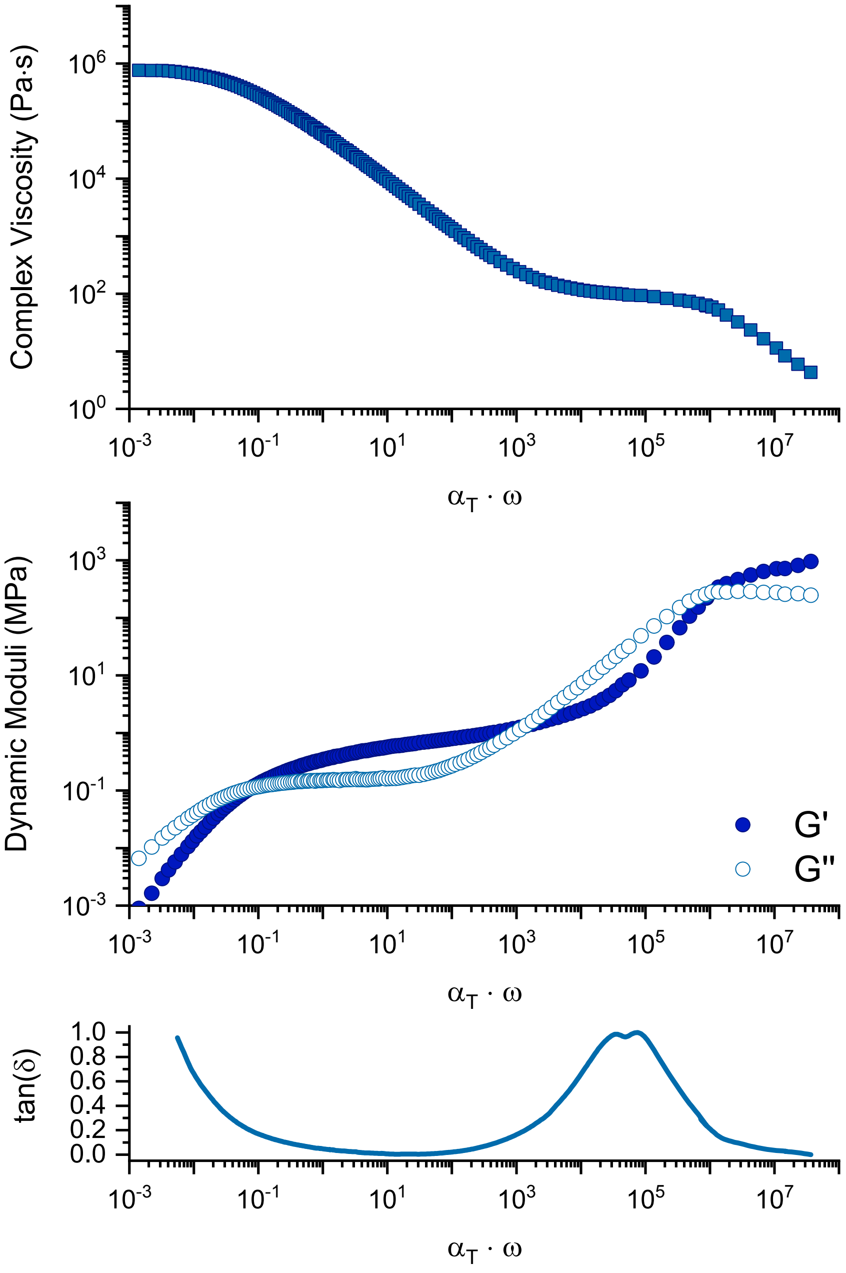

Figure S32: Mastercurves of PCPC-114 constructed by time-temperature superposition (TTS) referenced to 140 °C (0.5% strain, 0.01–100 Hz). The data points are the geometric average of four identical runs. Top: complex viscosity, η*; middle: dynamic moduli, G' and G"; bottom: tan(δ).

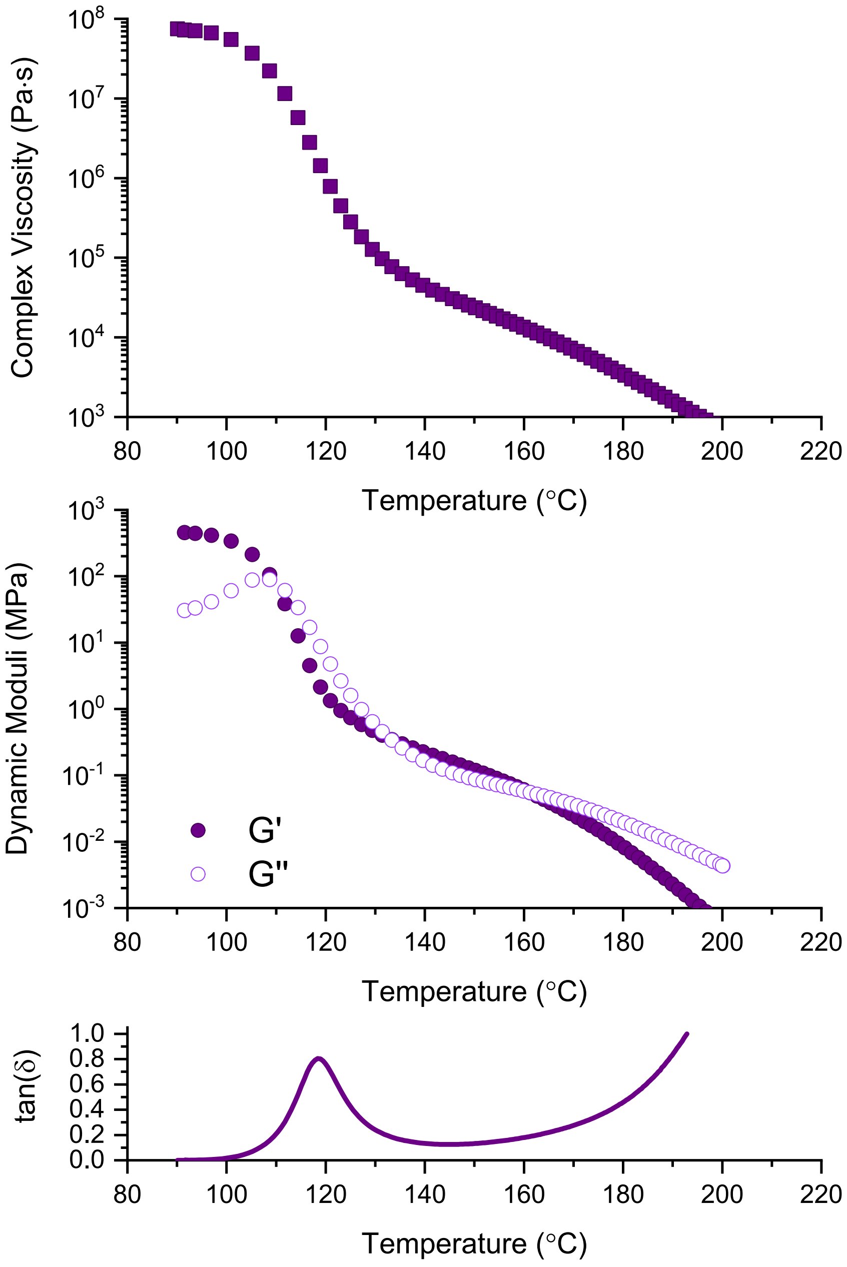

Figure S33: Oscillatory temperature ramp of PeCHC-125 (0.5% strain, 1.0 Hz, 2 °C · min^{-1}) between 90 and 200 °C. Top: complex viscosity, η*; middle: dynamic moduli, G' and G"; bottom: tan(δ).

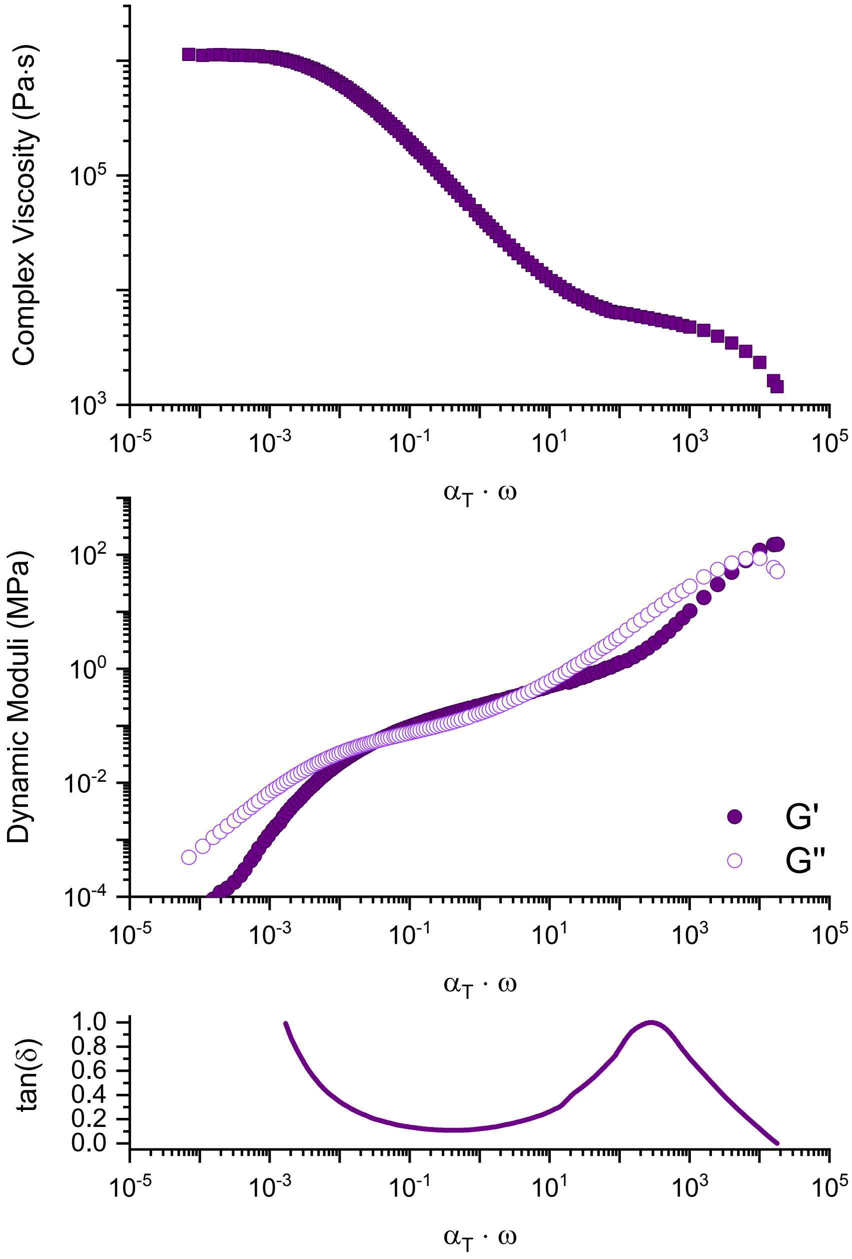

Figure S34: Mastercurves of PeCHC-125 constructed by time-temperature superposition (TTS) referenced to 140 °C (0.5% strain, 0.01–100 Hz). The data points are the geometric average of five identical runs. Top: complex viscosity, η*; middle: dynamic moduli, G' and G"; bottom: tan(δ).

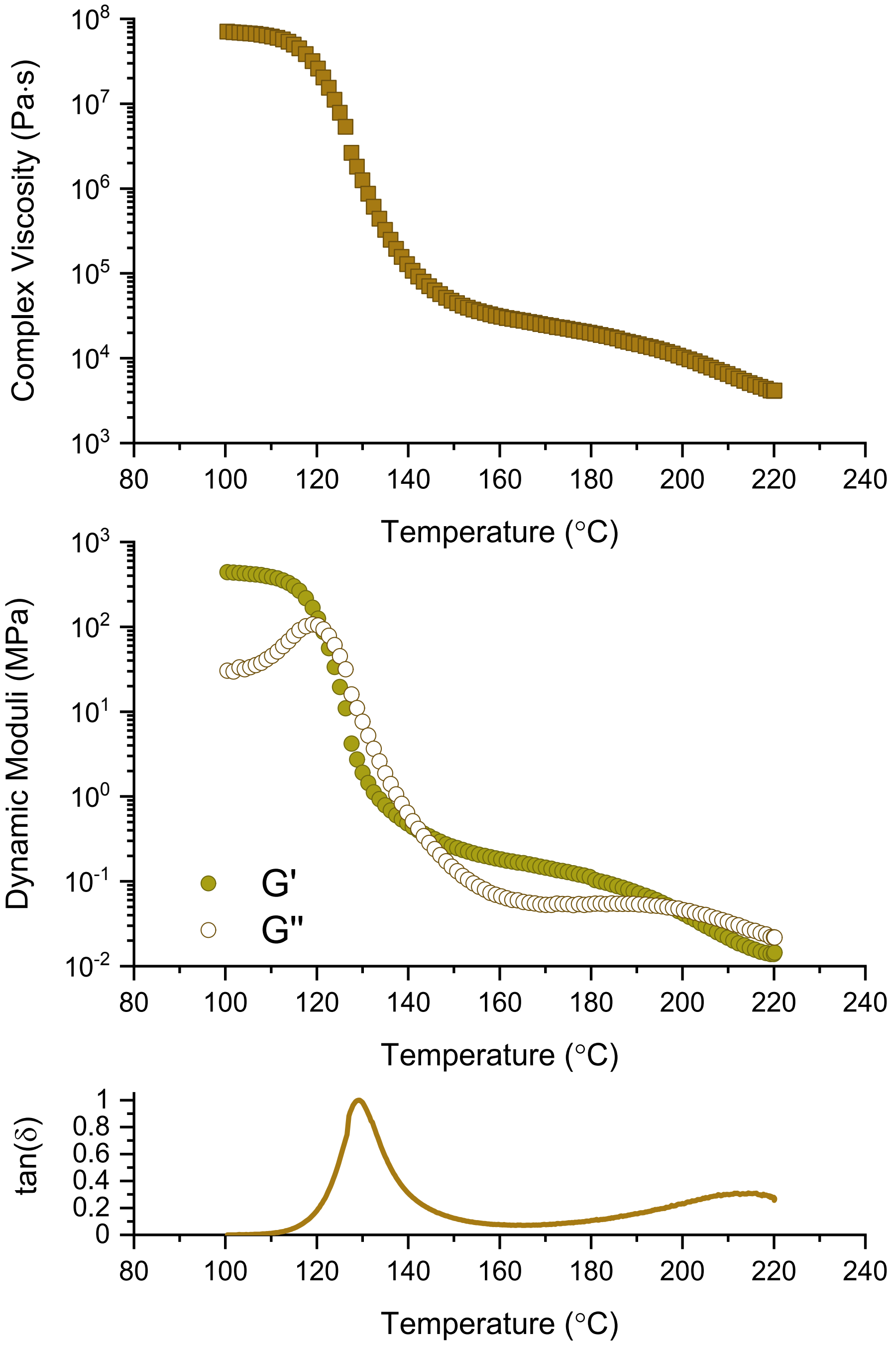

Figure S35: Oscillatory temperature ramp of PvCHC-125 containing 0.1 wt% of a thermal stabilizer (0.5% strain, 1.0 Hz, 2 °C · min^{-1}) between 100 and 220 °C. Top: complex viscosity, η*; middle: dynamic moduli, G' and G"; bottom: tan(δ).

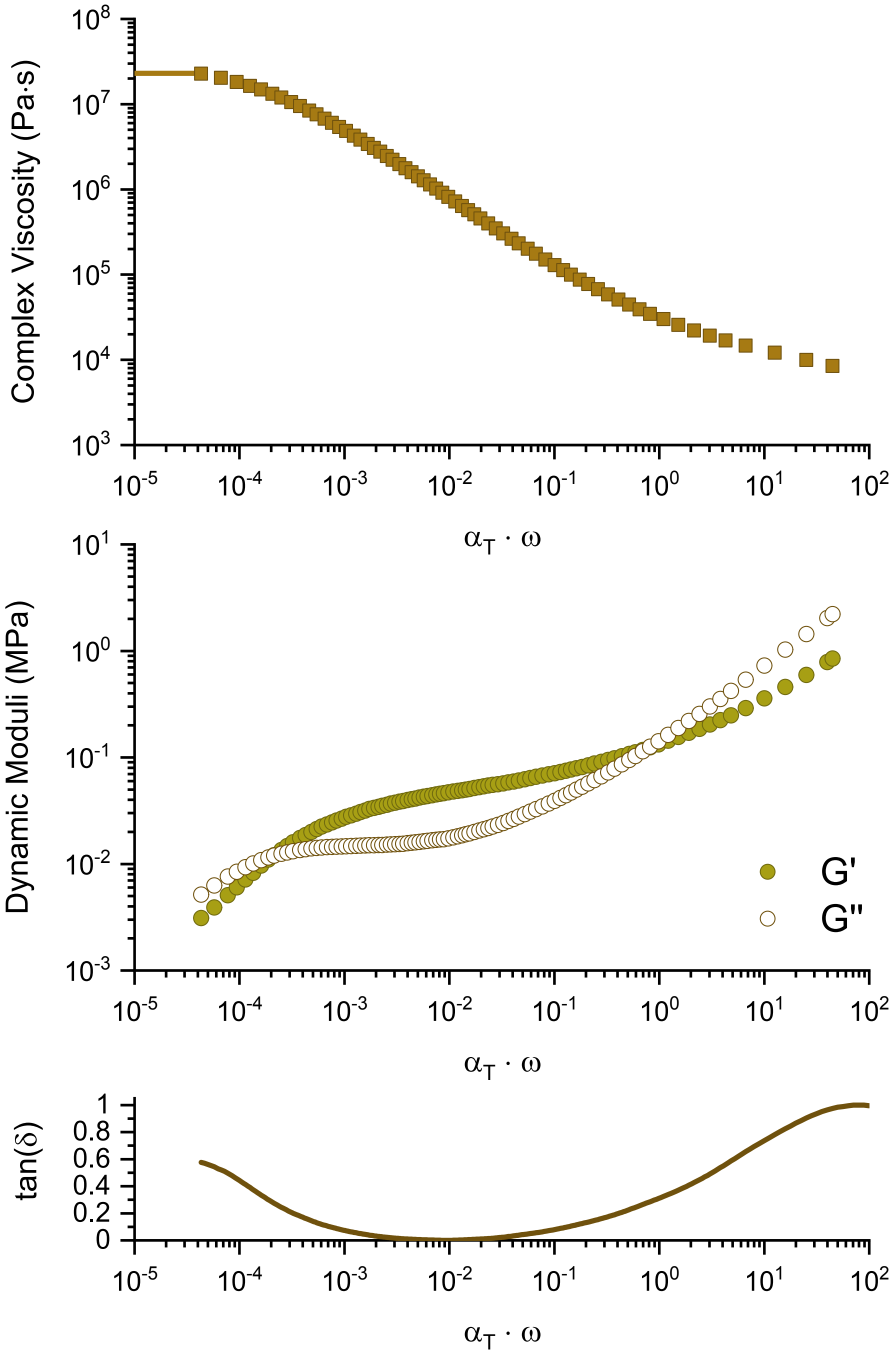

Figure S36: Mastercurves of PvCHC-125 containing 0.1 wt% of a thermal stabilizer constructed by time-temperature superposition (TTS) referenced to 140 °C (0.5% strain, 0.01–100 Hz). The data points are the geometric average of five identical runs. Top: complex viscosity, η*; middle: dynamic moduli, G' and G''; bottom: tan(δ).

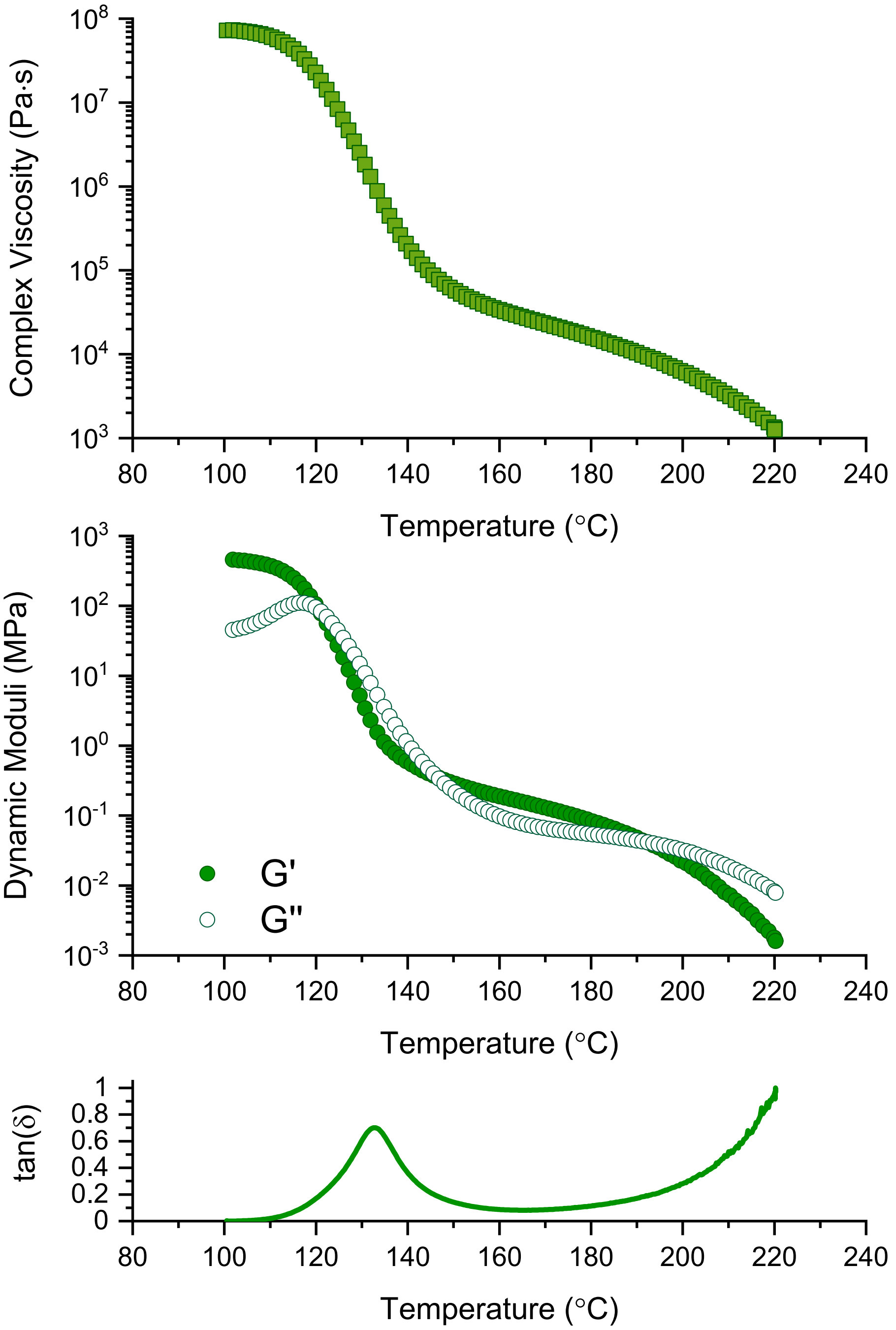

Figure S37: Oscillatory temperature ramp of PCHC-122 (0.5% strain, 1.0 Hz, 2 °C · min^{-1}) between 100 and 220 °C. Top: complex viscosity, η*; middle: dynamic moduli, G’ and G”; bottom: tan(δ).

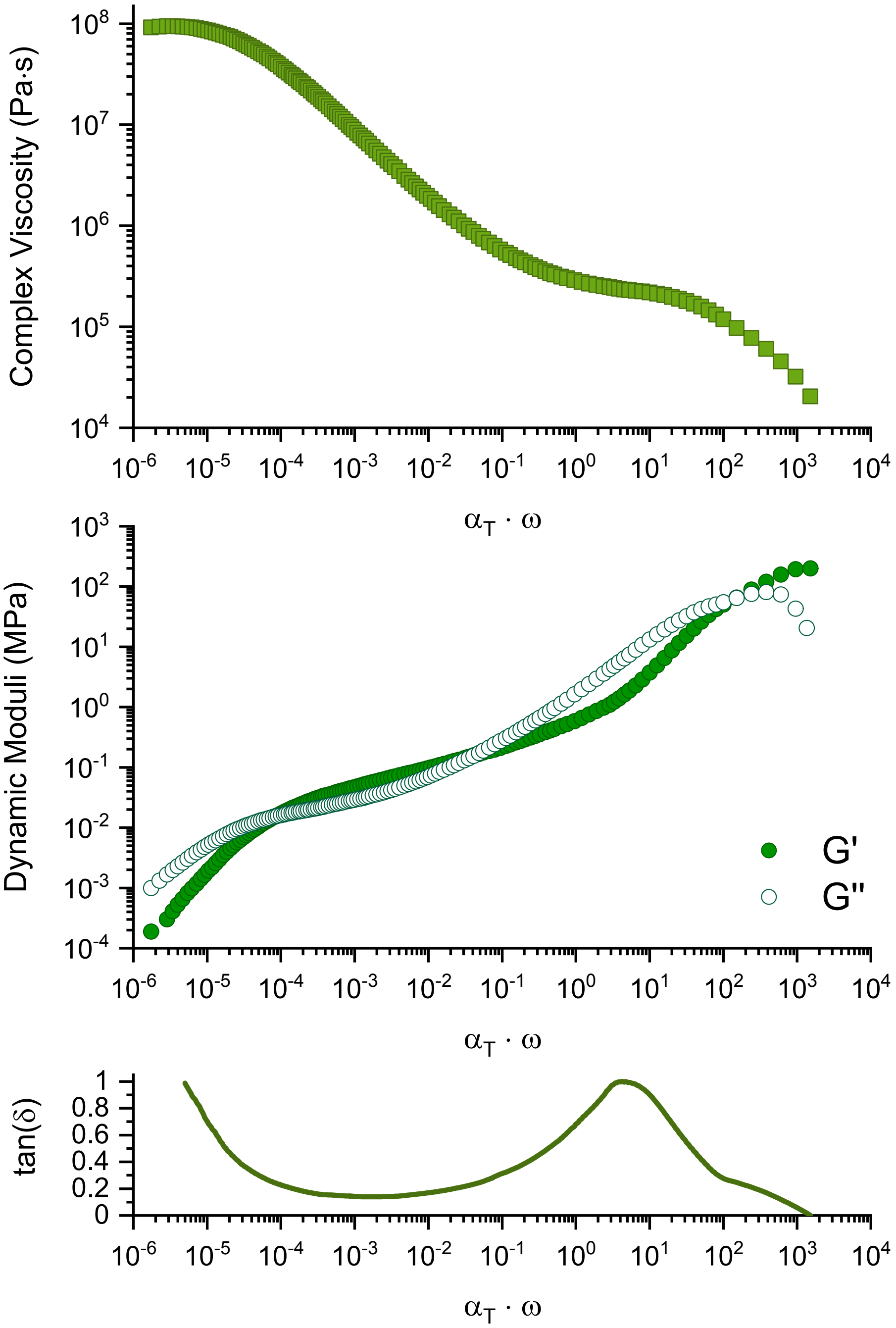

Figure S38: Mastercurves of PCHC-122 constructed by time-temperature superposition (TTS) referenced to 140 °C (0.5% strain, 0.01–100 Hz). The data points are the geometric average of four identical runs. Top: complex viscosity, η*; middle: dynamic moduli, G' and G"; bottom: tan(δ).

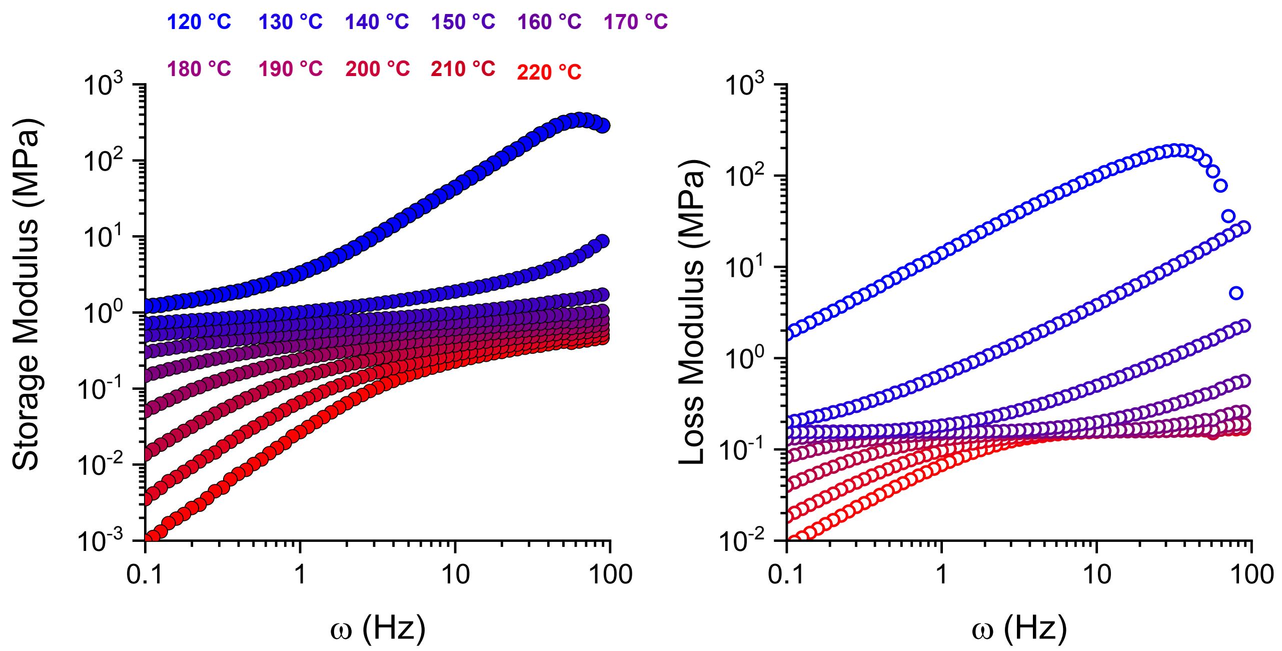

Figure S39: Oscillatory frequency sweeps (0.5% strain) of PCPC-114 employed in the creation of mastercurves.

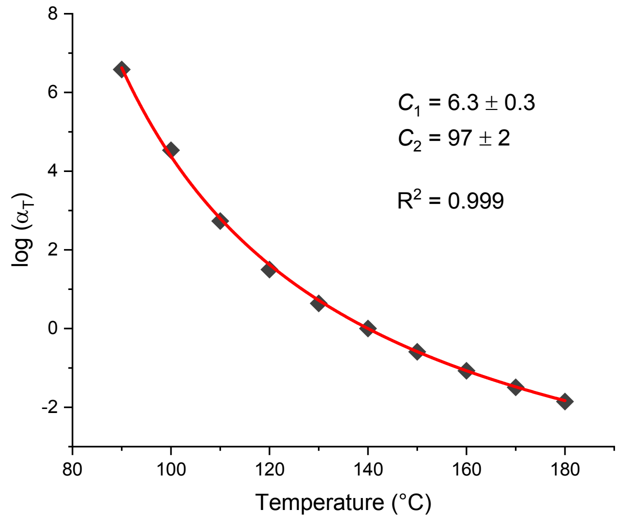

Figure S40: Temperature dependence of the shift factors, α_T, employed for the construction of the mastercurve of PCPC-114 (black diamonds) referenced at 140 °C and fitted curve (red line) to the WLF equation.

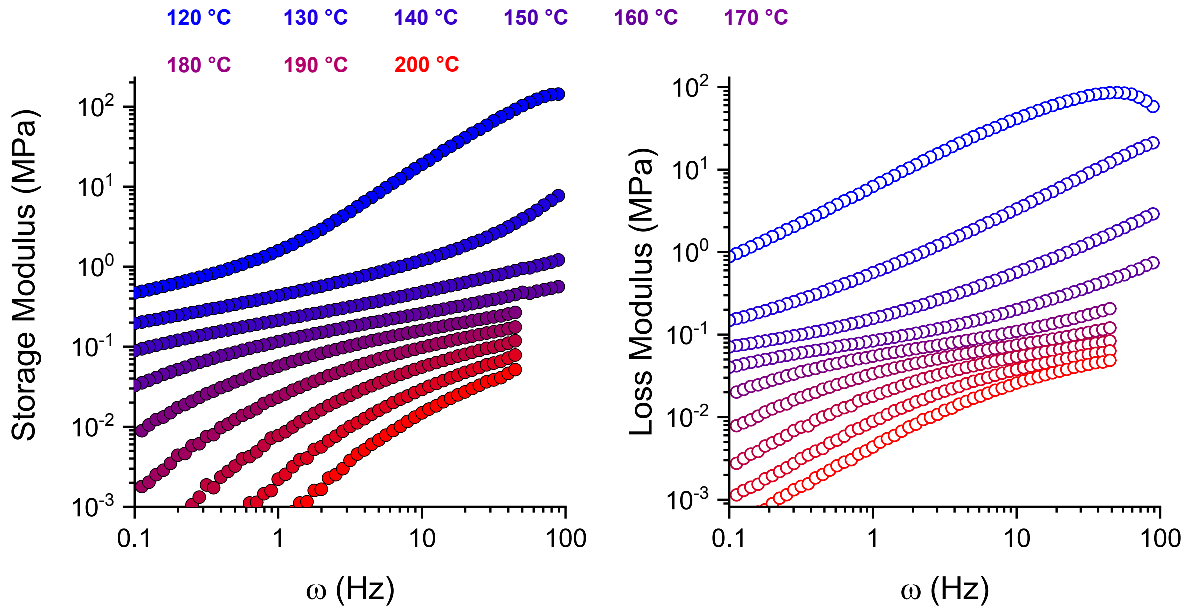

Figure S41: Oscillatory frequency sweeps (0.5% strain) of PeCHC-125 employed in the creation of mastercurves.

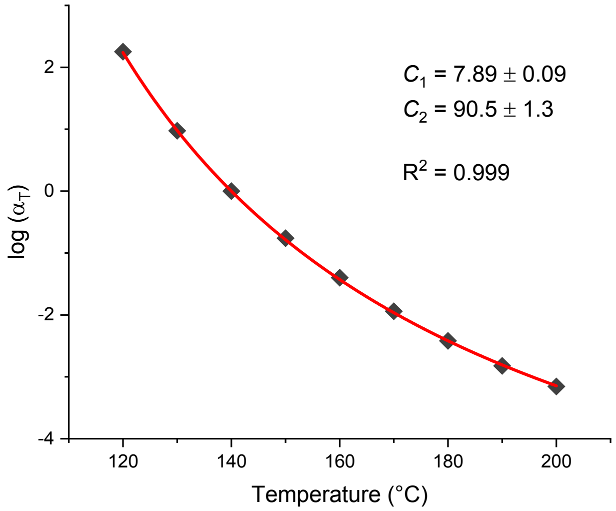

Figure S42: Temperature dependence of the shift factors, α_T, employed for the construction of the mastercurve of PeCHC-125 (black diamonds) referenced at 140 °C and fitted curve (red line) to the WLF equation.

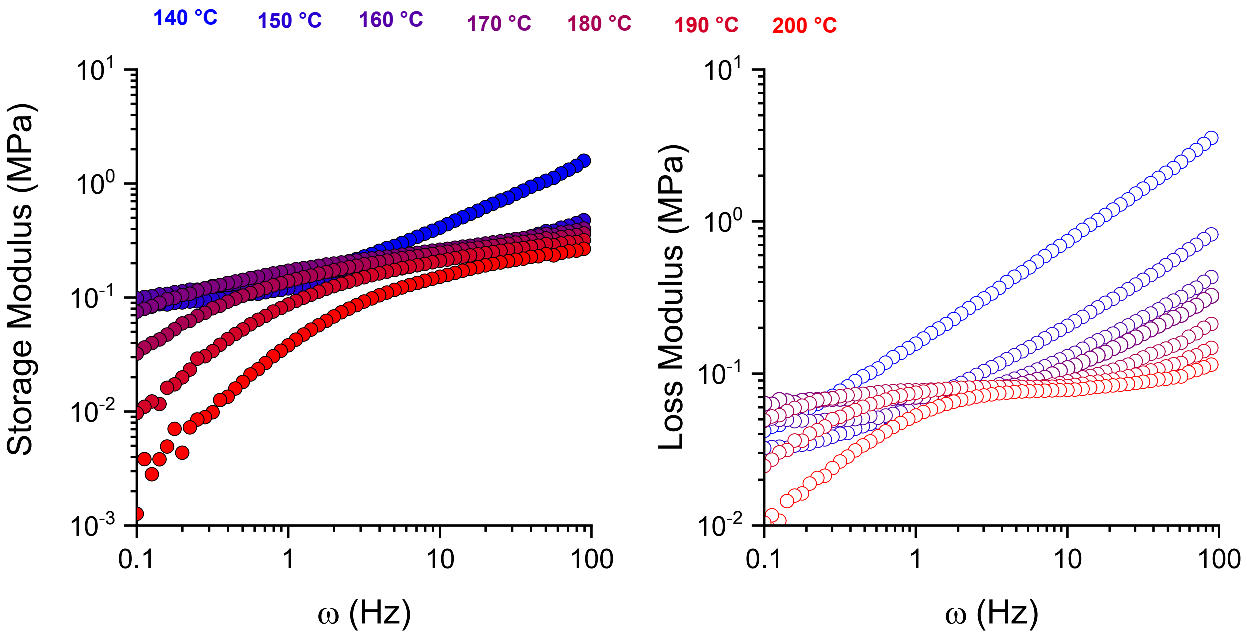

Figure S43: Oscillatory frequency sweeps (0.5% strain) of PvCHC-125 containing 0.1 wt% of a thermal stabilizer employed in the creation of mastercurves.

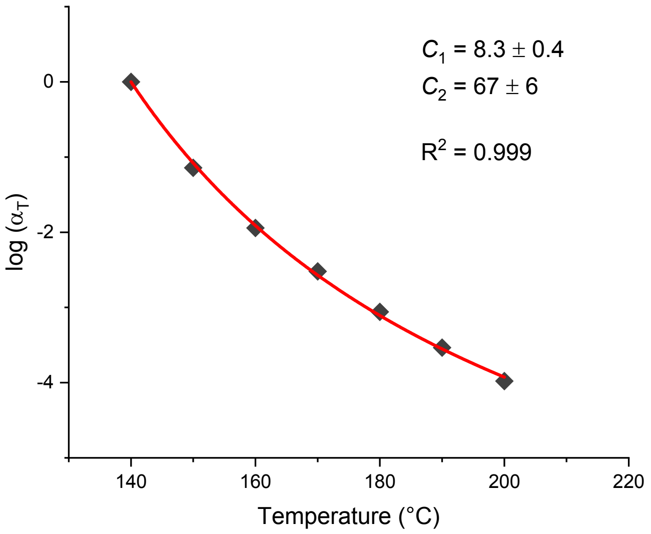

Figure S44: Temperature dependence of the shift factors, α_T, employed for the construction of the mastercurve of PvCHC-125 containing 0.1 wt% of a thermal stabilizer (black diamonds) referenced at 140 °C and fitted curve (red line) to the WLF equation.

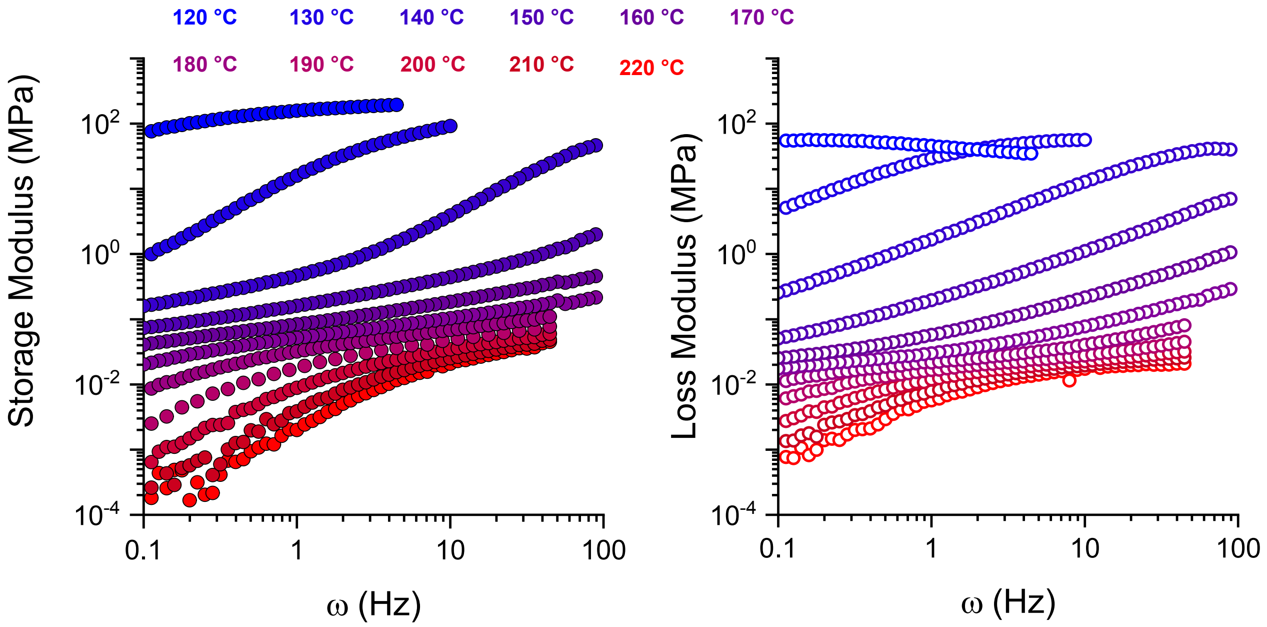

Figure S45: Oscillatory frequency sweeps (0.5% strain) of PCHC-122 employed in the creation of mastercurves.

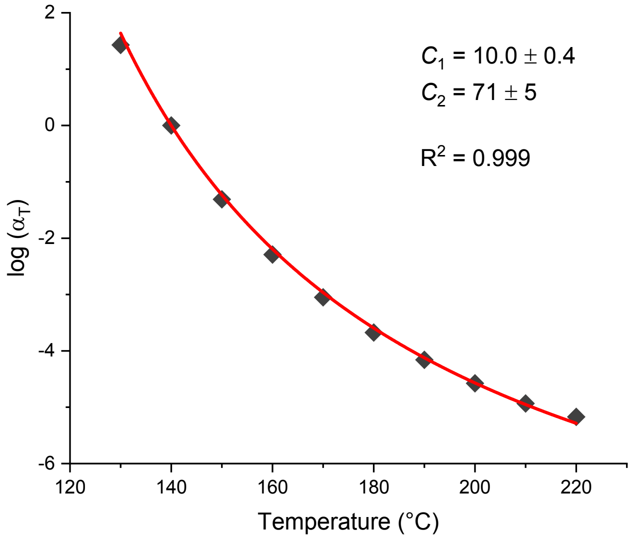

Figure S46: Temperature dependence of the shift factors, α_T, employed for the construction of the mastercurve of PCHC-122 (black diamonds) referenced at 140 °C and fitted curve (red line) to the WLF equation.

Thermal crosslinking of PvCHC. During the oscillatory temperature ramp, an unstabilized sample of vinyl substituted PvCHC-125, did not have a crossover temperature (Figure S48). Instead, its rubbery plateau extended well above 200 °C. It was proposed that some thermally induced cross-linking of the vinyl groups occurred at high temperatures. In support of this notion, PvCHC-125 samples subjected to DSC characterization in air showed an intense exothermic peak beginning at 173 °C which is consistent with alkene cross-linking (Figure S47). Moreover, stress-relaxation experiments using PvCHC-125 display step-wise increases in the relaxation modulus G(t)/G(0) with increasing temperature which is consistent with a cross-linked material. In contrast, PCPC-114 shows the viscous flow typical of a thermoplastic in related experiments (Figure S32). Further, PvCHC-125 treated at 180 °C for 2 hours would no longer dissolve in dioxane, instead forming a swollen gel, which is also typical of some chain crosslinking (Figure S51). The extent of cross-linking in the unstabilized PvCHC sample was probed by soaking a crosslinked film in a stock solution of catalyst **1** ($[\mathbf{1}]_0$:[PVCHC repeat units]$_0$) for 24 h, removing the solvent and subjecting the mixture to a 2 h isotherm at 140 °C on a TGA-FTIR (N_2 flow = 25 mL min^{-1}). 94% mass loss of the polymer was observed after 2 h, with vCHO and CO_2 detected as the sole products by FTIR (Figure S71 and 72, respectively). Under analogous conditions, non-crosslinked PvCHC degrades completely within 25 minutes. This suggests 6% mass is attributable to cross-linked moieties. As each cross-link involves two vCHC repeat units, it is posited approximately 3% of all vCHC repeat units were cross-linked under these conditions. This was further demonstrated with a scaled-up depolymerization: using 1.4 g of cross-linked PvCHC, the film was soaked in catalyst stock solution ($[\mathbf{1}]_0$:[PvCHC repeat units]$_0$, 1:300), the solvent removed to form a gel, and the mixture subjected to depolymerization at 140 °C *via* vacuum distillation. After 120 minutes, the gel had degraded to yield vCHO (92 %) as a clear oil. Notably, the bis-epoxide, i.e., the expected depolymerization product of the cross-linked polymer chains (Figure S72 and S73), was not detected. Accounting for some loss of vCHO in the distillation setup, this larger scale experiment is in reasonable agreement with the TGA-FTIR data indicating a cross-link density of <5%.

To address the issue of cross-linking, the PvCHC was processed with 0.1 wt% of a radical inhibitor, pentaerythritol tetrakis(3,5-di-tert-butyl-4-hydroxyhydrocinnamate) (PEHC), which is widely used to stabilize polymer formulations. Indeed, samples of PvCHC-125 containing 0.1 wt% of PEHC display the same high T_g value (129 °C, Figure S47) as the neat polymer and, importantly, show a crossover modulus at 196 °C (Figure S35). In the DSC experiments, this sample displays a substantially weaker exothermic peak at higher temperatures (onset at 213 °C), indicating more effective control over radical crosslinking.

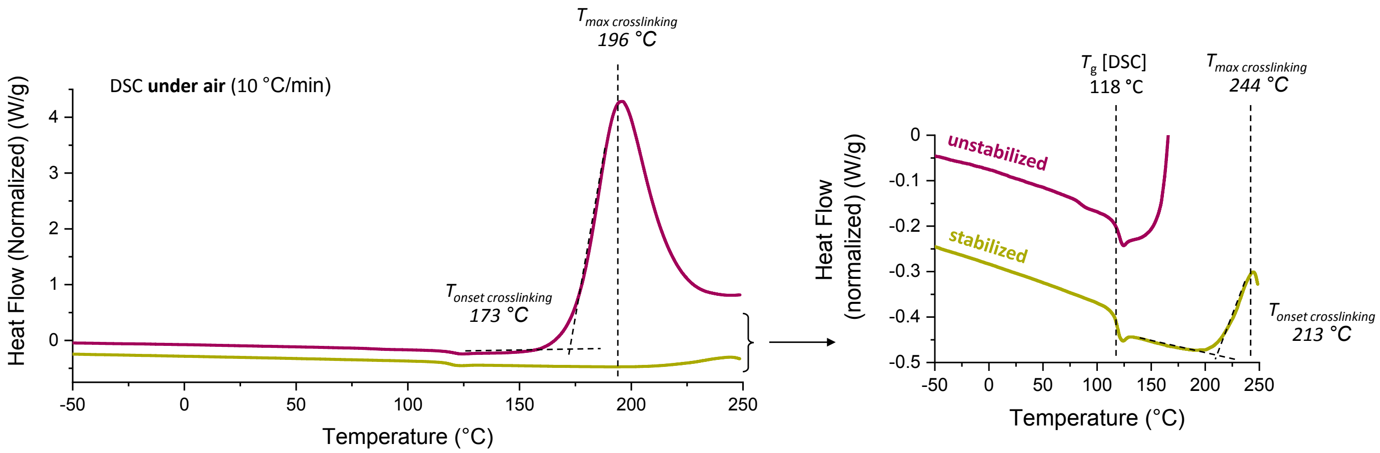

Figure S47: Comparison of thermal scans by DSC of PvCHC under air (10 °C min^{-1}): unstabilized sample (purple line) and stabilized sample (yellow line) by addition of 0.1 wt% of a radical inhibitor pentaerythritol tetrakis(3,5-di-tert-butyl-4-hydroxyhydrocinnamate).

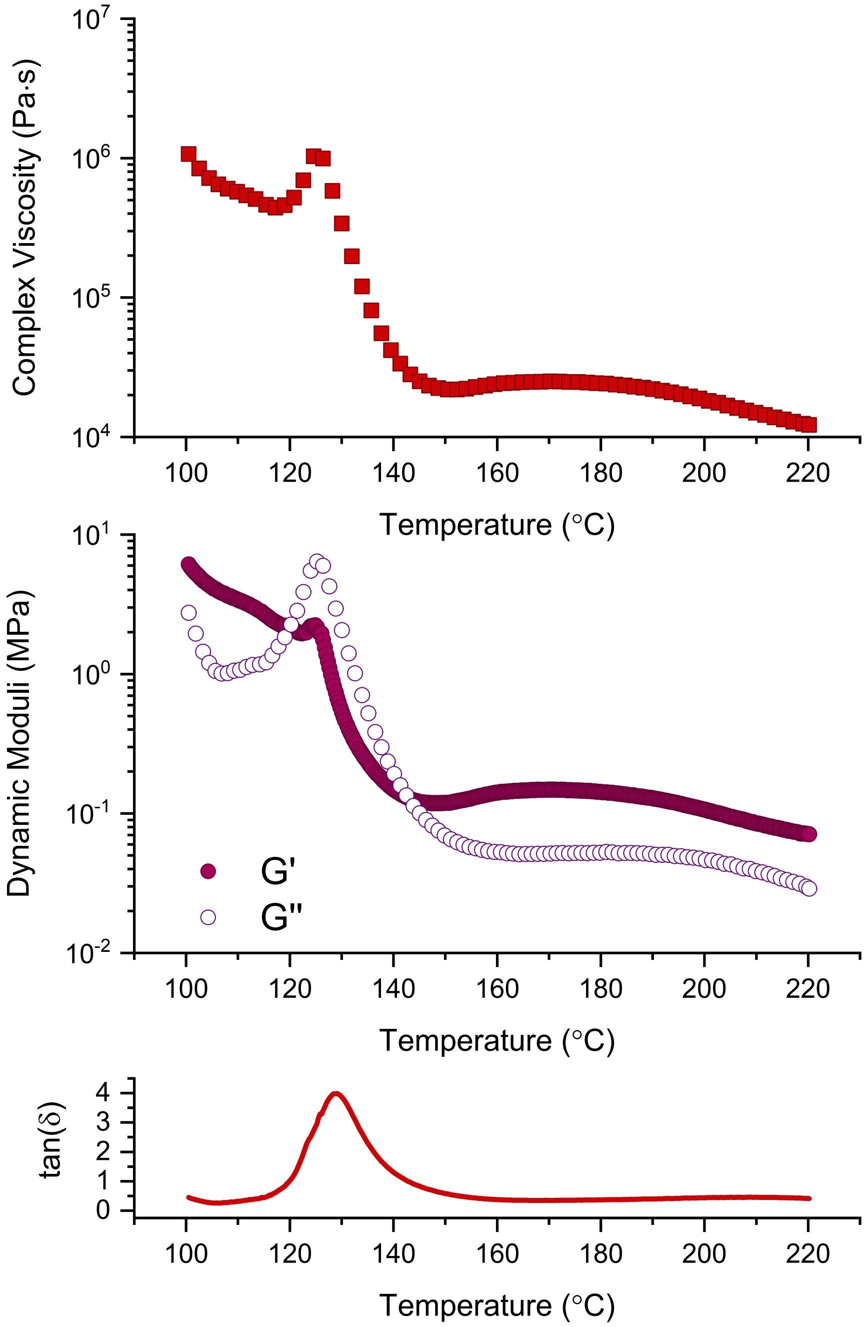

Figure S48: Oscillatory temperature ramp of unstabilized PvCHC-125 (0.5% strain, 1.0 Hz, 2 °C · min^{-1}) between 100 and 220 °C. Top: complex viscosity, η*; middle: dynamic moduli, G' and G"; bottom: tan(δ). The lack of crossover at high temperatures is expected to result from thermally induced cross-linking of the vinyl groups.

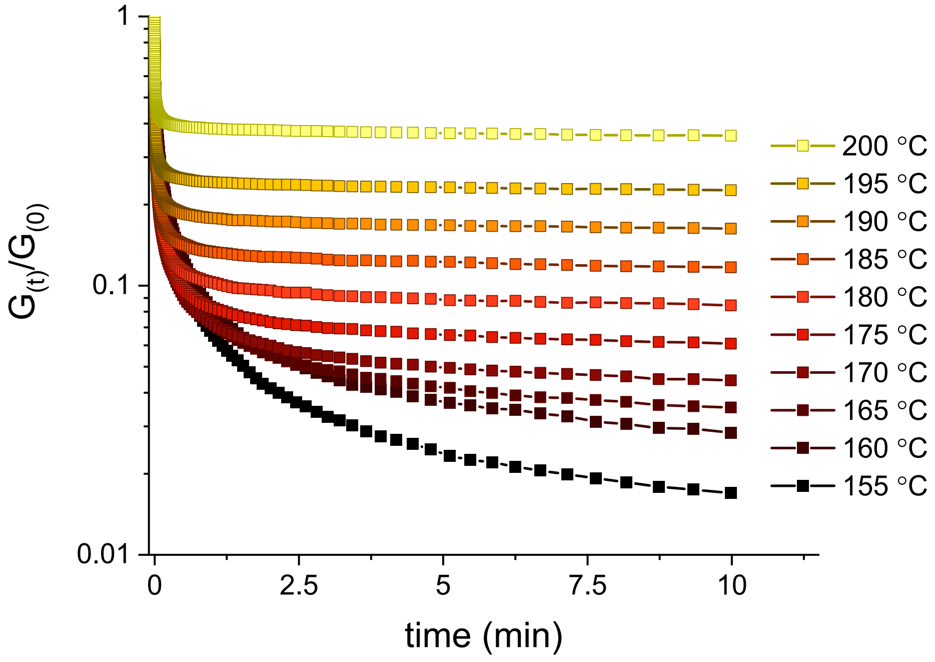

Figure S49: Normalized stress relaxation curves of unstabilized PvCHC-125 at various increasing temperatures.

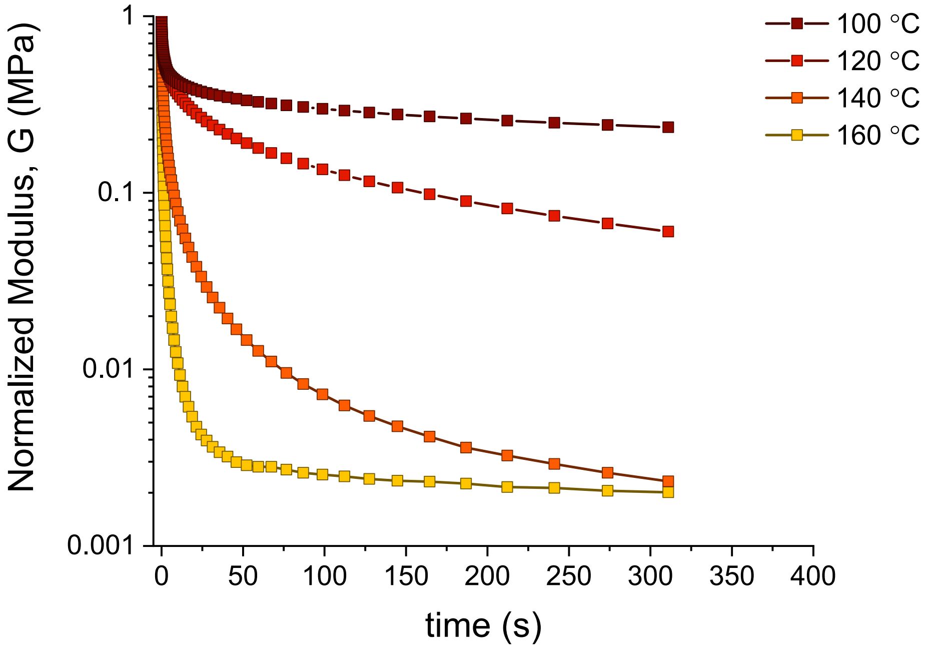

Figure S50: Normalized stress relaxation curves of PCPC-114 at various increasing temperatures.

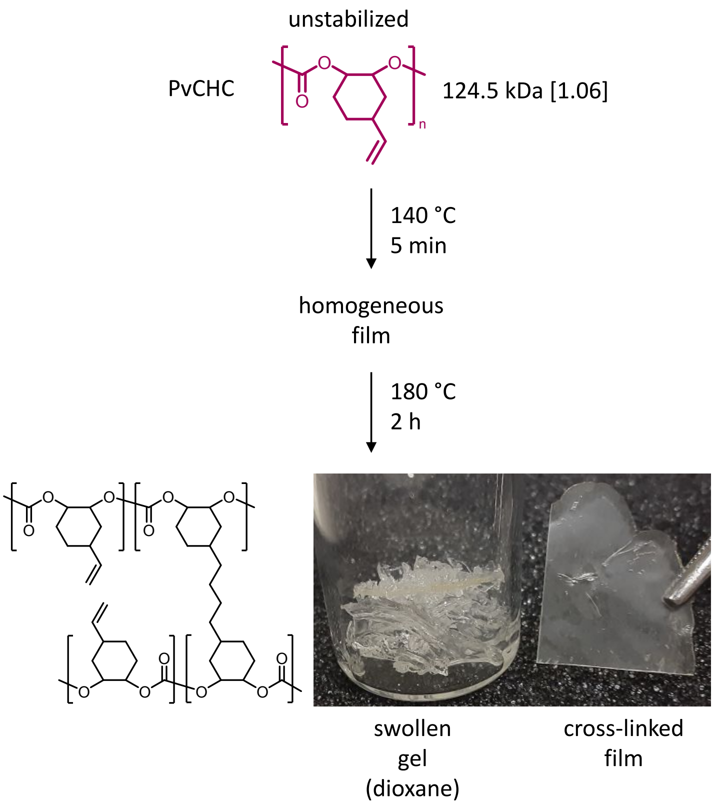

Figure S51: Diagram showing the cross-linking of a unstabilized PvCHC film sample. PvCHC-125 treated at 180 °C for 2 hours would no longer dissolve in dioxane, instead forming a swollen gel which is also typical of some chain crosslinking.

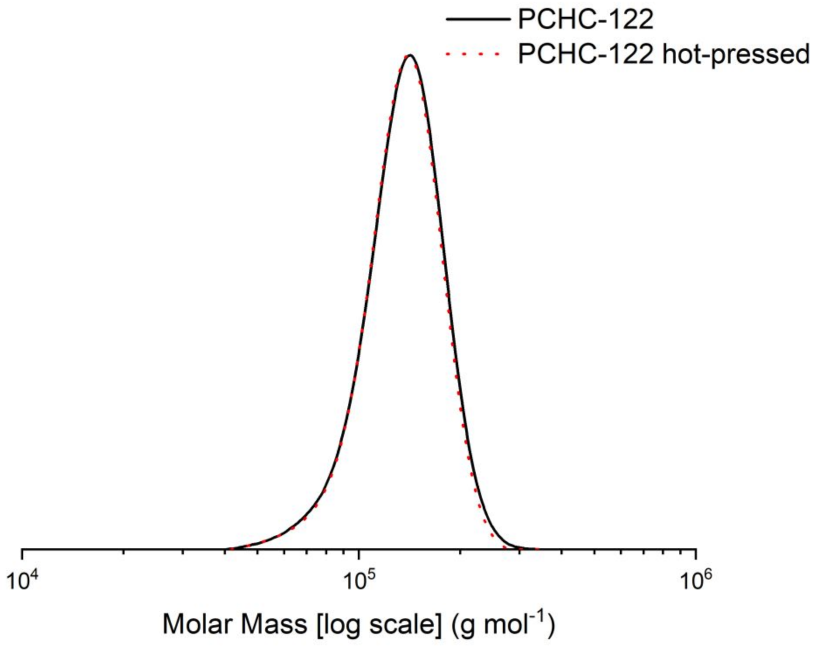

Figure S52: GPC analysis of PCHC-122 before and after hot-pressing.

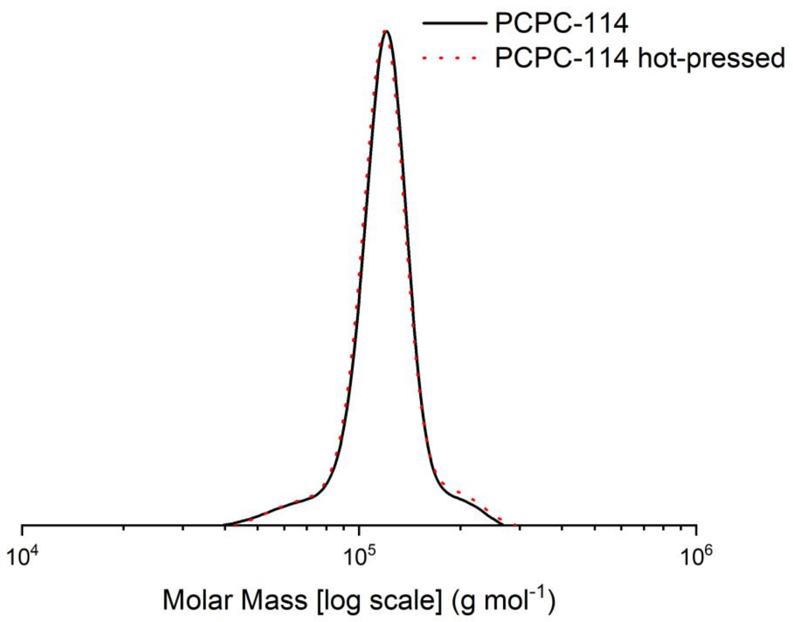

Figure S53: GPC analysis if PCPC-114 before and after hot pressing.

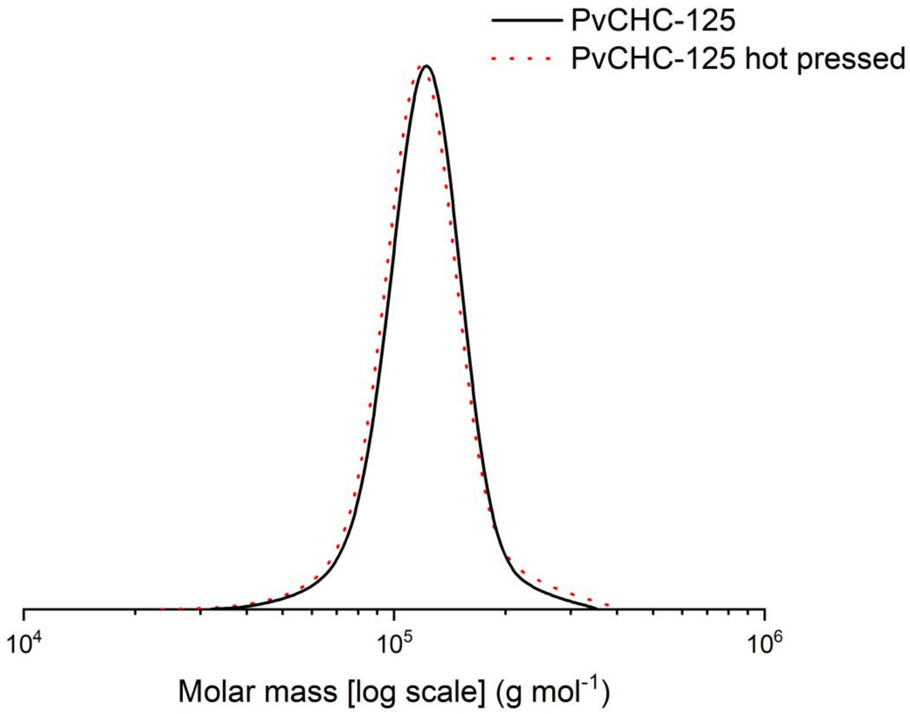

Figure S54: GPC analysis of PvCHC-125 before and after hot pressing (no stabilizer added).

Table S5: Thermal-mechanical properties of polymers in this work and commercial polymers.

Polymer	T_g (°C)[a]	σ (MPa)[b]	ε_b (%)[c]	E_y (GPa)[d]	u_t (MJ·m^{-3})[e]	Ref.
PvCHC-125	118	52.2 ± 1.0	4.9 ± 1.3	1.33 ± 0.11	1.6 ± 0.6	*this work*
PCHC-122	126	40.0 ± 1.8	3.3 ± 0.3	2.16 ± 0.06	0.9 ± 0.1	*this work*
PCPC-110	85	58.5 ± 1.7	7.1 ± 1.9	1.70 ± 0.09	2.9 ± 1.1	*this work*
PeCHC-125	105	46.7 ± 1.3	18.7 ± 4.2	1.31 ± 0.05	7.0 ± 1.5	*this work*
PvCHC-xlinked	N/A	38.7 ± 2.6	20.3 ± 5.0	1.81 ± 0.14	7.1 ± 1.9	*this work*
PLC	130	55	15	0.95	3.2	[16]
BPA-PC	147	63	45	2.5	22	[17]
PLLA	50-60	57.4 ± 3.7	9.3 ± 1.6	1.56 ± 0.22	4.0 ± 0.8	
PS	100	35-55	3.4	3.2-3.4	1.4 ± 0.3	[17]
PMMA	105	78.0 ± 7.3	8.5 ± 1.4	1.23 ± 0.04	3.89 ± 1.0	

[a]Determined by DSC at a scan rate of 10 °C min^{-1}. [b]Ultimate tensile strength. [c]Strain at break. [d]Young's Modulus. [e]Tensile toughness. Mean values ± std. dev. from measurements conducted independently on at least 4 specimens. PLC = poly(limonene carbonate).

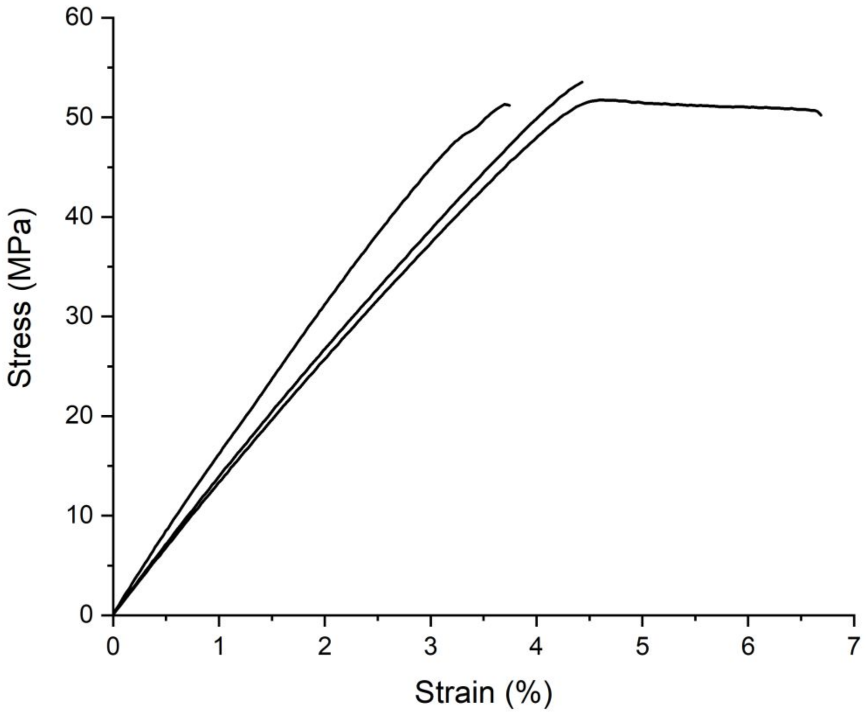

Figure S55: Tensile data for PvCHC-125 (without stabilizer).

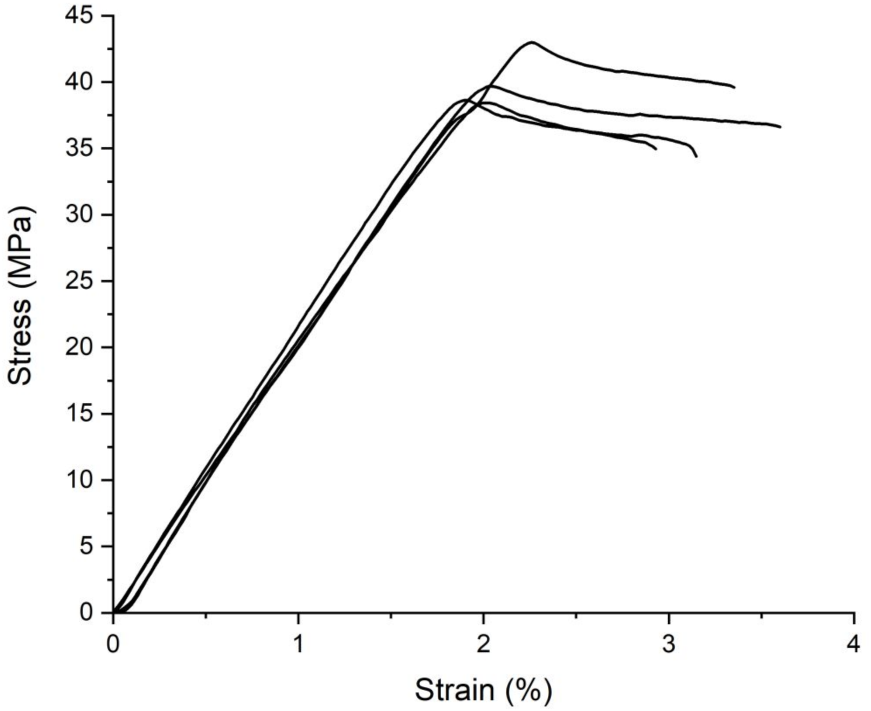

Figure S56: Tensile data for PCHC-122.

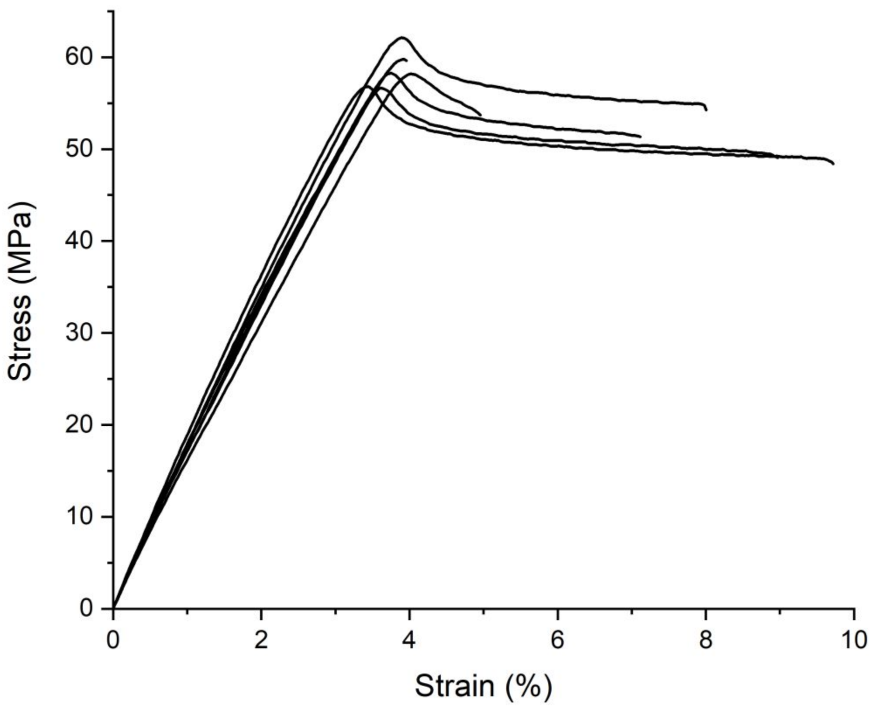

Figure S57: Tensile data for PCPC-114.

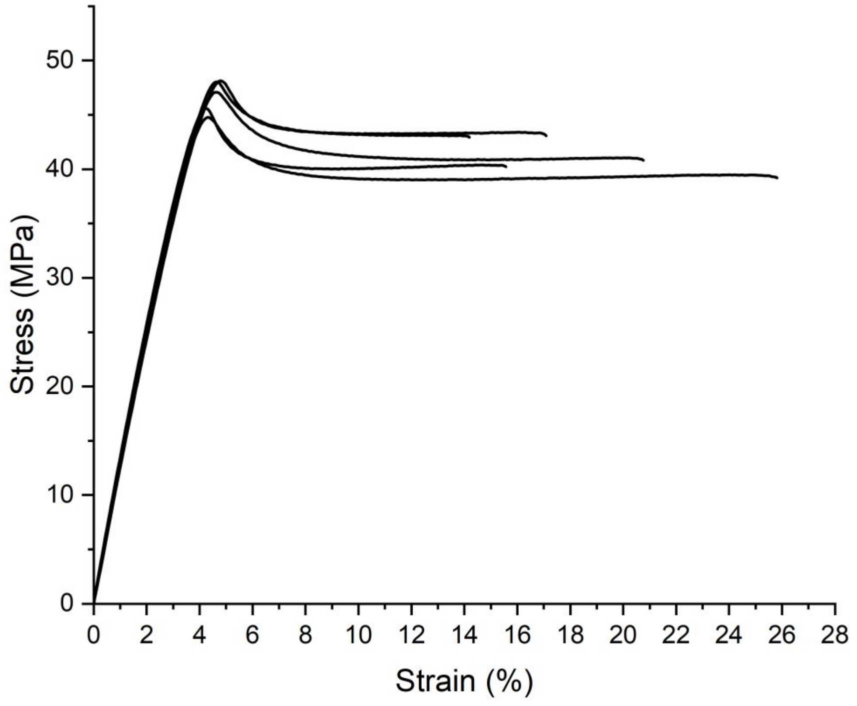

Figure S58: Tensile data for PeCHC-125.

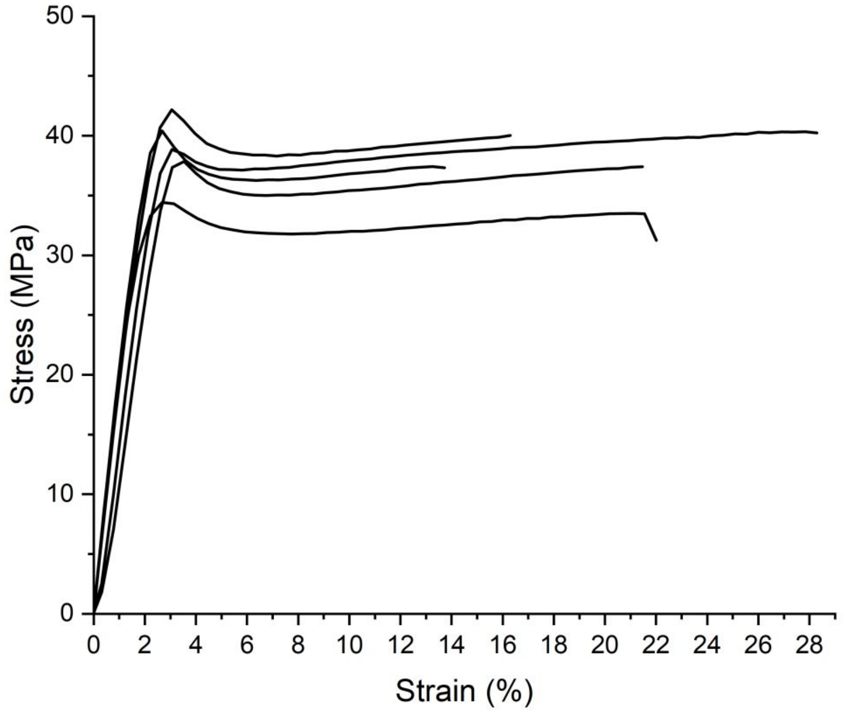

Figure S59: Tensile data for PvCHC-xlinked.

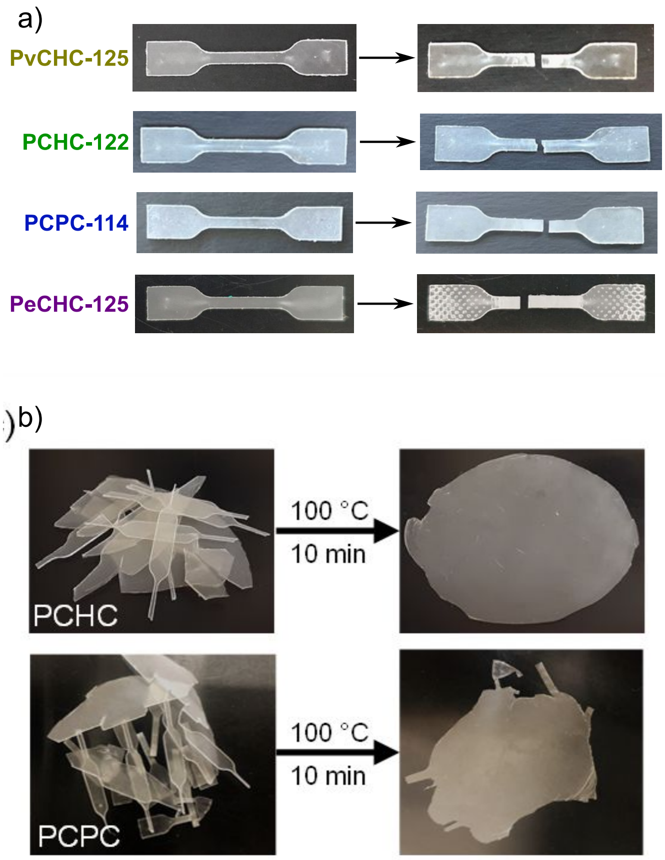

Figure S60: a) Images of the polymer specimens before and after tensile testing. b) Samples of PCHC-122 and PCPC-114 re-processed into a homogeneous film by compression moulding for 10 min at 140 °C and 100°C, respectively.

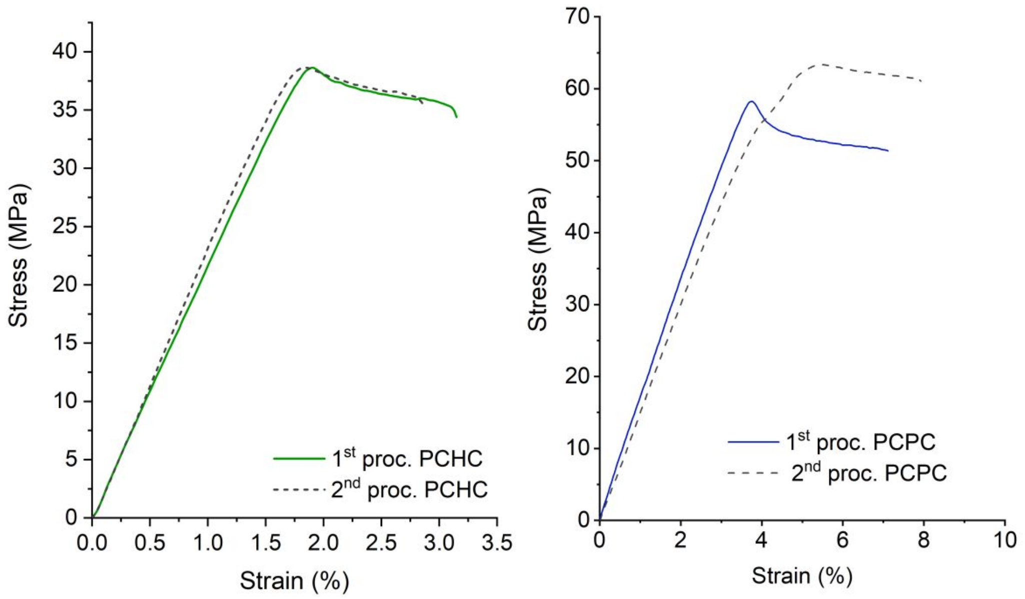

Figure S61: Tensile graphs of PCHC (left) and PCPC (right) specimens after reprocessing.

Table S6: Depolymerization data for PCHC, PCPC and PvCHC using catalyst 1 and prior literature reports on the depolymerization of PCPC.

entry #	polymer	$[cat]_0$:$[pol]_0$	k_{obs} (h^{-1})[a]	TOF (h^{-1})[b]	mass loss rate (kg g h^{-1})[c]	Selectivity (%)[d]
1	PCHC-122	1:300	4.1 (±0.3)	700 (±60)	0.11 (±0.01)	>99
2	PvCHC-125	1:300	4.4 (±0.2)	1700 (±60)	0.27 (±0.05)	>99
3	PCPC-114	1:300	10.7(±0.6)	4400 (±490)	0.59 (±0.06)	>99
4	XL-PvCHC	1:300	3.7 (±0.1)	490 (±60)	0.09 (±0.01)	>99
5	PeCHC-125	1:300	2.8 (±0.2)	540 (±70)	0.10 (±0.01)	>99
6	PCPC-114	1:1000	2.1 (±0.6)	1600 (±180)	0.17 (±0.01)	>99
7	PCPC-114	1:5000	1.1 (±0.1)	2500 (±200)	0.32 (±0.05)	>99
8 [e]	PCPC-45	1:50	Nd	3	0.00072	>99
9 [f]	PCPC-21	1:500	Nd	705	0.074	94
10 [g]	PCPC	1:50	Nd	2	0.00021	90

Depolymerizations conducted using TGA measurements, under N_2 (flow rate = 25 mL min^{-1}), at 140 °C, unless otherwise stated. Loadings given with respect to the polymer repeat unit. [a] k_{obs} calculated from first-order exponential decay fits of mass% *vs.* time plots, errors calculated from repeat runs.[b] TOF = moles of polymer repeat unit consumed (20-80% conversion)/mole of catalyst/time, unless otherwise stated.[c] Mass loss rate = mass of polymer repeat unit consumed (20-80% conversion)/mass of catalyst/time, unless otherwise stated.[d] Determined by *in-situ* TGA-FTIR measurements.[e] Taken from Liu *et al*,[18] Table 2, entry 9, conversion = 63% after 12 h, reaction performed at 110 °C in toluene with $[PCPC]_0$ = 1.56 M in toluene. Conversion and selectivity determined by ^{1}H NMR spectroscopy. [f] Taken from Lu et al,[19] Figure 3, conversion 99%, selectivity 94% after 40 minutes, reaction performed at 200 °C in bulk PCPC. Conversion and selectivity determined by ^{1}H NMR spectroscopy. [g] Taken from Darensbourg *et al*,[20] Table 1 entry 1, conversion = 76% after 19 h, reaction performed at 110 °C in *d*-toluene with $[PCPC]_0$ = 0.3 M. Conversion and selectivity determined by ^{1}H NMR spectroscopy.

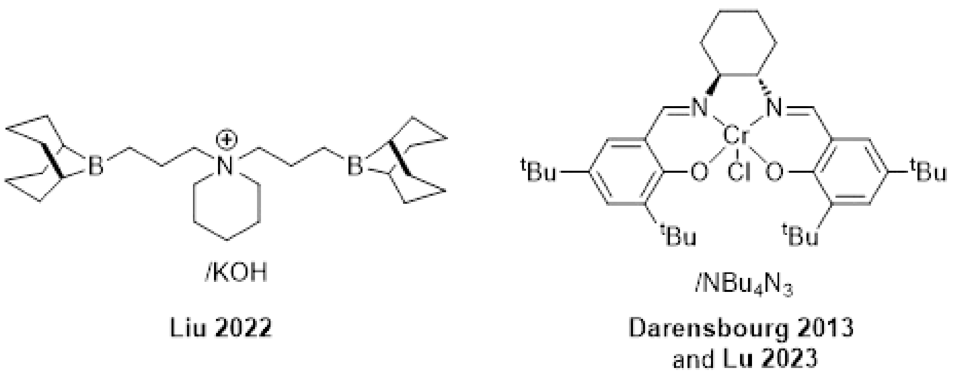

Figure S62. Structures of catalysts used for the depolymerization of PCPC.[18-20]

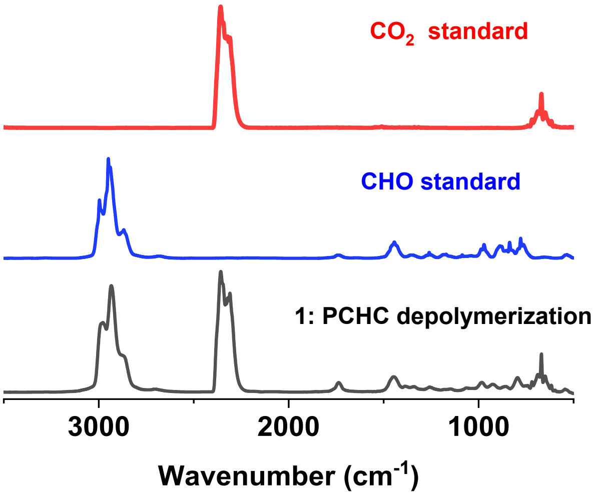

Figure S63. TGA-FTIR gas-phase analysis of PCHC-122 depolymerization mixture catalysed by 1. ([1]$_0$:[PCHC-122]$_0$ = 1:300, 140 °C, black) with stacked CHO (blue) and CO_2 standards (red).

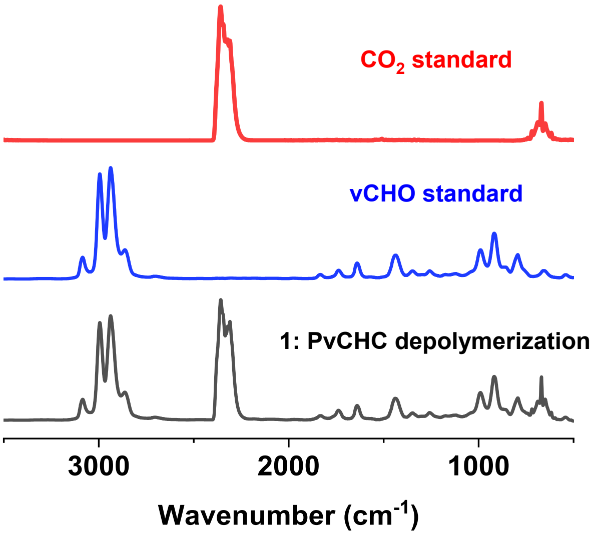

Figure S64. TGA-FTIR gas-phase analysis of PvCHC-125 depolymerization mixture catalysed by 1 ([1]$_0$:[PvCHC-125]$_0$ = 1:300, 140 °C, black) with stacked vCHO (blue) and CO_2 standards (red).

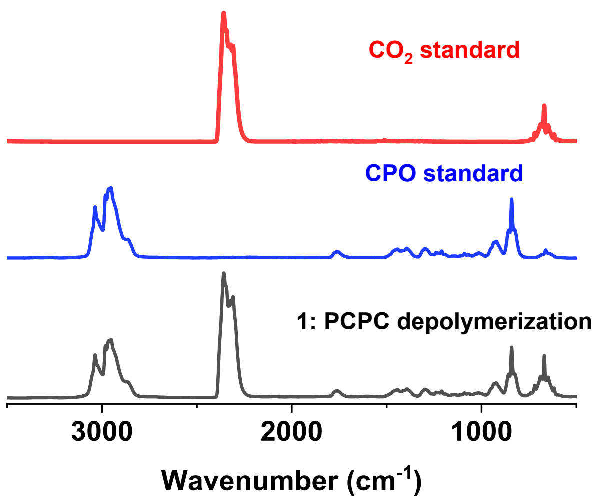

Figure S65. TGA-FTIR gas-phase analysis of PCPC-114 depolymerization mixture catalysed by **1**. ($[1]_0$:$[\text{PCPC-114}]_0$ = 1:300, 140 °C, black) with stacked CPO (blue) and CO_2 standards (red).

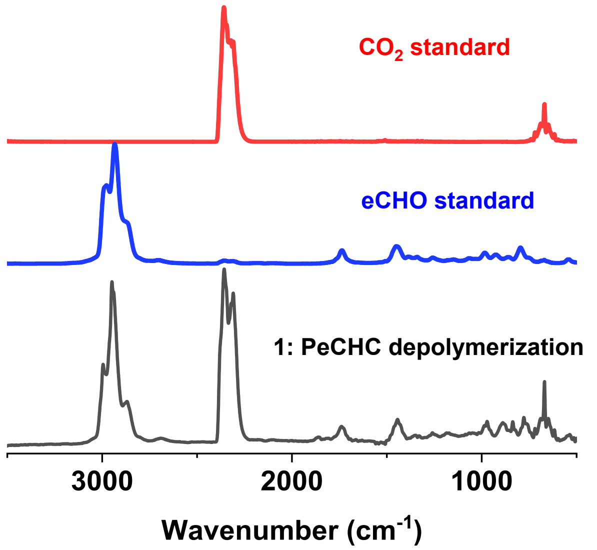

Figure S66. TGA-FTIR gas-phase analysis of PeCHC-125 depolymerization mixture catalysed by **1**. ($[1]_0$:$[\text{PeCHC-125}]_0$ = 1:300, 140 °C, black) with stacked eCHO (blue) and CO_2 standards (red).

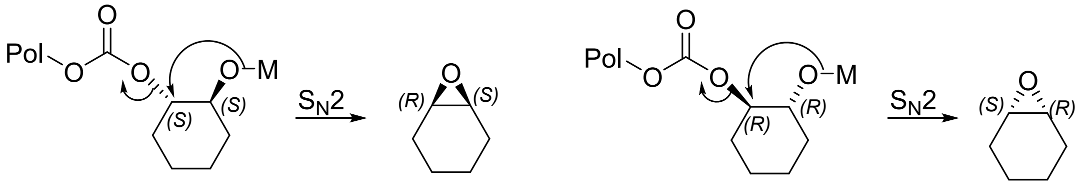

Figure S67: Example depolymerization mechanism showing backbiting reactions with stereochemical inversion to form *cis*-epoxide, irrespective of absolute stereochemistry.

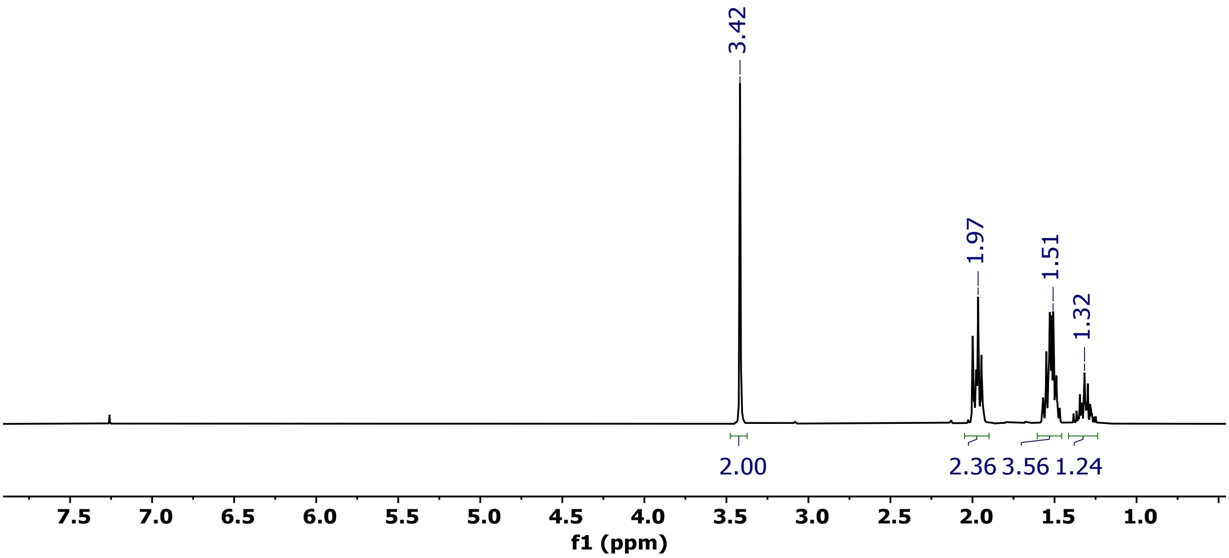

Figure S68: ^{1}H NMR spectrum ($CDCl_3$) of CPO isolated from the depolymerization of PCPC-114.The reaction was conducted at 140 °C with $[cat]_0$: $[PCPC\text{-}114]_0$ = 1: 1000.

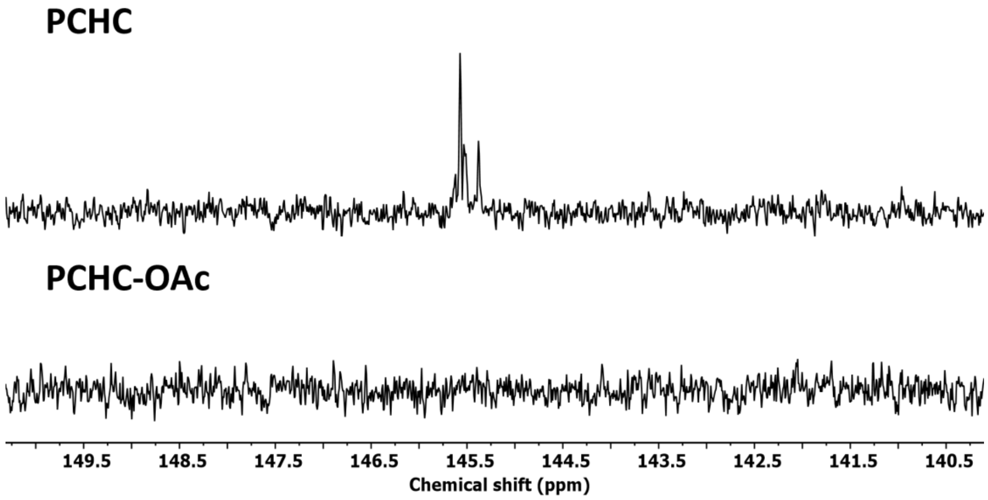

Figure S69: End group analysis of PCHC and PCHC-OAc by $^{31}P\{^1H\}$ NMR spectroscopy, after reacting with 2-chloro-4,4,5,5-tetramethyl dioxaphospholane in $CDCl_3$. The peak at 145.5 ppm for PCHC is indicative of hydroxyl end groups, whereas the absence of peaks in PCHC-OAc means that there are no residual hydroxyl moieties.

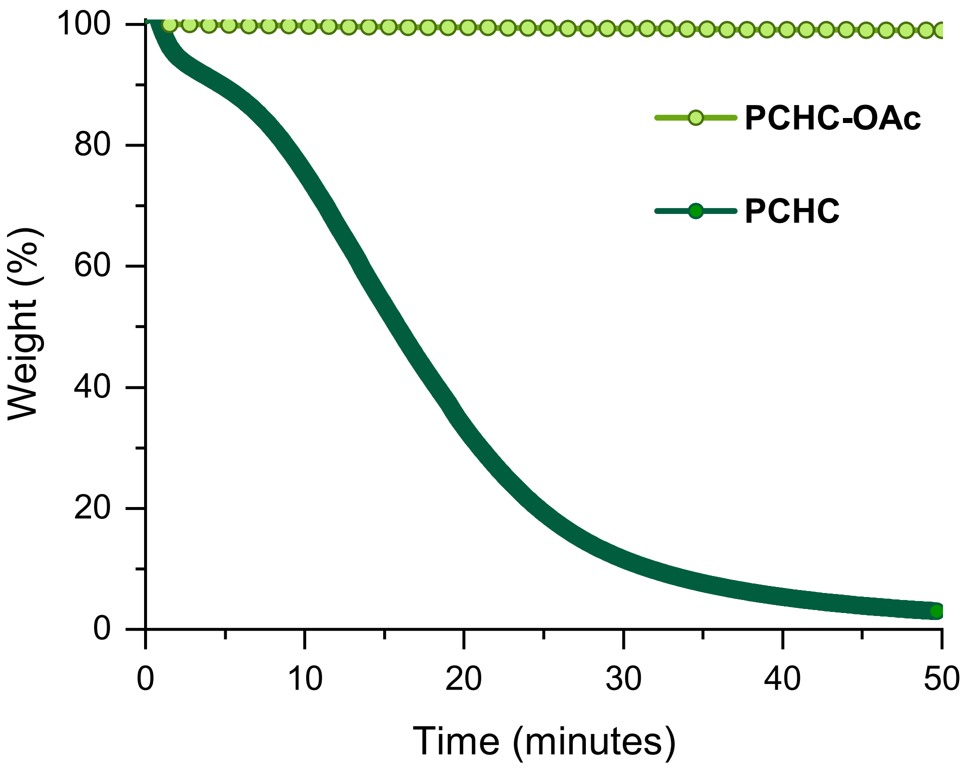

Figure S70: Isothermal TGA data at 140 °C comparing the depolymerization of the PCHC-OAc and PCHC in the presence of complex **1** ($[1]_0$:$[polymer]_0$ = 1:300).

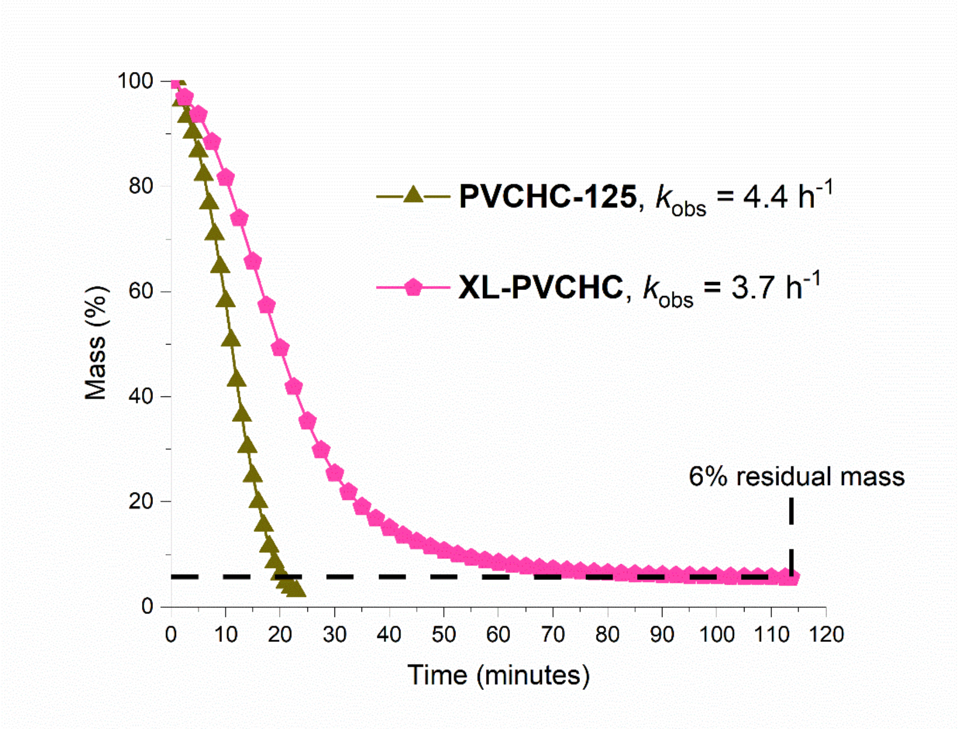

Figure S71. Representative weight vs. time plots for the depolymerization of PvCHC-125 (yellow), PVCHC x-linked (pink) catalysed by **1**.The depolymerizations were performed at 140 °C with $[1]_0$:$[polymer]_0$ loadings of 1:300.

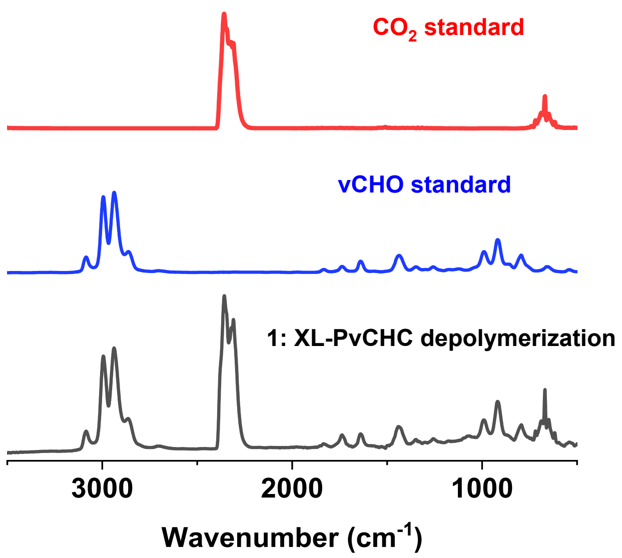

Figure S72. TGA-FTIR gas-phase analysis of PvCHC x-linked depolymerization mixture catalysed by **1.** ($[1]_0$:[PvCHC x-linked]$_0$ = 1:300, 140 °C, black) with stacked vCHO (blue) and CO_2 standards (red).

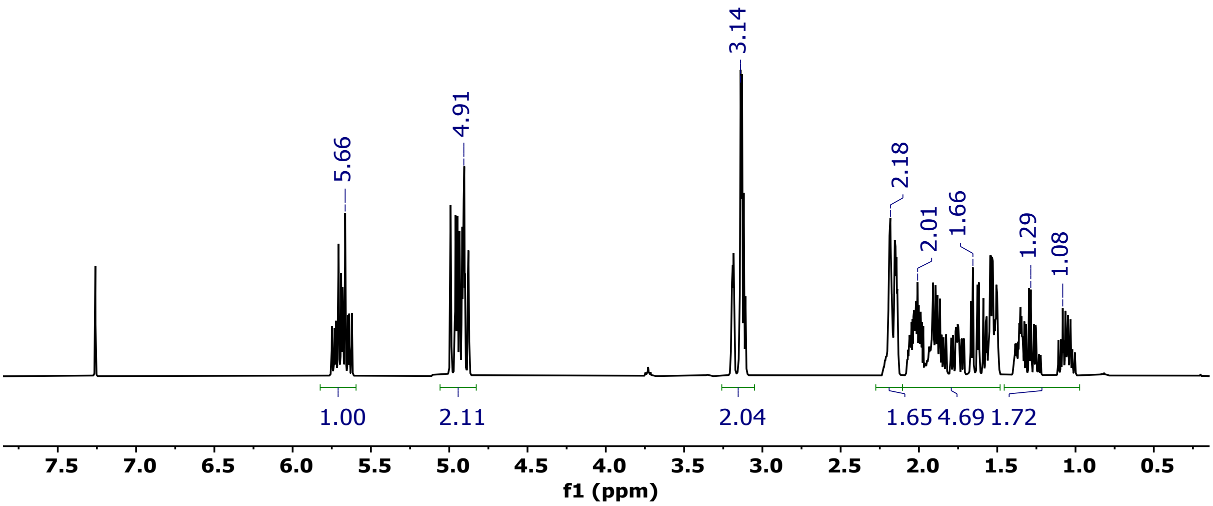

Figure S73: ^{1}H NMR spectrum ($CDCl_3$) of vCHO isolated from the depolymerization of PvCHC-x-linked. The reaction was conducted at 140 °C with $[cat]_0$: [PVCHC x-linked]$_0$ = 1: 300.

References

(1) Kember, M. R.; Knight, P. D.; Reung, P. T. R.; Williams, C. K., Highly Active Dizinc Catalyst for the Copolymerization of Carbon Dioxide and Cyclohexene Oxide at One Atmosphere Pressure. *Angew. Chem. Int. Ed.* **2009,** *48* (5), 931-933. 10.1002/anie.200803896.

(2) Royo, P.; Vazquez, A., Pentafluorophenylcobalt(II) complexes. *J. Organomet. Chem.* **1981,** *204* (2), 243-247. https://doi.org/10.1016/S0022-328X(00)84590-8.

(3) Smith, C. F.; Tamborski, C., Synthesis and reactions of a bis(pentafluorophenyl)cobalt complex. *J. Organomet. Chem.* **1971,** *32* (2), 257-262. https://doi.org/10.1016/S0022-328X(00)85078-0.

(4) Deacy, A. C.; Kilpatrick, A. F. R.; Regoutz, A.; Williams, C. K., Understanding metal synergy in heterodinuclear catalysts for the copolymerization of CO2 and epoxides. *Nat. Chem.* **2020,** *12* (4), 372-380. 10.1038/s41557-020-0450-3.

(5) Cosier, J.; Glazer, A., A nitrogen-gas-stream cryostat for general X-ray diffraction studies. *J. Appl. Crystallogr.* **1986,** *19* (2), 105-107. doi:10.1107/S0021889886089835.

(6) CrysAlisPRO, Oxford Diffraction /Agilent Technologies UK Ltd, Yarnton, England.

(7) SHELXTL v5.1, Bruker AXS, Madison, WI, **1998**.

(8) Dolomanov, O. V.; Bourhis, L. J.; Gildea, R. J.; Howard, J. A. K.; Puschmann, H., OLEX2: a complete structure solution, refinement and analysis program. *J. Appl. Crystallogr.* **2009,** *42* (2), 339-341. doi:10.1107/S0021889808042726.

(9) Sheldrick, G., Crystal structure refinement with SHELXL. *Acta Crystallogr. Sect. C* **2015,** *71* (1), 3-8. doi:10.1107/S2053229614024218.

(10) Diment, W. T.; Williams, C. K., Chain end-group selectivity using an organometallic Al(iii)/K(i) ring-opening copolymerization catalyst delivers high molar mass, monodisperse polyesters. *Chem. Sci.* **2022,** *13* (29), 8543-8549. 10.1039/D2SC02752F.

(11) Hillmyer, M. A.; Laredo, W. R.; Grubbs, R. H., Ring-Opening Metathesis Polymerization of Functionalized Cyclooctenes by a Ruthenium-Based Metathesis Catalyst. *Macromolecules* **1995,** *28* (18), 6311-6316. 10.1021/ma00122a043.

(12) Li, C.; Sablong, R. J.; van Benthem, R. A. T. M.; Koning, C. E., Unique Base-Initiated Depolymerization of Limonene-Derived Polycarbonates. *ACS Macro Lett.* **2017,** *6* (7), 684-688. 10.1021/acsmacrolett.7b00310.

(13) Cheng, M.; Darling, N. A.; Lobkovsky, E. B.; Coates, G. W., Enantiomerically-enriched organic reagents polymer synthesis: enantioselective copolymerization of cycloalkene oxides and CO using homogeneous, zinc-based catalysts. *Chem. Commun.* **2000,** (20), 2007-2008. 10.1039/B005537I.

(14) Koning, C.; Wildeson, J.; Parton, R.; Plum, B.; Steeman, P.; Darensbourg, D. J., Synthesis and physical characterization of poly(cyclohexane carbonate), synthesized from CO2 and cyclohexene oxide. *Polymer* **2001,** *42* (9), 3995-4004. https://doi.org/10.1016/S0032-3861(00)00709-6.

(15) Fetters, L. J.; Lohse, D. J.; Richter, D.; Witten, T. A.; Zirkel, A., Connection between Polymer Molecular Weight, Density, Chain Dimensions, and Melt Viscoelastic Properties. *Macromolecules* **1994,** *27* (17), 4639-4647. 10.1021/ma00095a001.
(16) Hauenstein, O.; Reiter, M.; Agarwal, S.; Rieger, B.; Greiner, A., Bio-based polycarbonate from limonene oxide and CO2 with high molecular weight, excellent thermal resistance, hardness and transparency. *Green Chem.* **2016,** *18* (3), 760-770. 10.1039/C5GC01694K.
(17) CHEMnetBASE - Polymers: a Property Database. http://poly.chemnetbase.com/faces/polymers/PolymerSearch.xhtml (accessed Feb 22).
(18) Yang, G.-W.; Wang, Y.; Qi, H.; Zhang, Y.-Y.; Zhu, X.-F.; Lu, C.; Yang, L.; Wu, G.-P., Highly Selective Preparation and Depolymerization of Chemically Recyclable Poly(cyclopentene carbonate) Enabled by Organoboron Catalysts. *Angew. Chem. Int. Ed.* **2022,** *61* (46), e202210243. https://doi.org/10.1002/anie.202210243.
(19) Yu, Y.; Gao, B.; Liu, Y.; Lu, X.-B., Efficient and Selective Chemical Recycling of CO2-Based Alicyclic Polycarbonates via Catalytic Pyrolysis. *Angew. Chem. Int. Ed.* **2022,** *61* (34), e202204492. https://doi.org/10.1002/anie.202204492.
(20) Darensbourg, D. J.; Wei, S.-H.; Yeung, A. D.; Ellis, W. C., An Efficient Method of Depolymerization of Poly(cyclopentene carbonate) to Its Comonomers: Cyclopentene Oxide and Carbon Dioxide. *Macromolecules* **2013,** *46* (15), 5850-5855. 10.1021/ma401286x.
